# Identification of miRNAs and Their Target Genes Associated with Sunitinib Resistance in Clear Cell Renal Cell Carcinoma Patients

**DOI:** 10.3390/ijms25136881

**Published:** 2024-06-22

**Authors:** María Armesto, Stéphane Nemours, María Arestín, Iraide Bernal, Jon Danel Solano-Iturri, Manuel Manrique, Laura Basterretxea, Gorka Larrinaga, Javier C. Angulo, David Lecumberri, Ane Miren Iturregui, José I. López, Charles H. Lawrie

**Affiliations:** 1 Molecular Oncology Group, Biogipuzkoa Health Research Institute, 20014 San Sebastián, Spain; maria.armestoalvarez@bio-gipuzkoa.eus (M.A.); stephane.nemours@bio-gipuzkoa.eus (S.N.); maria.arestinmuruzabal@bio-gipuzkoa.eus (M.A.); iraide.bernalsimon@osakidetza.eus (I.B.); laura.basterrecheabadiola@osakidetza.eus (L.B.); 2 Pathology Department, Donostia University Hospital, 20014 San Sebastián, Spain; jondanel.solanoiturri@osakidetza.eus (J.D.S.-I.); manuel.manriquecelada@osakidetza.eus (M.M.); 3 Medical Oncology Department, Donostia University Hospital, 20014 San Sebastián, Spain; 4 Biobizkaia Health Research Institute, 48903 Barakaldo, Spain; gorka.larrinaga@ehu.eus (G.L.); joseignacio.lopez@biocrucesbizkaia.org (J.I.L.); 5 Department of Physiology, Faculty of Medicine and Nursing, University of the Basque Country (UPV/EHU), 48940 Leioa, Spain; 6 Clinical Department, Faculty of Medical Sciences, European University of Madrid, 28905 Getafe, Spain; javier.angulo@universidadeuropea.es; 7 Department of Urology, University Hospital of Getafe, 28907 Madrid, Spain; 8 Department of Urology, Urduliz University Hospital, 48610 Urduliz, Spain; david.lecumberricastanos@osakidetza.eus; 9 Department of Urology, Cruces University Hospital, 48903 Barakaldo, Spain; anemiren.iturreguidelpozo@osakidetza.eus; 10 Pathology Department, Cruces University Hospital, 48903 Barakaldo, Spain; 11 IKERBASQUE, Basque Foundation for Science, 48009 Bilbao, Spain; 12 Radcliffe Department of Medicine, University of Oxford, Oxford OX3 9DU, UK

**Keywords:** renal cancer, sunitinib, resistance, miRNA, transcriptome, pathway analysis

## Abstract

Sunitinib has greatly improved the survival of clear cell renal cell carcinoma (ccRCC) patients in recent years. However, 20–30% of treated patients do not respond. To identify miRNAs and genes associated with a response, comparisons were made between biopsies from responder and non-responder ccRCC patients. Using integrated transcriptomic analyses, we identified 37 miRNAs and 60 respective target genes, which were significantly associated with the NF-kappa B, PI3K-Akt and MAPK pathways. We validated expression of the miRNAs (*miR-223*, *miR-155*, *miR-200b*, *miR-130b*) and target genes (*FLT1*, *PRDM1* and *SAV1*) in 35 ccRCC patients. High levels of *miR-223* and low levels of *FLT1*, *SAV1* and *PRDM1* were associated with worse overall survival (OS), and combined *miR-223* + *SAV1* levels distinguished responders from non-responders (AUC = 0.92). Using immunohistochemical staining of 170 ccRCC patients, VEGFR1 (*FLT1*) expression was associated with treatment response, histological grade and RECIST (Response Evaluation Criteria in Solid Tumors) score, whereas SAV1 and BLIMP1 (*PRDM1*) were associated with metachronous metastatic disease. Using in situ hybridisation (ISH) to detect *miR-155* we observed higher tumoural cell expression in non-responders, and non-tumoural cell expression with increased histological grade. In summary, our preliminary analysis using integrated miRNA-target gene analyses identified several novel biomarkers in ccRCC patients that surely warrant further investigation.

## 1. Introduction

Renal tumours are amongst the most common neoplasms in the Western world, accounting for about 2–3% of all adult cancers, and cases have been increasing during the last 10 years [1,2,3,4]. Clear cell renal cell carcinoma (ccRCC), which represents 70–80% of renal carcinomas, is an aggressive tumour often associated with a poor prognostic outcome as nearly a third of patients present with locally advanced and/or metastatic disease [5,6]. Consequently, there has been great interest in targeted therapies for ccRCC, including therapeutics targeting the vascular endothelial growth factor (VEGF) [7], mammalian target of rapamycin (mTOR) pathways, and more recently, the PD-1/PD-L1 axis (e.g., nivolumab) [8,9,10]. Sunitinib (Sutent^®^), a small molecule inhibitor of multiple receptor tyrosine kinases (RTKs), including VEGF receptors (VEFGR), platelet-derived growth factor receptors (PDGFR), fms-related tyrosine kinase 3 (FLT3), and stem cell growth factor receptors KIT and RET [11,12], has greatly improved the outcome for metastatic ccRCC patients. Sunitinib continues to be a first-line treatment for many patients due to its more tolerable adverse/toxicity profile than other drugs [13,14,15]. The median survival of ccRCC patients treated with sunitinib, however, remains poor (8 to 30 months) [16,17], as nearly a third of patients do not initially respond to treatment, and those that do acquire resistance after ~12 months [18]. Therefore, there is a clear need to better understand the molecular mechanisms of resistance to sunitinib treatment in ccRCC and to target these mechanisms accordingly. 

**Figure 1:**
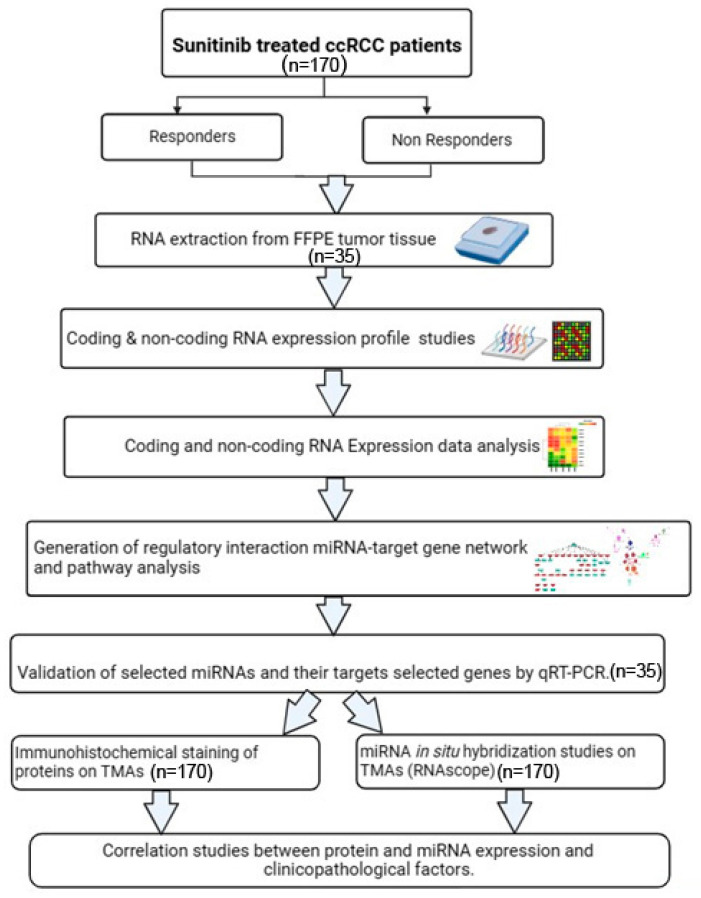
Schematic diagram of the workflow used in this study.

Several mechanisms have been shown to be involved in resistance to sunitinib, including up-regulation of proangiogenic pathways, alterations to the tumour microenvironment (TME), the endoplasmic reticulum stress response, single nucleotide polymorphisms (e.g., *ABCB1* and *ABCG2* genes), changes in the methylation status of *PON1*, as well as the involvement of non-coding RNAs (ncRNAs) such as microRNAs (miRNAs) (reviewed by Jin et al. [13]). The potential role of miRNAs in sunitinib resistance in particular have generated great interest; however, to date, very few of these studies have considered using an integrated omic approach to better understand the functional role of aberrantly expressed miRNAs on target genes and their associated pathways [19,20,21,22,23]. Therefore, we used microarray analysis to identify differentially expressed miRNAs and genes and used an integrated omic network analysis to identify miRNAs and their respective target genes and associated pathways (as outlined in Figure 1). 

## 2. Results

### 2.1. Patient Selection

After a re-review of clinical notes, we identified 174 ccRCC patients that had undergone treatment with sunitinib that attended either University Hospital Donostia ((HUD) San Sebastián, Spain) or University Hospital Cruces ((HUC), Bilbao, Spain). These cases were classified as either responders (R; *n* = 74) (time to progression (TTP) > 24 months post-treatment) or non-responders (NR; *n* = 41) (TTP < 4 months post-treatment) as previously described [24]. These were defined as the NR/R cohort. Cases that had an intermediate response (i.e., TTP 4–24 months; *n* = 59) were not considered in the analysis of response but were used for the correlation analyses for factors other than the sunitinib response. Individual patients’ data, including ISUP (International Society of Urological Pathology) histological grade and RECIST (Response Evaluation Criteria in Solid Tumors) scores from the whole cohort (174 cases) can be found in Supplementary Table S1. Tumour material from 170 of these cases (four had no biopsy material) was used to create a multiple tissue array (TMA). 

The median age of the patients in the NR/R cohort was 59 (58 for males and 60 for females; R = 59 years old and NR = 60 years old) and the median follow-up time was 47 months (Table 1). From this cohort we selected 35 cases, 20 R patients (3 female, 17 male) and 15 NR patients (4 female, 11 male), for which there was sufficient clinical material and full clinical data (including follow-up) available for the molecular analyses. Kaplan–Meier analysis confirmed that the NR patients within this cohort had inferior overall survival (OS) when compared with R patients (*p* value < 0.0001; Supplementary Figure S1) with an OS of 28 months for NR patients compared to 134 months for R patients. 

### 2.2. Non-Coding RNA and Gene Expression in Sunitinib Response 

**Figure 2:**
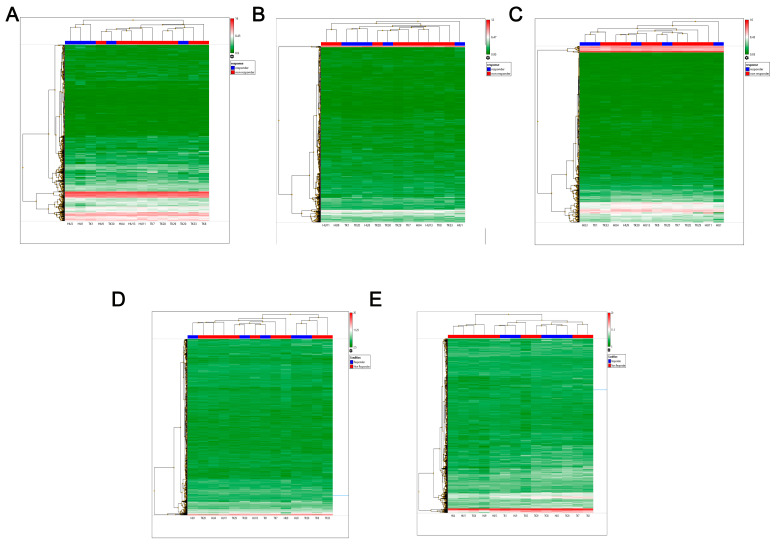
Heatmap of unsupervised cluster analyses depicting expression of (**A**) mature miRNAs, (**B**) pre-miRNAs, (**C**) snoRNAs and scaRNAs, (**D**) lncRNA and (**E**) coding genes in ccRCC cases. The dendrogram at the side shows the distribution of the RNAs, and at the top the relationship between patient samples (blue responder and red non-responder) is shown.

We extracted RNA from the 35 cases for which there was sufficient tumour material; however, only 14 (R = 5, NR = 9) of these cases had sufficiently good quality RNA (i.e., RIN value > 8) in order to carry out microarray analyses. A further 15 cases were identified and extracted but again had only low-quality RNA. We believe this is likely caused by excess time elapsed between the nephrectomy and placing the tissue in formalin as has previously been reported [25]. Unsupervised cluster analysis of the expression of the mature miRNA (Figure 2A), pre-miRNA (Figure 2B), small nucleolar RNAs (snoRNAs) (Figure 2C) and long non-coding RNAs (lncRNAs) (Figure 2D), as well as that of the coding genes (Figure 2E), largely clustered the NR cases distinctly from the R cases.

Using ANOVA analysis, we identified 220 differentially expressed miRNAs (DEmiRNAs) between NR and R cases, of which 141 were upregulated and 79 downregulated in NR patients (Table S2). Additionally, we identified 52 differentially expressed pre-miRNAs, 42 of which were upregulated and 10 were downregulated (Table S3). Twenty-four (46%) of the 52 pre-miRNAs were also dysregulated as mature miRNAs, including up-regulation of multiple members of the *miR-200* family (i.e., *miR-200a*, *miR-200b*, *miR-200c* and *miR-429*), the *miR-17~92* cluster (i.e., *miR-17*, *miR-18a* and *miR-19a*), and twenty members of the chromosome 14 cluster. There were 511 differentially expressed lncRNAs between the NR and R cases, 189 of which were upregulated and 322 downregulated (Table S4), and 49 differentially expressed snoRNAs, only four of which were downregulated (Table S5). From the gene expression analysis, we identified 1026 differentially expressed encoding genes (DEgenes), of which 234 were upregulated and 792 downregulated in the NR cases (Table S6). 

### 2.3. Interaction Network Analysis 

**Figure 3:**
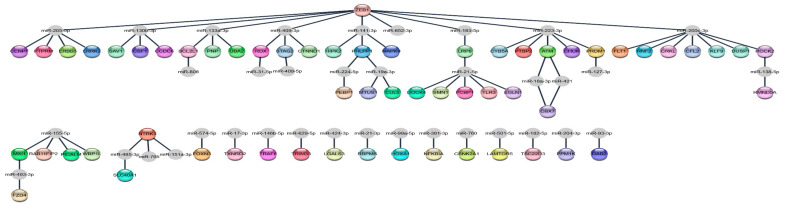
String visualisation network of miRNA–target gene interactions associated with sunitinib resistance in ccRCC patients.

In order to identify genes regulated by miRNAs associated with the response to sunitinib, we created an interaction network by mapping the DEmiRNAs and DEgenes that were reciprocally expressed (i.e., down-regulated miRNAs and up-regulated genes and vice versa) to a database of experimentally validated miRNA–gene target interactions (*n* = 10,754). In this way, we identified 60 genes (all down-regulated) and 37 miRNAs (all up-regulated) (Figure 3, Table 2).

**Figure 4:**
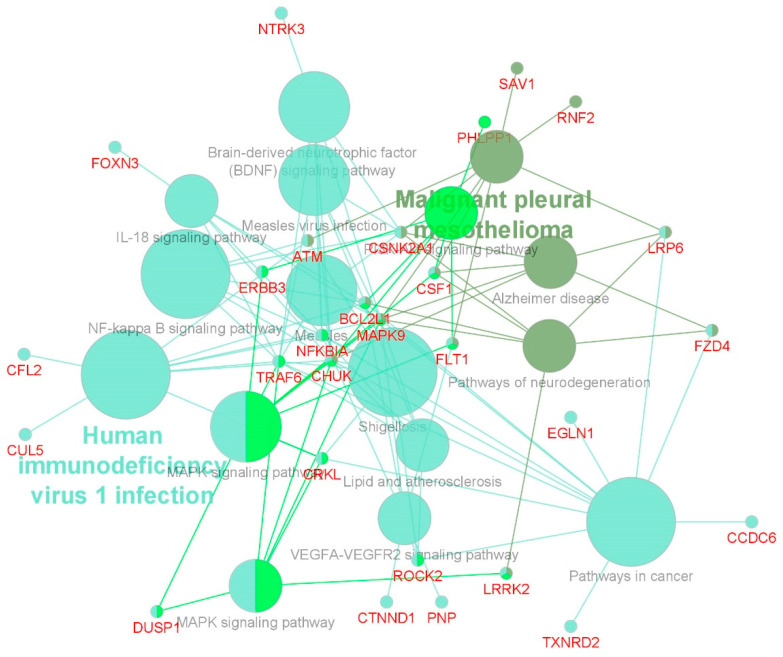
Gene ontology and pathway mapping of miRNA targeted genes. Terms are functionally grouped based on shared genes (kappa score) and are shown in different colours. The node size represents the degree of significance.

In order to gain more insight into the biological function of the miRNA targeted genes we carried out functional pathway enrichment analysis using the Cluego and Cluepedia algorithms [26,27] (Figure 4; Supplementary Figure S2; Table 3). From this analysis we found a significant enrichment for NF-kappa B, IL-18, VEGFR, PI3K-Akt and MAPK signalling pathways, amongst others (Table 3).

### 2.4. Validation of Identified miRNAs and Genes

**Figure 5:**
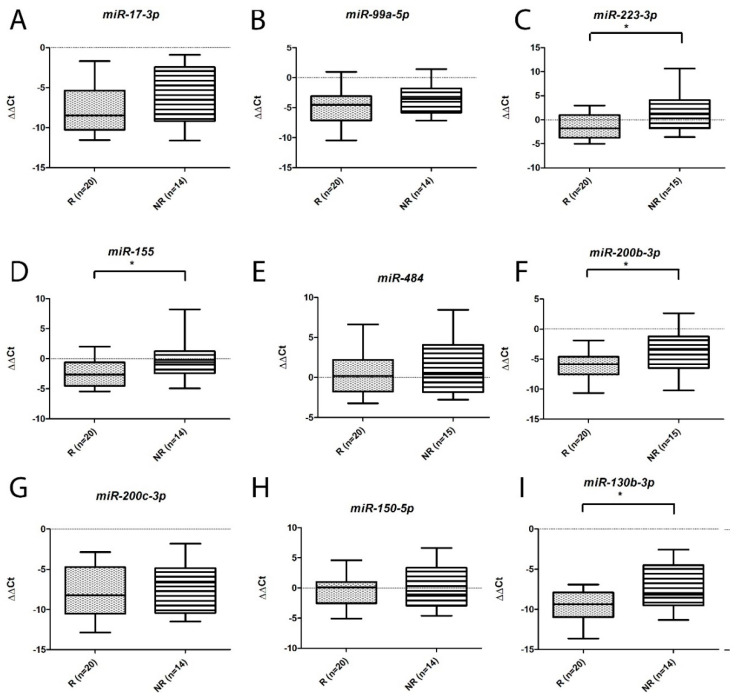
Box and whisker plots of levels of differentially expressed miRNAs measured by qRT-PCR in NR and R ccRCC cases. (**A**) *miR-17-3p*; (**B**) *miR-99a-5p*; (**C**) *miR-223-3p*; (**D**) *miR-155*; (**E**) *miR-484*; (**F**) *miR-200b-3p;* (**G**) *miR-200c-3p*; (**H**) *miR-150-5p*; (**I**) *miR-130b-3p*. Significant differences (*p* < 0.05) are denoted by asterisks (*).

**Figure 6:**
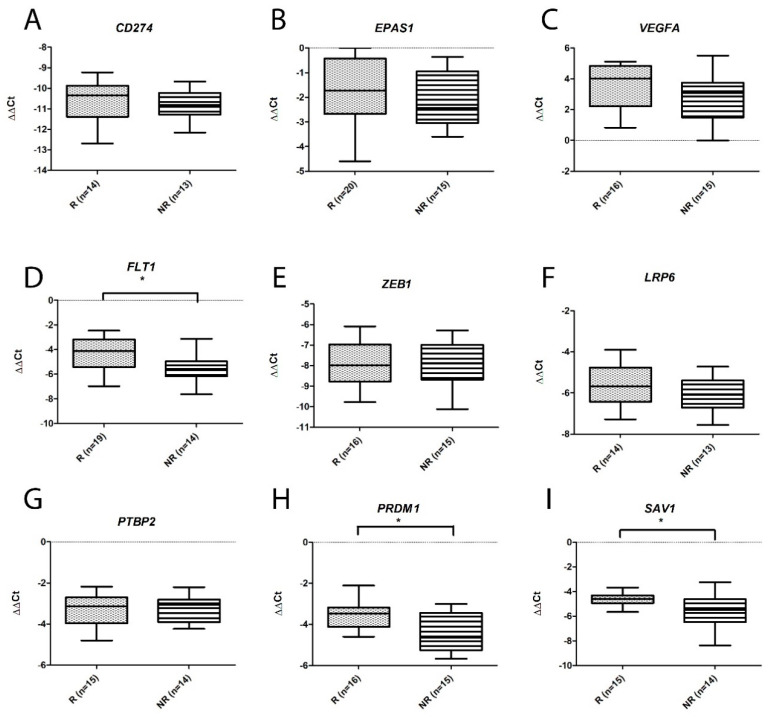
Box and whisker plots of levels of differentially expressed genes measured by qRT-PCR in NR and R ccRCC cases. (**A**) *CD274*; (**B**) *EPAS1*; (**C**) *VEGFA*; (**D**) *FLT1*; (**E**) *ZEB1*; (**F**) *LRP6;* (**G**) *PTBP2*; (**H**) *PRDM1*; (**I**) *SAV1*. Significant differences (*p* < 0.05) are denoted by asterisks (*).

On the basis of the above findings and previously published associations with renal cancer, nine miRNAs and nine genes were selected for further validation. As can be seen from Figure 5, although the levels of all of the tested miRNAs were higher in NR patients than R patients (consistent with the microarray results), only the levels of *miR-223*, *miR-155*, *miR-130b-3p* and *miR-200b-3p* were significantly so. Also consistent with the microarray results we observed that all of the genes were down-regulated (Figure 6), although only the downregulation of *FLT1*, *PRDM1*, and *SAV1* was significant in this cohort. 

### 2.5. Association of the Expression of Identified Potential Molecular Markers and Clinical Outcomes

**Figure 7:**
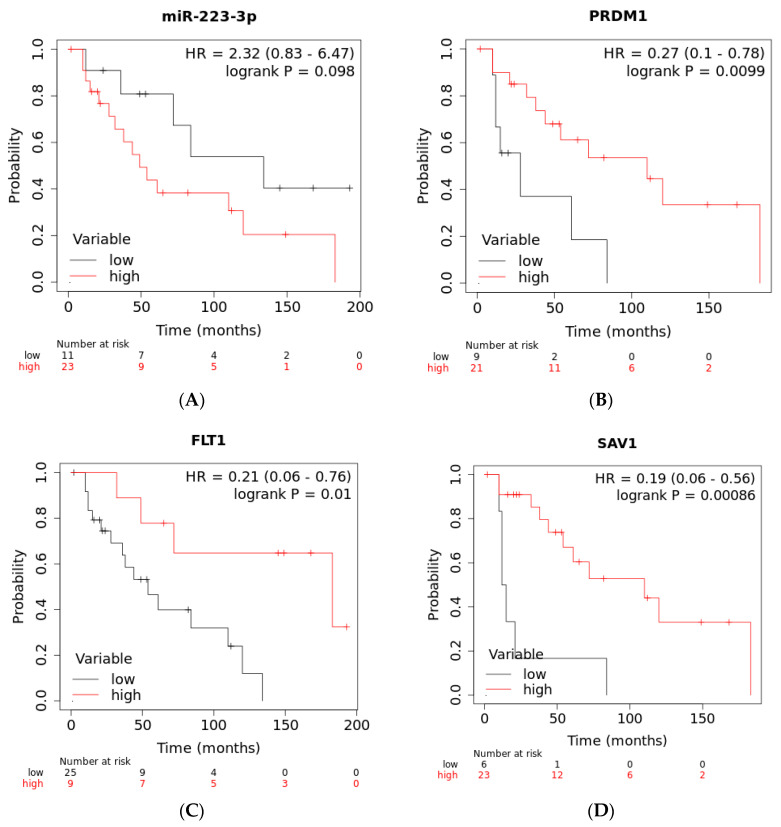
Kaplan–Meier survival curves in univariate analysis of expression levels of (**A**) *miR-223-3p*, (**B**) *PRDM1*, (**C**) *FLT1* and (**D**) *SAV1* as a function of overall survival (OS) in months.

As the response to sunitinib treatment was clearly associated with overall survival (OS) in this cohort of ccRCC patients (Supplementary Figure S1), we investigated whether levels of the validated miRNAs and genes were also associated with survival. We observed that high levels of *miR-223* (although it did not reach statistical significance) and low levels of *PRDM1*, *SAV1* and *FLT1* were indeed associated with shorter OS of 49, 28, 15 and 54 months, respectively, compared to medians of 134, 110, 110 and 183 months, respectively, for patients with low expression of *miR-223* and high expression of` *PRDM1*, *SAV1* and *FLT1* (Figure 7). The expression levels of *miR-155*, *miR-130b* and *miR-200b* were not significantly associated with OS in this cohort.

To evaluate the ability of the identified genes and miRNAs to differentiate between ccRCC cases that responded to sunitinib from those that did not, we carried out ROC analysis. Levels of *miR-223-3p*, *PRDM1*, *FLT1* and *SAV1* alone had area under the curve (AUC) values ≥ 0.7 (0.73, 0.77, 0.83 and 0.83, respectively), as did a combination of all miRNAs, combinations of three miRNAs (*miR-223 + miR-155 + miR-200b* and *miR-223 + miR-155 + miR-130*) and of two miRNAs (*miR-223 + miR-155* and *miR-223 + miR-200b*) (Supplementary Table S7. A combination of all three genes (i.e., *PRDM1 + FLT1 + SAV1*) gave an AUC value of 0.9 and other gene combinations had values >0.8. The highest AUC values resulted from a combination of *SAV1* and *miR-223* (AUC = 0.92).

### 2.6. Protein Expression of miRNA Target Genes Is Associated with Sunitinib Response

In order to explore further the correlation between sunitinib response and protein expression of the validated genes, we carried out immunohistochemical staining of VEGFR1 (*FLT1*), SAV1 and BLIMP1 (*PRDM1*) on 170 ccRCC cases uniformly treated with sunitinib. We also carried out PD-L1 staining on these cases as we previously reported the involvement of this molecule in an in vitro model of sunitinib resistance [28]. Of these cases, 56% (84/151) were positive for SAV1 expression, 39% (55/142) for VEGFR1 expression, 13% (20/152) for BLIMP1 expression and 58% (96/164) for PD-L1 expression. Correlation analysis of the protein expression with clinical parameters (i.e., M (metastasis), histological ISUP grade, RECIST score and response) found significant correlations between VEGFR1 expression and decreasing histological ISUP grade (*p* = 0.003), RECIST score (*p* = 0.021) and sunitinib responsiveness (*p* = 0.020) (Table 4). There were also correlations between SAV1 and BLIMP1 expression and the presence of metachronous metastatic disease (*p* = 0.013 and 0.006, respectively), and BLIMP1 expression was found to be associated with male patients (*p* = 0.048) (Table 4). We also carried out correlation analysis with tumour stage (pT), presence of nodes (N), and the ECOG (Eastern Cooperative Oncology Group) performance status scale and IMDC (International Metastatic Renal-Cell Carcinoma Database Consortium) indicators but found no significant correlation with any of the biomarkers tested

### 2.7. In Situ Expression of miR-155 in Tumour Cells but Not Non-Tumour Cells Is Associated with Sunitinib Response

**Figure 8:**
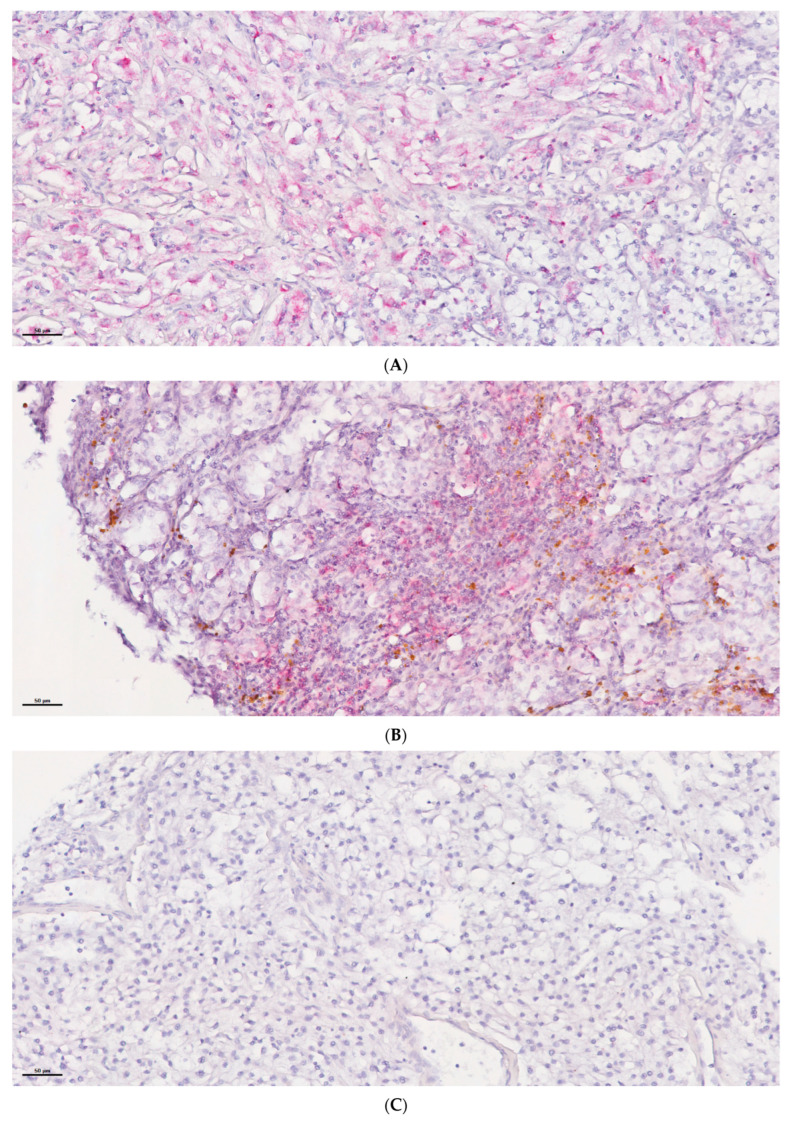
Examples of *miR-155* expression detection by ISH in ccRCC cases demonstrating (**A**) positive expression in tumour cells, (**B**) positive expression in non-tumour cells and (**C**) negative expression.

As the levels of VEGFR-1 were associated with sunitinib response in this cohort, we explored the expression of the targeting miRNA, *miR-155*, using miRNA in situ hybridisation (miRNA ISH) (Figure 8). The expression levels were then scored according to whether the expression was associated with tumour cells or non-tumour cells. Tumour cells in 8.8% of the ccRCC cases (14/159 countable cases) were positive for *miR-155* expression whereas non-tumoural cells were positive in 74.8% of cases (110/147 countable cases). Correlation analysis revealed that cases with *miR-155* tumour cell expression were associated with non-responder cases (χ^2^ = 10.79; *p =* 0.029) (Table 4). In contrast, there was no correlation between *miR-155* expression in non-tumoural cells and response, but there was a correlation between expression and increased histological ISUP grade (χ^2^ = 45.52; *p =* 0.007).

## 3. Discussion

Sunitinib remains the first line treatment of choice for many metastatic ccRCC patients due to its low toxicity profile and durability of response [29,30]. However, despite the great improvements made to the overall survival of many treated patients, a significant proportion do not respond to treatment and of those that do, many will develop resistance within 12 months and undergo disease progression. Therefore, there has been a great deal of interest in understanding the molecular mechanisms of sunitinib resistance. Previous research studies, including ours, have shown the involvement of miRNAs in sunitinib resistance [19,20,28,31,32,33,34,35,36]. However, the majority of the studies that investigated patient responsiveness to treatment are limited to the identification of differentially expressed miRNAs, meaning that many of the identified miRNAs could result from indirect associations without having a functional role in response. Indeed, although the unsupervised cluster analysis of both differentially expressed ncRNAs (miRNAs, pre-miRNAs, snoRNAs and lncRNAs) and genes were generally distinct between patients that responded to treatment from those that did not (Figure 2), those cases that did not cluster distinctly in the different classes of RNA were inconsistent, suggesting that this approach was not sufficient to identify ncRNAs and genes truly associated with sunitinib response. To overcome this limitation, a more focused approach is necessary. 

We focused on only those miRNAs associated with response that had target genes reciprocally differentially expressed. This integrated omic approach resulted in 17% of miRNAs (37/220) and 6% (60/1026) of genes being selected. Using pathway analysis to examine potential common functional roles for the genes, we established that amongst the most enriched pathways was NF-kappa B signalling (*p* = 1.9 × 10^−4^; 6/60 genes), consistent with the findings of Aimudula et al. [37] and Makhov et al. [38]. In addition, MAPK, IL-18 and PI3K-Akt pathways were significantly enriched as well as VEGFA-VEGFR2 signalling pathways. All of these pathways have previously been associated with sunitinib resistance, demonstrating the robustness of this integrated omic approach [39,40,41,42,43]. It should be noted, however, that the miRNA:target gene interactions identified in this study are based upon published binding studies, and nearly all of these studies were conducted under physiological conditions and therefore may not accurately reflect the complexity that occurs in cancer patients.

Expression levels of *miR-223*, *miR-155*, *miR-130b* and *miR*-200b were found to be upregulated in cases non-responsive to sunitinib treatment when compared to responsive patients. Both *miR-130b* and *miR-200b* have previously been identified by our group as being differentially expressed in a previous in vitro model of sunitinib resistance [28]. *MiR-223* was identified by Butz et al. as being differentially expressed in a xenotransplant model of sunitinib resistance [44]. Merhautova et al. also observed a decrease in *miR-155* levels in sunitinib responsive patients [22] and *miR-130b* was previously related to sunitinib resistance in renal cancer. Levels of this miRNA were found to be higher in sunitinib resistant cells when compared with parental ones [45]. In agreement with our results, the work of Zhou et al., using the TCGA database, showed that high expression levels of *miR-130b* in renal tumour samples were related to worse survival [46]. We also demonstrated that down-regulation of *FLT1*, *PRDM1* and *SAV1* were significantly associated with non-responsive ccRCC patients. Expression of *FLT1* has been linked to sunitinib response in an in vitro model of glioblastoma [47]. As far as we are aware, the expression of *PRDM1* and *SAV1* has not been identified before as being involved in sunitinib response.

There was an association between high expression of *miR-223* and low expression of *PRDM1*, *SAV1* and *FLT1* with a poor prognostic outcome in this cohort of ccRCC cases, as shown in the survival analysis. Kowalik et al. also demonstrated that high expression levels of *miR-223* were related to higher tumour stages and grades [48], and other studies have shown that ccRCC patients with high expression of *miR-223* had a shorter OS [49,50]. It has previously been shown that *FLT1* expression was higher in ccRCC cases that responded well to sunitinib [51,52] and was associated with the prognostic outcome [52].

Individual levels of *miR-223* and the genes *PRDM1*, *SAV1* and *FLT1* all had good biomarker ability to discriminate ccRCC patients that were responders from non-responders (AUC > 0.7). However, the best results were obtained with a combination of *miR-223* and *SAV1* (AUC = 0.92), although *SAV1* alone (AUC = 0.83) or in combination with any of the miRNAs (i.e., *miR-155*, *miR-130b* or *miR-200b*) gave high scores (AUC ≥ 0.89). Although these results clearly need to be repeated in larger cohorts, the AUC values obtained are very promising and much better than those obtained with other published biomarkers of sunitinib response in ccRCC [24]. The inclusion of an miRNA in particular is promising as a useful biomarker, especially if it could be detected in blood or urine as we have carried out previously [53,54]. This is an area we are actively investigating.

To further explore the identified target genes, we carried out immunohistochemical staining on 170 cases of sunitinib treated ccRCC cases to investigate protein expression of SAV1, VEGFR1 (*FLT1* gene) and BLIMP1 (*PRDM1* gene), as well as PD-L1, which we had previously identified in our cell model of sunitinib resistance but was not identified in the current analyses [28]. We observed a positive correlation between VEGFR1 expression and those patients that responded to sunitinib as well as a negative correlation with increasing RECIST score and histological ISUP grade. These findings concur with previous studies that demonstrated an association between VEGFR1 expression and better patient outcomes at both the protein [55] and mRNA levels [52], and is consistent with the RECIST score, which is a measure of treatment response [56]. A negative correlation between VEGFR1 expression and (Fuhrman) histological grade has also previously been demonstrated by Lkhagvadorj et al. [57]. We observed that the expression of both BLIMP1 and SAV1 were associated with patients that had metachronous rather than synchronous metastases. Patients with synchronous metastases have been demonstrated to have poorer OS than those with metachronous metastases in a series of 48 ccRCC patients [58], thirteen of whom were treated with sunitinib. Consistent with those findings, we found a significant correlation between metachronous metastases and sunitinib response in our cohort (*p* < 0.001).

SAV1 expression has been linked to progression in gastric cancer [59], hepatic carcinoma [60] and pancreatic cancer [61]. Our results were consistent with others who found an association between SAV1 expression and high grade ccRCC [62,63]. Interestingly, *miR-130b*, which we demonstrated was overexpressed in sunitinib non-responders, targets SAV1 [64].

BLIMP1 has been identified as a key driver of metastasis in pancreatic cancer [65] and lung cancer [66], although we are not aware of a previously characterised role of this molecule in renal cancer tumour cells. It has been reported, however, that BLIMP1 is expressed in resident CD8^+^ T cells of ccRCC cases [67], which are more frequently found in metastatic ccRCC and are associated with a poor prognostic outcome [68]. Intriguingly, expression of BLIMP1 was also associated with male gender in our cohort. The reason for this correlation remains unclear although it has been described that BLIMP1 expression has a gender bias in dendritic cells (DCs) [69] and that DCs are highly enriched in the ccRCC microenvironment [70]. We found no significant correlations between PD-L1 expression and clinical parameters in this study.

We identified *miR-155* as being down-regulated in sunitinib responsive patients and although we found that its expression was associated with the prognostic outcome in this cohort, it was not significant. The role of *miR-155* expression in renal cancer is well documented [71,72] and is linked with survival in sunitinib treated patients [22,73]. As VEGFR1 (*FLT1*) expression has been demonstrated to be regulated by *miR-155* [74], we investigated the expression of this miRNA further by carrying out miRNA ISH on the 170 cases contained on the TMA. miRNA ISH has been used previously to detect *miR-21* and *miR-382* in rat kidney tissue [75,76], and *miR-126*, *miR-222* and *miR-221* in ccRCC (*n* = 37), papillary RCC (*n* = 28), chromophobe RCC (*n* = 20) and oncocytoma (*n* = 13) cases [77]. MiRNA ISH has been used to detect *miR-155* expression in lung cancer [78], cutaneous T cell lymphoma [79] and pancreatic cancer [80], but as far as we are aware this is the first description of miRNA ISH being used to examine *miR-155* expression in kidney cancer. We found a correlation between the expression of *miR-155* in tumour cells, but not in non-tumour cells, with sunitinib responsiveness. 

The up-regulation of *miR-155* has been shown to increase proliferation and invasion potential of ccRCC tumour cells in vitro and was found to be associated with clinical aggressiveness through the targeting of E2F2 [81] and JADE-1 [82]. A potential role for *miR-155* targeting *FLT1* in sunitinib resistance in ccRCC has not previously been postulated and is an area that surely warrants further investigation. The observation that *miR-155* expression was associated with histological ISUP grade in non-tumour cells but not in tumour cells is intriguing as this parameter is a measure of the neoplastic cell morphological differentiation state and does not take into account the tumour microenvironment (TME). Our results suggest that the expression of TME-derived *miR-155*, rather than tumour-derived *miR-155*, confers these morphological changes. It has been demonstrated that exosome delivered *miR-155* derived from tumour associated macrophages (TAMs) changes the phenotype of ccRCC tumour cells in vitro and in vivo [83].

**Figure 9:**
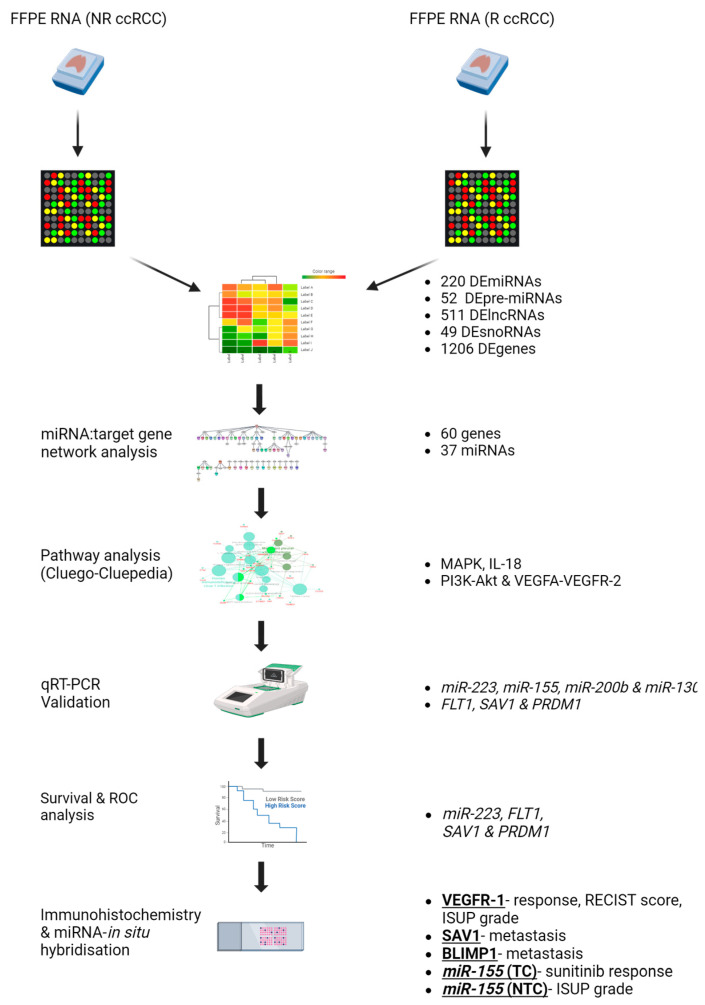
Schematic summary of main findings in this study.

In summary (Figure 9), using an integrated omic approach for the identification of miRNAs and their respective target genes associated with sunitinib resistance in ccRCC patients, we have provided further insight into resistance mechanisms and identified potential targets for future studies.

## 4. Materials and Methods

### 4.1. Patient Selection and Patient Material

The 174 ccRCC patients were retrospectively selected from among patients uniformly treated with sunitinib as frontline therapy that attended either Hospital Donostia (*n* = 67) or Hospital Cruces (*n* = 107). All patients were treated with sunitinib as frontline therapy, and biopsy samples (nephrectomy FFPE blocks) were taken at the time of diagnosis prior to treatment. Cases were classified as responders when the time to progression (TTP) was greater than 24 months or as non-responders when the TTP was less than 4 months as previously described [24]. Both response and progression criteria for ccRCC cases were assessed by clinicians in accordance with the RECIST guidelines [56].

The corresponding FFPE blocks from 170 ccRCC cases (4 were missing or had no biopsy material remaining) were retrospectively retrieved from the pathology departments of the respective hospital and the cases were re-reviewed by a uropathologist who selected an area of high tumor load (>70%) in order to construct multiple tissue microarrays (TMA) for immunohistochemical staining and in situ hybridisation (ISH). Written informed consent was obtained from the patients for the inclusion of their samples in this study and the samples were collected in accordance with the Declaration of Helsinki and approved by local ethics committees (CEIm-Euskadi approval number PI2015059X).

### 4.2. RNA Extraction and Microarray Analysis

Total RNA used for molecular analysis (i.e., microarray and qRT-PCR) was extracted from whole section FFPE biopsy material from 35 cases using the RecoverAll kit in accordance with the manufacturer’s instructions (Thermo Fisher Scientific Inc., Waltham, MA, USA).

One µg and 200 ng of purified RNA was labelled and hybridised to Affymetrix Genechip miRNA v.4.0 microarrays and Clariom D human microarrays, respectively, in accordance with the manufacturer’s instructions (Thermo Fisher). The resultant intensity data (i.e., cel files) from both microarray platforms were imported and analysed using the Transcriptome Analysis Console (TAC) software version 4.0.2 (Thermo Fisher). Differentially expressed miRNAs and genes were identified on the basis of >1.5-fold expression changes and *p* < 0.05 between NR and R patients (Supplementary Table S3). All microarray data were Minimum Information About a Microarray Experiment (MIAME) compliant and the raw data have been Gene Expression Omnibus (GEO) database (pending accession number).

### 4.3. Interaction Network Analysis

Lists of differentially expressed genes (DEgenes) and miRNAs (DEmiRNAs) were imported into Cytoscape (v 3.9.1) in order to construct miRNA–target gene interaction networks as previously described [84]. In brief, we used the miRTarBase [85] dataset filtered to include only experimentally validated miRNA–gene interactions (10,754 interactions) to create a network based on differentially expressed genes that were inversely correlated with differentially expressed miRNAs (i.e., genes up-regulated and miRNAs down-regulated and vice versa).

Enriched gene ontology biological pathways were identified and visualised using Cytoscape plug-in ClueGO and Cluepedia v.5.9 [26,27]. Functional enrichment was performed using ontologies: GO, Biological Process y Molecular Function, KEGG and Reactome.

### 4.4. miRNA and Gene Expression (qRT-PCR)

For miRNA expression measurement, 500 ng of RNA was reverse transcribed using Taqman Megaplex™ miRNA pool A according to the manufacturers’ instructions (Thermo Fisher). The resultant cDNA was amplified using Megaplex™ PreAmp Primers pool A and Taqman PreAmp Master Mix following the manufacturers’ instructions (Thermo Fisher). The resulting cDNA was diluted 1:40 before carrying out qPCR using individual Taqman probes in triplicate on a LightCycler^®^ 96 System machine (Roche, Basel, Switzerland). *RNU48* levels were used as control. 

For gene expression analysis, 500 ng of RNA was reverse transcribed using SuperScript IV VILO Master Mix system (Thermo Fisher) and the cDNA amplified using Taqman PreAmp Master Mix (Thermo Fisher) and a pooled set of Taqman Assays prepared by combining the individual assays for the genes of interest in a final concentration of 0.2X. Following a 1:5 dilution, the amplified cDNA was used for qRT-PCR using individual Taqman probes in triplicate in a Bio-Rad Maestro CFX system. *B2M* was used as a control gene. Samples with Ct values > 35 were removed from the analysis as being unreliable. The mean Ct value of each triplicate was used for analysis with the ΔΔCt method. Expression levels were compared using the Mann-Whitney U-test (GraphPad Prism v.5.0, La Jolla, CA, USA).

### 4.5. Statistical Analyses

Binary regression logistic models correlating the sunitinib response and the expression of the miRNAs/genes were carried out followed by ROC analysis as implemented using MedCalc^®^ Statistical Software version 20.216 (MedCalc Software Ltd., Ostend, Belgium). The output was graphically plotted using ROCplotter (https://rocplot.org/) [86].

Survival analyses were performed using the Kaplan–Meier method with a long-rank test implemented in KMplot [87,88]. OS was defined as the time between the first diagnosis and patient death due to disease. Patients who were alive at the time of the study or lost to follow up were treated as censored events.

Correlation analysis between categorical variables for immunohistochemical and in situ hybridisation were carried out using Chi-square (χ^2^) analysis implemented in SPSS^®^ 29.0 software (IBM, New York, NY, USA).

### 4.6. Immunohistochemical Staining and miRNA In Situ Hybridisation

Immunohistochemistry was performed according to standard protocols using an automated immunostainer (AutoStainer Link 48 Dako, Glostrup, Denmark). The EnVision Flex visualization system was used as recommended by the manufacturer. In brief, after deparaffination and rehydration of the slides, antigen retrieval was performed using Dako PT link pre-treatment and citrate (pH 9) retrieval buffer before incubating the slides with antibodies against VEGFR1 (Abcam (Cambridge, UK) [Y103] ab32152, 1:100 dilution), SAV1 (Merck (Darmstadt, Germany); Cat. No. MABS1708; 1:50 dilution) and BLIMP-1 (Merck, clone ROS195G, Cat. No. MABE1814,1:50). HRP-conjugated secondary antibodies were used at a 1:2000 dilution and staining was visualized using a DAB kit (Abcam) and then the sections were counterstained with haematoxylin (Panreac Quimica, Barcelona, Spain) according to the manufacturers’ instructions. PD-L1 staining was carried out with the SP142 antibody on a Ventana machine according to the standard manufacturers’ procedure (Roche Diagnostics).

Detection of miRNA by in situ hybridisation (ISH) was carried out using the miRNAscope™ HD (RED) Assay 324,510 (Advanced Cell Diagnostics (ACDBio), Newark, NJ, USA) and miRNAscope™ Probe-SR-hsa-miR-155-5p-S1 MIMAT0000646 (727991-S1) according to the manufacturers’ instructions. Scoring of miR-155 expression was carried out by an expert uropathologist who scored the expression as absent or present in tumour cells or non-tumour cells.

## Figures and Tables

**Table 1:** Summary of clinical characteristics of ccRCC cases used in this study.

Patients’ Characteristics	Treatment Response		
	Responders	Non-Responders	Not Known/Unclassified
Number	74	41	59
Sex			
Female	20	13	13
Male	54	28	31 (15 NK)
Median age: 59	59	60	58
Median female age: 60	60.5	62	63
Median male age: 58	58.5	57	51
Median follow-up (months)	47	13	-

**Table 2:** List of DEmiRNAs and their respective predicted DEgene targets identified by network analysis.

miRNA	Target Gene(s)
*hsa-miR-138-5p*	*RMND5A*, *ROCK2*
*hsa-miR-141-3p*	*ZEB1*, *MAPK9*, *HIPK2*, *PHLPP1*
*hsa-miR-146-5p*	*TRAF6*
*hsa-miR-151-3p*	*NTRK3*
*hsa-miR-155-5p*	*RAB11FIP2*, *WBP1L*, *MXI1*, *PICALM*, *FLT1*
*hsa-miR-17-3p*	*TXRND2*
*hsa-miR-182-5p*	*TSC22D3*, *LRP6*, *ZEB1*
*hsa-miR-18a-3p*	*ATM*, *CBX7*
*hsa-miR-183-5p*	*LRP6*
*hsa-miR-19a-3p*	*CUL5*, *MTUS1*, *PHLPP1*
*hsa-miR-200b-3p*	*ZEB1*, *RNF2*, *FLT1*
*hsa-miR-200c-3p*	*ZEB1*, *FLT1*, *RNF2*, *CFL2*, *DUSP1*, *KLF9*, *CRKL*, *ROCK2*
*hsa-miR-204-3p*	*PPM1K*
*hsa-miR-205-5p*	*PTPRM*, *LRRK2*, *ERBB3*, *CENPF*, *ZEB1*
*hsa-miR-21-5p*	*RPBMS*
*hsa-miR-21-3p*	*EGLN1*, *LRP6*, *TRL3*, *DOCK4*, *SMN1*, *PCBP1*
*hsa-miR-223-3p*	*PTBP2*, *CYB5A*, *ATM*, *CHUCK*, *PRDM1*, *ZEB1*
*hsa-miR-224-5p*	*PHLPP1*, *PEBP1*
*hsa-miR-31-5p*	*RDX*
*hsa-miR-381-3p*	*NFKB1A*
*hsa-miR-409-3p*	*RDX*, *STAG2*, *ZEB1*, *CTNND1*
*hsa-miR-421*	*ATM*, *CBX7*
*hsa-miR-424-3p*	*LGALS3*
*hsa-miR-485-3p*	*SLC40A1*, *NTRK3*
*hsa-miR-493-3p*	*FZD4*, *MXI1*
*hsa-miR-501-5p*	*LAMTOR5*
*hsa-miR-574-5p*	*FOXN3*
*hsa-miR-608*	*BCL2L1*
*hsa-miR-629-5p*	*TRIM33*
*hsa-miR-652-3p*	*ZEB1*
*hsa-miR-760*	*CSNK2A1*
*hsa-miR-765*	*NTRK3*
*hsa-miR-93-3p*	*DAB2*
*hsa-miR-99a-5p*	*HOXA1*
*hsa-miR-127-3p*	*PRDM1*
*hsa-miR-130b-3p*	*ZEB1*, *SAV1*, *CSF1*, *CCDC6*
*hsa-miR-133a-3p*	*BCL2L1*, *PNP*, *ZEB1*, *UBA2*

**Table 3:** Functional enrichment of the identified DEG results using Cluego, showing the associated genes with the pathways and the percentage of mapped genes from the total number of genes from the term.

Pathway	Corrected *p* Value	% Genes	N Genes	Associated Genes
HIV1 infection	1.6 × 10^−5^	4.25	9	*ATM*, *BCL2L1*, *CFL2*, *CHUK*, *CRKL*, *CUL5*, *MAPK9*, *NFKBIA*, *TRAF6*
Pathways in cancer	1.55 × 10^−4^	2.26	12	*BCL2L1*, *CCDC6*, *CHUK*, *CRKL*, *EGLN1*, *FZD4*, *LRP6*, *MAPK9*, *NFKBIA*, *ROCK2*, *TRAF6*, *TXNRD2*
NF-kappa B signalling pathway	1.9 × 10^−4^	5.77	6	*ATM*, *BCL2L1*, *CHUK*, *CSNK2A1*, *NFKBIA*, *TRAF6*
Shigellosis	4.09 × 10^−4^	3.24	8	*ATM*, *BCL2L1*, *CHUK*, *CRKL*, *MAPK9*, *NFKBIA*, *ROCK2*, *TRAF6*
Measles virus infection	7.56 × 10^−4^	4.35	6	*BCL2L1*, *CHUK*, *CSNK2A1*, *MAPK9*, *NFKBIA*, *TRAF6*
Yersinia infection	7.86 × 10^−4^	4.38	6	*CHUK*, *CRKL*, *MAPK9*, *NFKBIA*, *ROCK2*, *TRAF6*
Brain-derived neurotrophic factor (BDNF) signalling pathway	7.98 × 10^−4^	4.17	6	*CHUK*, *CSNK2A1*, *MAPK9*, *NFKBIA*, *NTRK3*, *TRAF6*
MAPK signalling pathway	9.02 × 10^−4^	2.72	8	*CHUK*, *CRKL*, *CSF1*, *DUSP1*, *ERBB3*, *FLT1*, *MAPK9*, *TRAF6*
Lipid and atherosclerosis	5.58 × 10^−3^	2.79	6	*[BCL2L1*, *CHUK*, *MAPK9*, *NFKBIA*, *ROCK2*, *TRAF6]*
Malignant pleural mesothelioma	9.83 × 10^−3^	1.79	8	*ATM*, *CSF1*, *CSNK2A1*, *FLT1*, *LRP6*, *MAPK9*, *RNF2*, *SAV1*
Alzheimer disease	1.22 × 10^−2^	1.56	6	*CHUK*, *CSF1*, *CSNK2A1*, *FZD4*, *LRP6*, *MAPK9*
IL-18 signalling pathway	1.32 × 10^−2^	2.15	6	*BCL2L1*, *CHUK*, *FOXN3*, *MAPK9*, *NFKBIA*, *TRAF6*
Pathways of neurodegeneration	1.88 × 10^−2^	1.47	7	*BCL2L1*, *CSF1*, *CSNK2A1*, *FZD4*, *LRP6*, *LRRK2*, *MAPK9*
VEGFA-VEGFR2 signalling pathway	2.45 × 10^−2^	1.59	7	*BCL2L1*, *CTNND1*, *FLT1*, *MAPK9*, *NFKBIA*, *PNP*, *ROCK2*
PI3K-Akt signalling pathway	2.52 × 10^−2^	1.69	6	*BCL2L1*, *CHUK*, *CSF1*, *ERBB3*, *FLT1*, *PHLPP1*

**Table 4:** Chi-square (χ^2^) analysis of protein expression and miR-155 expression vs. clinical parameters and gender. NK; not known. Significant values are shaded in grey.

	M (Metastasis)		Histological ISUP Grade		RECIST	Score	Response		Gender	
	χ^2^	*p*-Value	χ^2^	*p*-Value	χ^2^	*p*-Value	χ^2^	*p*-Value	χ^2^	*p*-Value
PD-L1	4.615	0.329	10.863	0.210	9.287	0.319	0.187	0.911	1.912	0.752
VEGFR1	0.110	0.946	16.253	0.003	11.609	0.021	9.536	0.020	0.610	0.737
SAV1	12.711	0.013	4.267	0.832	9.626	0.292	0.055	0.973	2.722	0.605
BLIMP1	14.507	0.006	5.313	0.724	10.742	0.233	0.130	0.937	9.580	0.048
*miR-155* TC	NK	NK	6.878	0.737	NK	NK	10.789	0.029	12.880	0.378
*miR-155* NTC	NK	NK	45.521	0.007	NK	NK	5.519	0.854	26.067	0.350

## Data Availability

Data available in a publicly accessible repository. Gene Expression Omnibus (GEO) database (pending accession number).

## Supplementary Materials

The supporting information can be downloaded at: https://www.mdpi.com/article/10.3390/ijms25136881/s1.
