# Supplementary material for: Identification of miRNAs and Their Target Genes Associated with Sunitinib Resistance in Clear Cell Renal Cell Carcinoma Patients
Source: Int J Mol Sci. 2024 Jun 22;25(13):6881. doi: 10.3390/ijms25136881 (PMC11241516; doi:10.3390/ijms25136881)
Supplement: Supplementary file 1 [file ijms-25-06881-s001.zip › ijms-3043695-supplementary.pdf]

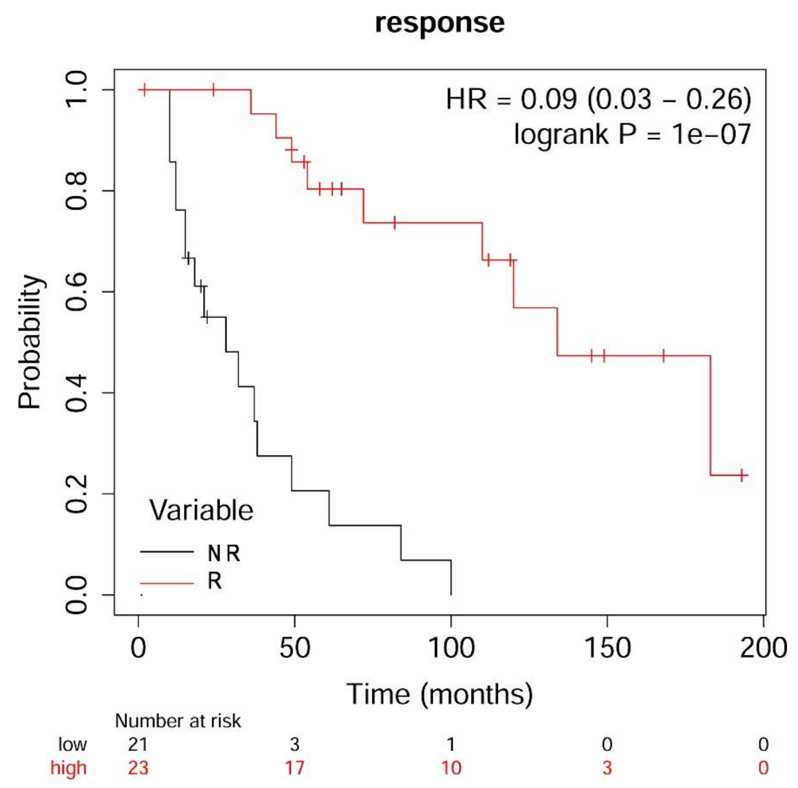

Supplementary Figure S1. Kaplan-Meier analysis of overall survival (OS) of ccRCC patients used in the study as a function of response and non-response to sunitinib treatment.

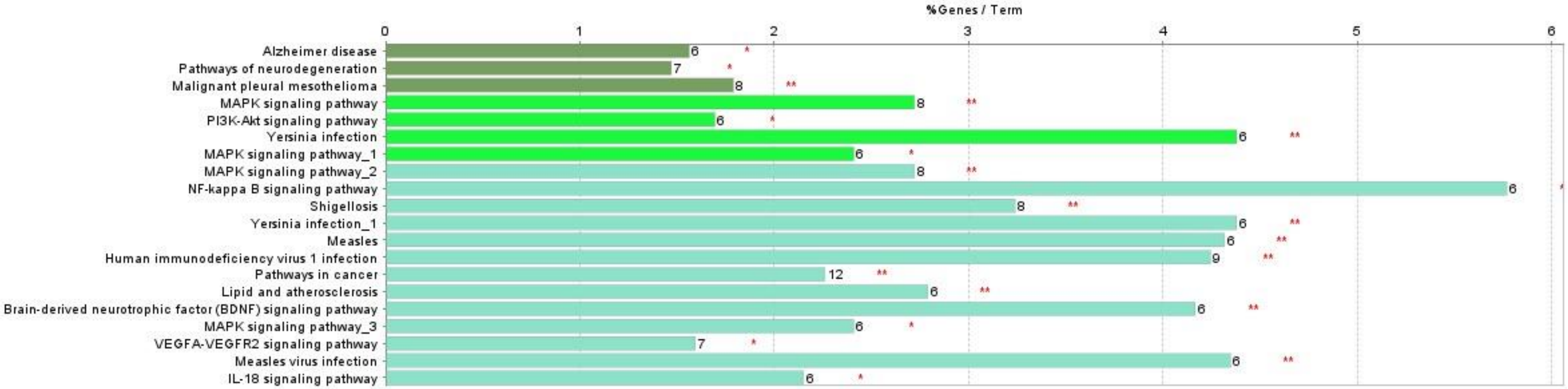

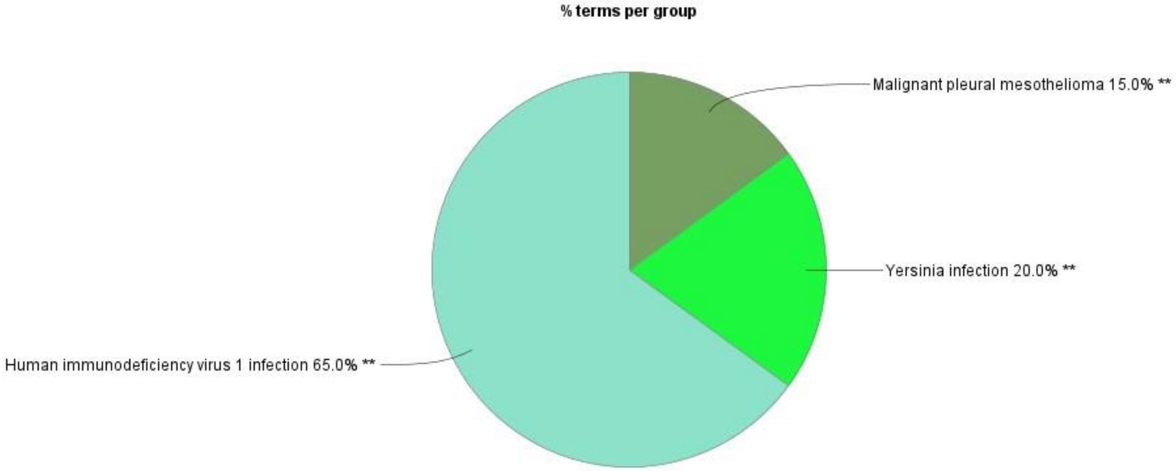

Supplementary Figure S2. Pathway enrichment analysis of miRNA target genes differentially expressed in NR vs R ccRCC patients.

Table S1. Clinical details of ccRCC cases present on the TMA and used in this study.

No.	Sex	Age	TNM Stage	Stage	Histological grade	Response (R/NR)	Follow up	RECIST	M*	SAV1	VEGFR1	BLIMP1
1	F	61	pT4N2M1	4	3	R	49	3	S	0	0	0
2	M	58	pT3N0M1	4	4	NR	17	4	S	-	-	-
3	M	57	pT3N1M1	4	4	R	-	4	S	-	0	0
4	M	68	pT3N0M1	4	4	R	54	2	S	1	0	0
5	M	54	pT3N0M0	3	4	R	49	2	M	0	0	0
6	F	60	NK	3	4	NR		NK	NK	1	0	0
7	M	56	pT3N1M0	3	4	R	-	3	M	0	0	0
8	F	63	pT3N0M0	3	3	R	61	2	M	1	0	0
9	F	26	pT3N0M1	3	4	R	75	3	S	1	1	0
10	M	56	pT3N1M1	4	4	NR	4	4	S	0	0	0
11	F	67	pT2N0M1	3	2	R	-	4	S	-	-	-
12	M	71	pT3N1M0	3	4	R	-	4	M	1	1	0
13	M	56	pT3N1M0	3	4	NR	-	4	M	0	1	0
14	M	50	pT3N1M1	4	3	R	-	2	S	1	1	0
15	F	76	pT3N0M0	3	4	R	-	2	M	1	0	0
16	M	47	pT3N0M0	3	3	R	-	4	M	1	0	0
17	M	73	pT3N0M0	2	2	NR	-	2	M	1	1	0
18	M	58	pT3N0M0	3	3	R	-	2	M	1	1	0
19	F	53	pT3N0M0	4	4	-	-	4	M	1	1	0
20	M	83	pT3N0M0	4	4	NR	-	4	M	1	1	0
21	F	45	pT1N0M0	1	3	-	17	2	M	1	0	0
22	F	80	pT3N0M0	3	3	NR	3	4	M	1	1	0
23	M	52	pT2N0M0	2	2	R	21	4	M	-	-	-

24	M	74	pT3N0M0	3	4	-	-	3	M	1	1	0
25	M	57	pT3N0M0	3	4	R	74	1	M	-	-	-
26	M	40	pT3N1M0	3	4	R	-	2	M	1	1	0
27	M	48	pT3N0M0	3	4	-	-	1	M	1	0	1
28	M	54	pT3NM	NK	4	-	-	NK	NK	0	0	0
29	M	58	pT3N0M0	3	4	-	-	4	M	1	1	0
30	M	70	pT3N0M0	3	4	R	-	1	M	0	1	1
31	M	42	pT3N0M0	3	4	-	-	2	M	0	0	1
32	M	54	pT3N2M1	4	4	NR	4	4	S	0	0	0
33	M	53	pT3N2M1	4	4	NR	1	4	S	0	1	0
34	M	61	pT1N0M0	1	3	R	79	3	M	0	0	0
35	F	48	pT3NM		2	-	-	NK	NK	1	0	0
36	F	73	pT1N1M0	3	3	R	-	1	M	1	0	0
37	M	74	pT3N0M1	4	NK	NR	-	4	S	-	-	-
38	F	56	pT3N0M0	3	4	-	-	4	M	0	0	0
39	M	53	pT3N2M1	4	4	-	-	4	S	-	-	-
40	F	55	pT3N0M1	4	3	R	-	2	S	1	1	0
41	M	70	pT3N2M1	4	4	R	-	2	S	1	1	0
42	M	64	pT3N0M0	3	2	R	-	2	M	1	0	0
43	M	59	pT1N0M0	1	3	R	72	1	M	1	0	0
44	M	46	pT3N0M0	3	4	NR	2	NK	M	1	1	0
45	F	42	pT2NM		4	-	-	NK	NK	0	1	0
46	M	82	pT3N0M0	3	4	R	-	3	M	1	1	0
47	M	43	pT3N0M0	3	4	R	-	2	M	0	1	1
48	M	68	pT3N2M1	4	3	-	-	2	S	0	0	0
49	M	66	pT2N0M0	1	3	NR	-	4	M	1	1	0
50	M	68	pT2N2M1	4	4	NR	-	3	S	-	-	-
51	M	60	pT1N0M0	1	3	R	-	3	M	0	0	0

52	M	66	pT3N0M1	4	4	NR	-	4	S	1	1	0
53	M	58	pT3N0M0	3	4	R	-	NK	M	0	0	0
54	M	66	pT3N0M0	3	3	-	-	2	M	1	0	0
55	F	55	pT3N0M0	3	2	NR	-	4	M	-	-	0
56	F	51	pT3N0M0	3	2	-	-	2	M	1	1	1
57	M	60	pT3N2M1	4	4	NR	-	4	S	-	-	-
58	M	69	pT2N0M0	2	2	R	-	2	M	1	0	0
59	M	63	pT2N0M0	2	4	-	-	4	M	1	0	0
60	M	37	pT3N0M1	4	3	R	-	3	S	-	-	0
61	M	61	pT3N0M0	3	4	R	-	4	M	1	0	0
62	M	70	pT1N0M0	1	2	R	-	3	M	0	0	0
63	M	55	pT3N0M0	3	4	R	-	2	M	-	-	0
64	M	45	pT3N0M0	3	2	R	49	2	M	1	-	0
65	F	60	pT3N1M0	3	4	R	-	4	M	0	1	0
66	M	60	pT2N0M0	2	3	NR	-	4	M	0	-	0
67	M	60	pT2N0M0	2	1	R	-	3	M	0	-	0
68	F	78	pT3N0M1	4	4	R	-	4	S	0	0	0
69	M	72	pT3N0M0	3	4	-	-	4	M	1	1	1
70	M	66	pT3N0M0	3	2	-	-	3	M	0	0	1
71	M	57	pT2N0M1	4	4	R	-	NK	S	0	0	0
72	F	52	pT3N0M1	4	3	NR	2	4	S	0	0	0
73	F	63	pT3N0M0	3	2	-	-	3	M	0	0	0
74	F	66	pT1N0M0	1	1	NR	-	3	M	1	1	0
75	F	74	pT3N0M1	4	2	R	-	1	S	0	0	0
76	M	54	pT3N1M1	4	4	NR	-	4	S	1	-	0
77	M	55	pT1N0M1	4	2	NR	-	4	S	1	-	0
78	M	77	pT3N0M0	3	2	NR	-	4	M	0	0	1
79	M	33	pT2N0M0	2	2	R	168	1	M	0	0	0

80	NK	NK	pT3N0M0	NK	4	-	-	NK	M	1	1	0
81	M	51	pT3N0M0	3	4	R	-	2	M	1	-	-
82	F	57	pT3N0M0	3	3	R	63	1	M	1	0	0
83	M	66	pT1N0M0	1	1	R	-	1	M	0	0	0
84	F	67	pT2N0M0	2	4	R	126	1	M	1	0	0
85	NK	NK	pT1N0M1	4	4	-	-	NK	S	1	0	0
86	NK	NK	NK	NK	NK	-	-	NK	NK	1	1	0
87	M	50	pT3N0M0	3	3	R	33	1	M	1	0	0
88	F	62	pT1N0M1	4	1	NR	-	4	S	1	1	0
89	F	57	pT4N0M0	4	3	NR	-	4	M	0	1	1
90	NK	NK	pT2N0M0	NK	2	-	-	NK	M	1	0	0
91	M	50	pT4N0M0	4	3	-	-	3	M	0	0	1
92	M	67	pT1N0M0	1	2	-	-	3	M	0	0	0
93	M	57	pT1N0M0	1	1	NR	-	3	M	0	0	0
94	M	47	pT4N1M1	4	NK	NR	-	4	S	0	-	0
95	NK	NK	pT2N0M0	NK	4	-	-	NK	M	0	0	0
96	F	58	pT3N0M0	3	3	R	-	NK	M	0	0	0
97	M	49	pT3N0M1	4	3	R	-	3	S	0	1	1
98	M	40	pT4N1M0	4	3	-	-	3	M	1	1	0
99	M	52	pT1N0M1	4	3	NR	19	4	S	0	1	1
100	M	77	pT3N0M1	4	3	-	-	4	S	0	1	1
101	M	61	pT3N0M1	4	3	NR	3	2	S	1	-	0
102	F	75	pT2N0M1	4	4	NR	4	4	S	0	1	0
103	M	42	pT3N0M1	4	3	R	44	4	S	1	0	0
104	M	55	pT3N0M0	3	3	-	-	3	M	1	0	0
105	F	64	pT1N0M0	1	1	-	-	3	M	0	1	0
106	M	69	pT3N0M0	3	2	R	-	3	M	0	0	0
107	NK	NK	pT3N0M0	NK	NK	-	-	NK	M	0	1	0

108	NK	NK	pT1N0M0	NK	3	-	-	NK	M	1	0	0
109	NK	NK	pT3N0M0	NK	4	-	-	NK	M	0	0	0
110	M	65	pT3N1M0	3	3	NR	4	4	M	1	0	0
111	NK	NK	pT3N0M0	NK	4	-	-	NK	M	1	1	0
112	M	73	pT4N0M0	4	2	R	-	3	M	0	0	1
113	M	64	pT3N0M1	4	2	-	-	4	S	1	0	0
114	M	71	pT3N0M0	3	2	-	-	4	M	0	0	1
115	F	51	pT3N0M1	4	3	R	-	2	S	0	0	0
116	M	57	pTNM0	NK	NK	R	-	2	M	1	1	1
117	M	65	pT3N0M1	4	3	R	33	4	S	1	0	0
118	NK	NK	pT3N0M0	NK	4	-	-	NK	M	1	1	0
119	M	76	pT3N0M1	4	2	R	-	4	S	0	1	0
120	M	67	pT3N0M1	4	4	R	-	3	S	0	0	0
121	M	54	pT4N0M0	4	3	R	-	2	M	0	0	0
122	M	76	pT3N0M0	4	4	R	-	2	M	-	-	-
123	F	70	pT1N0M1	4	4	R	-	3	S	-	-	-
124	F	60	pT3N0M1	4	4	NR	-	4	S	0	1	0
125	M	69	pT3N0M0	3	3	-	-	4	M	1	0	1
126	M	66	pT3N0M0	3	4	R	-	2	M	0	0	0
127	M	53	pT2N0M0	2	2	R	-	1	M	1	0	0
127	M	48	pT1N0M0	1	2	R	90	3	M	1	0	0
129	F	48	pT3N0M0	3	2	R	149	1	M	1	0	0
130	F	57	pT3N0M0	3	4	R	-	3	M	1	1	0
131	F	64	pT4N0M1	4	3	NR	1	4	S	0	-	-
132	F	62	pT3N0M1	4	4	NR	-	4	S	1	1	0
133	M	70	pT2N0M1	4	4	R	-	3	S	1	0	0
134	M	43	pT2N0M1	4	NK	-	-	2	S	1	1	0
135	M	63	pT4N1M1	4	4	-	-	4	S	1	1	0

136	F	65	pT1N1M1	4	4	-	-	3	S	-	-	-
137	M	64	pT3N0M1	4	2	-	-	4	S	0	0	0
138	M	57	pT1N0M0	1	4	NR	1	4	M	1	1	0
139	F	62	pT2N0M1	4	4	R	-	3	S	-	-	-
140	F	51	pT3N1M1	4	4	-	-	3	S	-	-	-
141	M	69	pT3N0M0	4	4	-	-	4	M	1	1	0
142	F	54	pT2N0M0	2	NK	-	-	4	M	1	0	0
143	M	77	pT3N0M1	4	4	-	-	4	S	0	0	0
144	M	49	pT1N0M0	1	3	R	-	1	M	-	-	-
145	M	45	pT3N0M0	3	4	NR	3	4	M	1	1	0
146	M	52	pT3N0M0	3	4	NR	-	3	M	1	0	0
147	F	50	pT3N0M1	4	4	-	-	4	S	1	1	0
148	M	64	pT1N0M0	1	2	-	-	3	M	1	1	0
149	M	58	pT3N0M1	4	3	-	-	4	S	-	-	-
150	M	68	pT1N0M0	1	2	R	47	2	M	1	0	1
151	M	63	pT3N0M1	4	3	-	-	2	S	-	-	-
152	M	59	pT3N0M0	3	3	R	-	NK	M	0	0	0
153	NK	NK	NK	NK	NK	NK	-	NK	NK	0	1	0
154	F	72	pT3N0M1	4	3	NR	-	3	S	1	0	0
155	M	60	pT3N0M0	3	4	R	-	2	M	0	1	1
156	M	51	pT3N0M1	4	4	-	-	2	S	0	0	1
157	M	49	pT3N0M0	3	3	R	-	4	M	1	0	0
158	F	43	pT1N0M0	1	3	R	-	1	M	1	0	0
159	M	54	pT3N0M0	3	2	NR	-	4	M	0	0	0
160	F	52	pT2N0M1	4	4	R	-	3	S	0	0	0
161	M	60	pT3N0M1	4	2	R	-	1	S	0	0	0
162	M	58	pT3N0M1	4	4	-	-	2	S	0	0	0
163	F	73	pT3N0M1	4	4	NR	-	4	S	0	0	0

164	F	40	pT3N0M0	3	4	-	-	4	M	1	1	0
165	M	53	pT1N0M1	4	4	NR	1	4	S	1	0	0
166	M	72	pT3N0M1	4	3	R	-	2	S	1	1	0
167	NK	NK	pT3N0M0	NK	2	NK	-	NK	M	1	0	0
168	NK	NK	pT2N0M0	NK	2	NK	-	NK	M	0	0	0
169	NK	NK	NK	NK	NK	NK	-	NK	NK	0	0	0
170	NK	NK	pT1N0M0	NK	4	NK	-	NK	M	1	0	0
171	M	68	NK	3	NK	NR	-	NK	NK	-	-	-
172	M	57	NK	1	NK	R	-	NK	NK	-	-	-
173	M	57	NK	3	NK	NR	-	NK	NK	-	-	-
174	M	43	NK	NK	3	R	-	NK	NK	-	-	-

*S = synchronous metastasis; M = metachronous metastasis. RECIST score: 1. Complete response, 2. Partial response, 3. Stable disease, 4. Progression

Supplementary Table S2. List of miRNAs commonly differentially expressed between NR and R.

miRNA	NR intensity	R intensity	Fold Change	P-val	Chromosome	Start	Stop
miR-1290	7.01	3.19	14.17	0.0161	chr1	19223572	19223590
miR-31-5p	11.76	8.38	10.43	0.0022	chr9	21512157	21512177
miR-183-5p	5.8	2.59	9.22	0.0314	chr7	1.29E+08	1.29E+08
miR-297	5.95	2.95	8.02	0.002	chr4	1.12E+08	1.12E+08
miR-3135b	10.81	7.84	7.84	0.0457	chr6	32717729	32717750
miR-205-5p	4.8	1.92	7.36	0.02	chr1	2.1E+08	2.1E+08
miR-223-3p	8	5.18	7.07	0.0214	chrX	65238779	65238800
miR-200c-3p	10.56	7.8	6.78	0.0033	chr12	7072905	7072927
miR-4786-3p	5.75	3.06	6.44	0.0361	chr2	2.41E+08	2.41E+08
miR-154-5p	4.26	1.73	5.76	0.0035	chr14	1.02E+08	1.02E+08
miR-182-5p	9.32	6.88	5.43	0.0048	chr7	1.29E+08	1.29E+08
miR-200b-3p	5.92	3.66	4.77	0.0146	chr12	2.4E+08	2.4E+08
miR-452-5p	6.56	4.31	4.76	0.0002	chrX	1.51E+08	1.51E+08
miR-141-3p	4.28	2.08	4.6	0.0093	chr12	7073318	7073339
miR-382-5p	7.45	5.3	4.45	9.09E-05	chr14	1.02E+08	1.02E+08
miR-200a-5p	7.05	4.94	4.32	0.002	chr1	1167863	1167952
miR-6836-5p	5.78	3.74	4.11	0.0269	chr7	2297186	2297207
miR-224-5p	5.75	3.76	3.98	0.0268	chrX	1.51E+08	1.51E+08
miR-138-5p	9.39	7.41	3.95	0.0185	chr16	56892439	56892461
miR-6754-3p	4.45	2.55	3.73	0.0008	chr11	71184592	71184613
miR-429	6.54	4.71	3.55	0.0144	chr12	1169005	1169087
miR-4793-3p	4.25	2.44	3.52	0.0159	chr3	48681634	48681656
miR-146b-3p	5.43	3.62	3.51	0.0003	chr10	1.04E+08	1.04E+08
miR-493-3p	4.01	2.2	3.51	0.001	chr14	1.01E+08	1.01E+08
miR-21-3p	8.66	6.87	3.45	0.0011	chr17	57918672	57918692
miR-195-3p	4.22	2.48	3.34	0.0084	chr17	6920947	6920968
miR-550a-3p	5.93	4.21	3.3	0.0429	chr7	29720364	29720385
miR-629-5p	8.4	6.81	3.01	0.008	chr15	70371766	70371786
miR-125b	6.28	4.69	3	0.0021	chr11	1.22E+08	1.22E+08
miR-574-5p	4.37	2.83	2.91	0.001	chr4	38869677	38869699
miR-4502	4.14	2.61	2.89	0.0136	chr13	1.15E+08	1.15E+08
miR-134-5p	7.36	5.83	2.88	0.0004	chr14	1.02E+08	1.02E+08
miR-193a-3p	7.57	6.05	2.87	0.0049	chr17	29887069	29887090
miR-409-5p	4.35	2.83	2.87	0.0232	chr14	1.02E+08	1.02E+08
miR-766-3p	5.4	3.94	2.76	0.0204	chrX	1.19E+08	1.19E+08
miR-18a-3p	5.24	3.77	2.76	0.002	chr13	92003051	92003073
miR-409-3p	7.67	6.23	2.71	0.0019	chr14	1.02E+08	1.02E+08
miR-433-3p	4.41	2.98	2.69	0.007	chr14	1.01E+08	1.01E+08
miR-432-5p	7.31	5.9	2.66	0.0009	chr14	1.01E+08	1.01E+08
miR-337-5p	5.49	4.1	2.61	0.0076	chr14	1.01E+08	1.01E+08
miR-106b-3p	8.73	7.36	2.59	0.0058	chr7	99691625	99691646
miR-760	4.64	3.28	2.56	0.0011	chr1	94312436	94312455

miR-99a-3p	3.81	2.45	2.56	0.0228	chr21	17911458	17911479
miR-3185	9.24	7.89	2.55	0.0178	chr17	46801808	46801830
miR-155-5p	13.02	11.69	2.51	0.0063	chr21	26946295	26946317
miR-671-5p	7.54	6.22	2.5	3.69E-05	chr7	1.51E+08	1.51E+08
miR-629-3p	5.73	4.41	2.49	0.0356	chr15	70371726	70371747
miR-17-3p	7.77	6.46	2.48	0.0038	chr13	92002909	92002930
miR-19a-3p	5.2	3.89	2.48	0.0047	chr13	92003193	92003215
miR-425-3p	7.32	6.01	2.47	0.0029	chr3	49057592	49057613
miR-766-5p	3.83	2.52	2.47	0.0047	chrX	1.19E+08	1.19E+08
miR-379-5p	6.85	5.58	2.43	0.002	chr14	1.01E+08	1.01E+08
miR-1285-3p	6.65	5.37	2.43	0.0059	chr2	70480065	70480086
miR-146b-5p	10.02	8.75	2.42	0.0038	chr10	1.04E+08	1.04E+08
miR-1301-3p	8.13	6.87	2.39	0.0048	chr2	25551520	25551543
miR-381-3p	4.43	3.21	2.33	0.009	chr14	1.02E+08	1.02E+08
miR-487a-5p	3.06	1.84	2.33	0.0059	chr14	1.02E+08	1.02E+08
miR-592	3.35	2.14	2.31	0.0154	chr7	1.27E+08	1.27E+08
miR-330-3p	7.86	6.67	2.29	0.008	chr19	46142267	46142289
miR-127-5p	3.57	2.38	2.28	0.0091	chr14	1.01E+08	1.01E+08
miR-3064-5p	3.4	2.22	2.26	0.0097	chr17	62496935	62496955
miR-487b-3p	6.73	5.55	2.26	0.0193	chr14	1.02E+08	1.02E+08
miR-127-3p	9.04	7.87	2.25	0.0049	chr14	1.01E+08	1.01E+08
miR-6877-3p	3.55	2.39	2.23	0.0097	chr9	1.36E+08	1.36E+08
miR-28-3p	10.13	8.99	2.21	0.0028	chr3	1.88E+08	1.88E+08
miR-874-3p	7.39	6.25	2.21	0.0085	chr5	1.37E+08	1.37E+08
miR-492	3.36	2.22	2.21	0.0021	chr12	95228203	95228225
miR-5095	4.79	3.65	2.2	0.029	chr1	53400608	53400628
miR-125b	8.11	6.97	2.2	0.008	chr21	17962610	17962631
miR-1180-3p	7.37	6.24	2.18	0.0014	chr17	19247826	19247847
miR-3615	5.25	4.13	2.17	0.0023	chr17	72744802	72744822
miR-331-5p	6.51	5.39	2.17	0.0199	chr12	95702221	95702242
miR-130b-3p	10.1	8.99	2.16	0.0117	chr22	22007643	22007664
miR-503-5p	9.41	8.3	2.15	0.0096	chrX	1.34E+08	1.34E+08
miR-1244	5.03	3.93	2.15	0.0452	chr12	9392068	9392093
miR-24-2-5p	7.58	6.48	2.14	0.0361	chr19	13947140	13947161
miR-4436b	4.69	3.59	2.14	0.0054	chr2	1.11E+08	1.11E+08
miR-93-3p	3.92	2.84	2.12	0.0331	chr7	99691400	99691421
miR-1254	4.66	3.58	2.11	3.70E-05	chr10	23682337	23682360
miR-3176	5.7	4.63	2.09	0.0448	chr16	593336	593354
miR-4474-3p	3.09	2.03	2.09	0.0177	chr9	20502275	20502296
miR-4538	4.08	3.03	2.07	0.0048	chr14	1.06E+08	1.06E+08
miR-339-5p	8.64	7.59	2.07	0.0139	chr7	1062626	1062648
miR-485-3p	3.75	2.73	2.03	0.0278	chr14	1.02E+08	1.02E+08
miR-424-3p	8.05	7.05	2.01	0.0288	chrX	1.34E+08	1.34E+08
miR-421	7.69	6.68	2.01	0.0094	chrX	73438227	73438249
miR-3184-3p	4.06	3.06	2.01	0.0026	chr17	28444113	28444135
miR-6796-3p	3.5	2.52	1.98	0.0191	chr19	40875794	40875814
miR-3651	10.07	9.08	1.98	0.0191	chr9	95054743	95054766
miR-133a-3p	3.53	2.55	1.97	0.0177	chr18	19405673	19405694

miR-769-3p	5.96	5	1.94	0.0338	chr19	46522258	46522280
miR-6857-5p	3.34	2.39	1.93	0.0105	chrX	53432671	53432692
miR-138-1-3p	4.91	3.97	1.92	0.0281	chr3	44155766	44155787
miR-4539	6.15	5.22	1.9	0.0036	chr14	1.06E+08	1.06E+08
miR-28-5p	10.58	9.67	1.88	0.0061	chr3	1.88E+08	1.88E+08
miR-1184	6.24	5.33	1.87	0.0246	chrX	1.54E+08	1.54E+08
miR-99b-3p	7.65	6.75	1.86	0.0128	chr19	52195909	52195930
miR-425-5p	10.59	9.7	1.86	0.0041	chr3	49057632	49057654
miR-550a	5.76	4.9	1.83	0.0133	chr7	29720405	29720424
miR-6511a	5.11	4.24	1.83	0.0274	chr16	15019837	15019858
miR-887-3p	5.92	5.05	1.82	0.0275	chr5	15935338	15935359
miR-3689a	3.11	2.24	1.82	0.0088	chr9	1.38E+08	1.38E+08
miR-376c-3p	4.34	3.49	1.81	0.0248	chr14	1.02E+08	1.02E+08
miR-584-5p	4.65	3.8	1.81	0.0245	chr5	1.48E+08	1.48E+08
miR-330-5p	3.32	2.47	1.8	0.0157	chr19	46142307	46142328
miR-1270	8.25	7.41	1.79	0.039	chr19	20510129	20510151
miR-4647	3.29	2.46	1.77	0.0074	chr6	44221990	44222012
miR-6762-3p	2.78	1.95	1.77	0.0157	chr12	1.14E+08	1.14E+08
miR-99a-5p	12.73	11.92	1.76	0.0433	chr21	17911421	17911442
miR-320e	8.59	7.79	1.74	0.027	chr19	47212551	47212568
miR-8055	3.06	2.26	1.74	0.0466	chr8	6479655	6479677
miR-1238-3p	4.13	3.34	1.73	0.0164	chr19	10662859	10662878
miR-339-3p	9.02	8.22	1.73	0.011	chr7	1062591	1062613
miR-1915-5p	3.23	2.45	1.72	0.004	chr10	21785538	21785559
miR-431-5p	2.71	1.96	1.68	0.0496	chr14	1.01E+08	1.01E+08
miR-1913	3.11	2.36	1.68	0.044	chr6	1.67E+08	1.67E+08
miR-6805-3p	4.19	3.44	1.68	0.0267	chr19	55899588	55899610
miR-1250-5p	3.41	2.67	1.66	0.0019	chr17	79107065	79107085
miR-652-3p	9.63	8.93	1.63	0.0004	chrX	1.09E+08	1.09E+08
miR-21-5p	11.69	10.98	1.63	0.0404	chr17	57918634	57918655
miR-2277-3p	3.33	2.63	1.62	0.0037	chr5	92956416	92956436
miR-4455	3	2.31	1.61	0.0255	chr4	1.86E+08	1.86E+08
miR-632	2.08	1.39	1.61	0.0176	chr17	30677188	30677206
miR-370-3p	5.84	5.15	1.61	0.0261	chr14	1.01E+08	1.01E+08
miR-637	4.22	3.53	1.61	0.0343	chr19	3961427	3961450
miR-27b-5p	6.48	5.81	1.6	0.0455	chr9	97847745	97847766
miR-6772-5p	5.08	4.41	1.59	0.0103	chr16	57806238	57806259
miR-6508-5p	3.16	2.51	1.57	0.0063	chr21	40818940	40818960
miR-3198	3.31	2.65	1.57	0.0348	chr12	54625191	54625212
miR-216a-5p	3	2.35	1.56	0.0047	chr2	56216155	56216176
miR-608	2.73	2.08	1.56	0.0127	chr10	1.03E+08	1.03E+08
miR-377-5p	2.53	1.9	1.55	0.0349	chr14	1.02E+08	1.02E+08
miR-7706	2.41	1.78	1.55	0.0174	chr15	85923870	85923893
miR-4481	4.18	3.56	1.54	0.0286	chr10	12695180	12695196
miR-4265	2.41	1.78	1.54	0.0252	chr2	1.1E+08	1.1E+08
miR-501-5p	3.76	3.13	1.54	0.0298	chrX	49774343	49774364
miR-151a-3p	10.83	10.22	1.52	0.0024	chr8	1.42E+08	1.42E+08
miR-6893-3p	3.58	2.97	1.52	0.0403	chr8	1.46E+08	1.46E+08

miR-23b-5p	6.38	5.78	1.51	0.0306	chr9	97847509	97847530
miR-191-3p	4.4	3.81	1.51	0.0314	chr3	49058064	49058085
miR-3928-3p	3.54	2.95	1.51	0.0351	chr22	31556048	31556069
miR-5584-5p	3.42	4.01	-1.5	0.018	chr1	45011165	45011186
miR-3126-5p	3.67	4.27	-1.51	0.0278	chr2	69330823	69330844
miR-642b-3p	6.36	6.95	-1.51	0.0401	chr19	46178199	46178220
miR-6881-5p	4.41	5	-1.51	0.0251	chr15	74703747	74703768
miR-6841-3p	1.72	2.32	-1.52	0.0143	chr8	24811310	24811330
miR-6892-5p	4.54	5.15	-1.52	0.0391	chr7	1.43E+08	1.43E+08
miR-6769b	7.48	8.08	-1.52	0.0029	chr1	2.07E+08	2.07E+08
miR-765	5.08	5.69	-1.53	0.0176	chr1	1.57E+08	1.57E+08
miR-6803-5p	12.24	12.86	-1.53	0.0267	chr19	55756592	55756613
miR-197-5p	7.38	7.99	-1.53	0.0318	chr1	1.1E+08	1.1E+08
miR-6716-3p	1.85	2.48	-1.54	0.0027	chr11	1.19E+08	1.19E+08
miR-6716-5p	7.51	8.14	-1.55	0.0064	chr11	1.19E+08	1.19E+08
miR-6750-3p	2.07	2.7	-1.55	0.0154	chr11	64665835	64665855
miR-3925-5p	3.42	4.05	-1.55	0.048	chr6	36590257	36590278
miR-6786-5p	11.72	12.36	-1.56	0.0402	chr17	79660839	79660859
miR-6802-5p	6.97	7.61	-1.57	0.0483	chr19	55751320	55751339
miR-761	1.97	2.62	-1.57	0.0119	chr1	52302047	52302068
miR-6880-5p	7.04	7.69	-1.57	0.0112	chr12	1.25E+08	1.25E+08
miR-4330	1.67	2.32	-1.58	0.003	chrX	1.5E+08	1.5E+08
miR-6868-5p	3.18	3.85	-1.58	0.0435	chr17	74094131	74094152
miR-8074	2.7	3.36	-1.58	0.039	chr19	51710195	51710218
miR-4463	10.99	11.67	-1.59	0.0141	chr6	76138162	76138178
miR-4695-5p	7.89	8.59	-1.63	0.0056	chr1	19209744	19209765
miR-6775-5p	9.41	10.13	-1.65	0.0206	chr16	87868237	87868261
miR-4314	3.44	4.17	-1.66	0.0349	chr17	7991384	7991401
miR-4743-5p	6.33	7.06	-1.66	0.0095	chr18	46196973	46196995
miR-642a-3p	6.76	7.5	-1.67	0.0495	chr19	46178236	46178257
miR-3141	8.7	9.44	-1.68	0.014	chr5	1.54E+08	1.54E+08
miR-542-3p	1.82	2.58	-1.69	0.0447	chrX	1.34E+08	1.34E+08
miR-5739	7.3	8.06	-1.7	0.0499	chr22	28855913	28855932
miR-204-3p	7.88	8.65	-1.7	0.0227	chr9	73424909	73424929
miR-6837-5p	4.71	5.5	-1.73	0.0029	chr7	44091370	44091390
miR-556-3p	1.9	2.69	-1.73	0.04	chr1	1.62E+08	1.62E+08
miR-5703	5.38	6.18	-1.74	0.0401	chr2	2.28E+08	2.28E+08
miR-6794-5p	8.61	9.42	-1.75	0.0267	chr19	12963079	12963098
miR-138-2-3p	2.17	2.97	-1.75	0.0173	chr16	56892486	56892507
miR-4436b	5.52	6.34	-1.77	0.0305	chr2	1.11E+08	1.11E+08
miR-4436a	5.37	6.2	-1.78	0.027	chr2	89111939	89111959
miR-4475	2.46	3.29	-1.78	0.0306	chr9	36823539	36823560
miR-4468	3.82	4.66	-1.8	0.0007	chr7	1.38E+08	1.38E+08
miR-524-5p	1.69	2.53	-1.8	0.0493	chr19	54214271	54214292
miR-7977	12.54	13.4	-1.81	0.0433	chr3	1.76E+08	1.76E+08
miR-3156-3p	1.8	2.66	-1.81	0.0249	chr10	45659510	45659530
miR-4257	4.96	5.84	-1.83	0.009	chr1	1.51E+08	1.51E+08
miR-7975	11.87	12.74	-1.83	0.0469	chr19	55634593	55634610

miR-4514	4.14	5.02	-1.84	0.0148	chr15	81289791	81289808
miR-6798-5p	8.46	9.34	-1.84	0.0298	chr19	49513168	49513190
miR-3945	4.12	5.01	-1.85	0.0036	chr4	1.86E+08	1.86E+08
miR-6885-5p	3.93	4.82	-1.85	0.0341	chr19	6389685	6389709
miR-4773	4.88	5.78	-1.86	0.0128	chr2	1.52E+08	1.52E+08
miR-5000-3p	2.26	3.2	-1.92	0.0144	chr2	75318000	75318021
miR-7515	4.47	5.42	-1.93	0.0285	chr2	6790550	6790567
miR-6819-5p	7.65	8.61	-1.95	0.0272	chr22	36682927	36682948
miR-6165	7.42	8.4	-1.97	0.0134	chr17	47588187	47588205
miR-6788-5p	3.96	4.95	-1.99	0.0297	chr18	10759622	10759642
miR-6808-5p	7.31	8.32	-2	0.0171	chr1	1275062	1275083
miR-6877-5p	6.27	7.26	-2	0.0096	chr9	1.36E+08	1.36E+08
miR-328-5p	10.38	11.38	-2	0.0426	chr16	67236270	67236292
miR-4428	6.98	8	-2.03	0.0258	chr1	2.38E+08	2.38E+08
miR-4328	2.59	3.63	-2.05	0.0201	chrX	78156701	78156717
miR-1286	2.9	3.97	-2.09	0.0135	chr22	20236668	20236688
miR-6760-5p	5.48	6.54	-2.1	0.0124	chr12	1.12E+08	1.12E+08
miR-4694-3p	2.88	3.96	-2.11	0.0026	chr11	19781559	19781579
miR-513c-5p	2.03	3.12	-2.12	0.005	chrX	1.46E+08	1.46E+08
miR-583	1.9	3	-2.15	0.0123	chr5	95414857	95414877
miR-4781-5p	2.52	3.63	-2.16	0.0064	chr1	54519764	54519784
miR-6766-5p	3.22	4.36	-2.2	0.0171	chr15	89870013	89870036
miR-4667-5p	7.27	8.44	-2.26	0.0104	chr9	35608093	35608114
miR-4261	5.42	6.62	-2.3	0.0193	chr2	10332750	10332765
miR-4708-3p	3.59	4.81	-2.34	0.0056	chr14	65801841	65801862
miR-942-3p	3.35	4.59	-2.36	0.0298	chr1	1.18E+08	1.18E+08
miR-6879-5p	8.02	9.27	-2.36	0.0212	chr11	64785982	64786003
miR-4445-3p	3.66	5.09	-2.7	0.0421	chr3	1.09E+08	1.09E+08
miR-3153	3.61	5.08	-2.77	0.0003	chr9	91927189	91927211
miR-4448	4.76	6.24	-2.79	0.0077	chr3	1.84E+08	1.84E+08
miR-126-5p	2.64	4.24	-3.04	0.0107	chr9	1.4E+08	1.4E+08
miR-570-3p	2.33	4.16	-3.55	0.0049	chr3	1.95E+08	1.95E+08
miR-3144-5p	1.95	3.82	-3.67	1.46E-05	chr6	1.2E+08	1.2E+08
miR-32-5p	4.12	6.29	-4.49	0.0399	chr9	1.12E+08	1.12E+08

Supplementary Table S3. List of pre-miRNAs differentially expressed between NR and R.

pre-miRNA	NR intensity	R intensity	Fold Change	P-val	Chromosome	Start	Stop
mir-297	4.71	2.95	3.39	0.0045	chr4	1.12E+08	1.12E+08
mir-4525	3.3	1.88	2.67	0.0113	chr17	80626109	80626183
mir-8061	3.24	1.95	2.44	0.0045	chr19	55156760	55156834
mir-21	7.22	6.01	2.31	0.0057	chr17	57918627	57918698
mir-3651	6.99	5.81	2.26	0.0031	chr9	95054740	95054829
mir-4730	2.8	1.76	2.06	0.0029	chr17	78393218	78393293
mir-4539	4.27	3.26	2.01	0.0013	chr14	1.06E+08	1.06E+08
mir-3689a	2.84	1.84	1.99	0.0425	chr9	1.38E+08	1.38E+08
mir-339	4	3.04	1.95	0.0003	chr7	1062569	1062662
mir-1299	3.53	2.62	1.89	0.0176	chr9	69002239	69002321
mir-346	2.88	1.98	1.86	0.0049	chr10	88024451	88024545
mir-28	5.17	4.33	1.8	0.0055	chr3	1.88E+08	1.88E+08
mir-6886	4.44	3.65	1.73	0.0035	chr19	11224150	11224210
mir-142	3.36	2.6	1.7	0.0008	chr17	56408593	56408679
mir-92b	4.55	3.79	1.7	0.0331	chr1	1.55E+08	1.55E+08
mir-5095	7.63	6.87	1.69	0.0476	chr1	53400602	53400689
mir-424	4.86	4.12	1.67	0.0231	chrX	1.34E+08	1.34E+08
mir-3654	2.28	1.54	1.67	0.0192	chr7	1.33E+08	1.33E+08
mir-4734	4.45	3.71	1.67	0.0228	chr17	36858515	36858584
mir-125b-2	2.6	1.87	1.66	0.0102	chr21	17962557	17962645
mir-550a-1	5.29	4.56	1.66	0.0107	chr7	30329410	30329506
mir-550a-2	5.29	4.56	1.66	0.0107	chr7	32772593	32772689
mir-550a-3	5.29	4.56	1.66	0.0107	chr7	29720350	29720444
mir-941-1	6.21	5.48	1.65	0.0326	chr20	62550802	62550873
mir-941-2	6.21	5.48	1.65	0.0326	chr20	62550858	62550929
mir-941-3	6.21	5.48	1.65	0.0326	chr20	62550914	62550985
mir-941-4	6.21	5.48	1.65	0.0326	chr20	62551109	62551180
mir-4442	3.14	2.43	1.64	0.0062	chr3	25706364	25706430
mir-6516	7.06	6.36	1.63	0.0156	chr17	75085499	75085579
mir-6836	5.59	4.9	1.61	0.0128	chr7	2297150	2297212
mir-6796	3.07	2.4	1.59	0.0039	chr19	40875753	40875814
mir-425	3.86	3.21	1.57	0.0051	chr3	49057581	49057667
mir-409	2.74	2.09	1.57	0.0006	chr14	1.02E+08	1.02E+08
mir-503	2.95	2.3	1.57	0.016	chrX	1.34E+08	1.34E+08
mir-22	2.66	2.02	1.55	0.0322	chr17	1617197	1617281
mir-769	2.75	2.12	1.55	0.0036	chr19	46522190	46522307
mir-6770-1	2.57	1.96	1.53	0.0053	chr16	15024677	15024736
mir-6770-2	2.57	1.96	1.53	0.0053	chr16	16423162	16423221
mir-6770-3	2.57	1.96	1.53	0.0053	chr16	18473208	18473267
mir-642a	3.86	3.25	1.52	0.0065	chr19	46178186	46178282

mir-4537	5.08	4.48	1.52	0.0203	chr14	1.06E+08	1.06E+08
mir-8078	3.62	3.01	1.52	0.0108	chr18	112256	112339
mir-4512	2.05	2.64	-1.5	0.0015	chr15	66789296	66789372
mir-513c	1.81	2.41	-1.51	0.0119	chrX	1.46E+08	1.46E+08
mir-548i-4	1.87	2.48	-1.53	0.0228	chrX	83480760	83480836
mir-3154	6.6	7.26	-1.58	0.0142	chr9	1.31E+08	1.31E+08
mir-548i-4	1.77	2.5	-1.66	0.0153	chrX	83480760	83480836
mir-3144	2.17	2.95	-1.71	0.0002	chr6	1.2E+08	1.2E+08
mir-7977	3.37	4.24	-1.84	0.0004	chr3	1.76E+08	1.76E+08
mir-1181	2.65	3.61	-1.95	0.0203	chr19	10514134	10514214
mir-548a-1	4.59	5.63	-2.06	0.0452	chr6	18572015	18572111
mir-548q	3.79	5.27	-2.79	0.0396	chr10	12767253	12767352

Supplementary Table S4. List of lncRNA differentially expressed between NR and R.

ID	NR intensity	R intensity	Fold Change	P-val	Chromosome	Cytoband
RP11-26E5.1; zashor	5.35	3.83	2.88	0.0001	chr4	
RP11-298O21.6	7.05	5.93	2.17	0.0516	chr1	
RP11-368I23.4	4.99	3.9	2.13	0.0003	chr4	
RP11-624L4.1; RP11-27M9.1; meepor	4.44	3.35	2.12	0.0842	chr8	
SNORD3B-2	7.73	6.65	2.11	0.0383	chr17	
KB-1299A7.2	4.53	3.47	2.08	0.0855	chr2	2p12
RP11-733C7.1	5.22	4.17	2.07	0.0192	chr3	
RP11-532L16.3	4.77	3.73	2.07	0.0057	chr7	7q31.3
RP4-591L5.1	4.58	3.56	2.03	0.0001	chr13	13q21.31
RP11-46I8.3	5.27	4.27	2	0.0045	chr20	
RP11-530I17.1	4.63	3.64	1.99	0.0005	chr16	
RP3-323A16.1	5.23	4.25	1.97	0.002	chr1	
RP11-114O18.1	5.43	4.47	1.95	0.0139	chr3	
CTD-2333M24.1	4.22	3.27	1.94	0.0055	chr10	10p14
LINC00358	4.64	3.69	1.94	0.0052	chr19	
LOC101927513	4.62	3.67	1.94	0.0081	chr21	
RP11-202G11.2; gofo	5.4	4.46	1.93	0.0395	chr3	3p14.2
LOC10192	4.3	3.37	1.91	0.0209	chr15	
RP3-414A15.11	4.22	3.29	1.91	0.1732	chr3	
RP11-480O10.1	3.97	3.05	1.9	0.0432	chr11	
RP11-1102P16.1	4.76	3.84	1.89	0.0018	chr11	
RP11-201A3.1	4.13	3.21	1.88	0.0876	chr18	
RP11-255H23.4	4.99	4.08	1.88	0.0488	chr2	2p21
LINC01609	5.05	4.14	1.87	0.0121	chr8	
; RP11-251P6.1; vawda	4.43	3.52	1.87	0.0071	chr5	
CTC-527H23.2	4.78	3.89	1.86	0.09	chr2	
IFNG-AS1	4.41	3.52	1.86	0.0515	chr17	
GS1-519E5.1; haro	4.39	3.51	1.84	0.0878	chr16	
LINC01214	5.23	4.36	1.84	0.006	chr20	
RP11-221N13.2	5.06	4.18	1.84	0.006	chr11	
RP11-779P15.2; moyswoyby	5.35	4.49	1.82	0.1002	chr1	1p21.1
RP11-84C10.3; flarcharbu	6.81	5.95	1.82	0.0051	chr12	
RP11-272K23.3	5.03	4.17	1.8	0.0123	chr2	2p23.3
CTD-2036A18.2	4.06	3.22	1.79	0.0022	chr3	
RP11-348M17.2	4.11	3.28	1.78	0.0058	chr16	16p13.3
RP11-439I14.2; swoysmee	5.29	4.46	1.78	0.0151	chr5	
RP11-820I16.1; platerbu	5.11	4.27	1.78	0.1274	chr3	
RP11-162D9.3; sareya	4.06	3.23	1.77	0.0184	chr15	
RP11-126K1.8; skorporbo	4.47	3.64	1.77	0.0054	chr1	
CTD-3096P4.1; rycher	4.34	3.52	1.76	0.0087	chr5	
AC127904.2	6.83	6.01	1.76	0.0057	chr8	
CTC-467M3.2	5.86	5.06	1.75	0.3275	chr2	

CTD-2194L12.2	5.03	4.23	1.75	0.1061	chr3	
RP11-561O23.7	4.87	4.07	1.75	0.0159	chr18	
CYB561D2	4.36	3.55	1.75	0.0044	chr16	
OFD1P6Y	4.41	3.62	1.74	0.1948	chr1	1q43
MEAT6	4.04	3.25	1.73	0.0103	chr12	12q15
RP5-1050D4.5	5.38	4.59	1.73	0.2737	chr9	
AC007392.4; jerdee	4.23	3.44	1.73	0.0988	chr20	
RFPL4B	4.29	3.5	1.73	0.074	chr1	
RP11-163M18.1; noyloyby	3.77	2.98	1.73	0.0051	chr12	
RP11-566H8.1	4.28	3.48	1.73	0.0132	chr8	
RP11-556I13.2	4.31	3.53	1.72	0.0097	chr10	10p14
RP11-275I4.2	3.62	2.84	1.72	0.0049	chr13	
RP11-727F15.13	6.82	6.03	1.72	0.1287	chr21	
SIDT1-AS1; AC055740.2	4.74	3.95	1.72	0.0161	chr13	
KCNAB1-AS2	4.68	3.91	1.71	0.0644	chr5	
RP11-212E8.1	4.43	3.65	1.71	0.2983	chr14	14q12
RP11-1079H9.1	4.45	3.69	1.7	0.0018	chr2	2p14
RP1-76B20.11; RP1-76B20.12	5.01	4.25	1.7	0.0064	chr15	
RP11-351C21.2	5.15	4.39	1.7	0.027	chr12	
RP4-620E11.8	6.11	5.34	1.7	0.2362	chr21	
LINC00879	4.68	3.91	1.7	0.0501	chr3	
bypa; LINC01074	4.27	3.52	1.69	0.0699	chr8	
RP1-34H18.1; RP1-97G4.1	4.08	3.32	1.69	0.1712	chr15	
CTC-332L22.1	4	3.24	1.69	0.0437	chr10	
SLFNL1-AS1	3.93	3.18	1.68	0.005	chr3	3p25.3
LOC101929341; RP11-26M5.2	4.11	3.36	1.68	0.0371	chrX	
RP11-47L3.1; LOC100287278	5.15	4.41	1.67	0.0486	chr18	18p11.21
RP11-125O18.1	4.3	3.56	1.67	0.0562	chr22	
RP11-317J19.1	4.21	3.47	1.67	0.0419	chr3	
CTD-3028N15.3	7.78	7.04	1.67	0.0533	chr13	13q22.3
RP11-94C24.11; klorvorbu	6.13	5.39	1.67	0.0561	chr5	
LOC101928241	3.97	3.25	1.66	0.0526	chr8	8q23
RP11-813F20.2	4.29	3.56	1.66	0.1521	chr11	
RP3-324O17.8; RNU6-384P	4.62	3.89	1.66	0.0047	chr5	
LOC101928909	4.1	3.38	1.65	0.2227	chr16	
CTD-2280E9.1; lorfley	4.17	3.45	1.65	0.2618	chr6	6p22.1
LOC105375734	5.89	5.18	1.64	0.0139	chrY	Yq11.22
AC004447.2; himere	3.95	3.24	1.64	0.0258	chr5	
AC016722.3	4.21	3.5	1.64	0.0689	chr12	
RP11-314P15.2; perswey	5.39	4.68	1.64	0.0029	chr11	
LINC00904	4.5	3.79	1.64	0.0178	chr13	13q34
APOC4-APOC2; APOC2	5.12	4.4	1.64	0.1398	chr9	9p11.2
AP000855.4	6.48	5.76	1.64	0.6777	chr9	9p13.3
TTTY1; TTTY1B	4.4	3.7	1.63	0.0064	chr9	
CTB-181F24.1	5.26	4.55	1.63	0.007	chr3	
RORA-AS2	5.33	4.62	1.63	0.0874	chr6	6q21

CTD-2007A10.1	4.96	4.25	1.63	0.1493	chr17	
RP4-620F22.2	4.68	3.98	1.62	0.0292	chr5	
C1orf143	4.51	3.82	1.62	0.0327	chr3	3q26.32
LOC10272455	4.69	3.99	1.62	0.0024	chr8	8q24.22
CNTN4-AS2	4.97	4.28	1.62	0.0015	chr9	
RP11-44K6.3	3.69	3.01	1.61	0.1604	chr17	
RP11-445P17.8	4.47	3.78	1.61	0.7322	chr13	13q12.12
RP11-176F3.7	5.04	4.35	1.61	0.0504	chr14	
RP11-693L9.2	3.98	3.3	1.61	0.0231	chr16	
RP11-454P21.1; stogor	3.89	3.2	1.61	0.0157	chr12	
RP11-384C4.7	4.52	3.83	1.61	0.1087	chr12	
LINC01430	4.69	4	1.61	0.0217	chr22	
ZNF20	4.57	3.9	1.6	0.0056	chr10	
RP11-513D5.2	4.7	4.02	1.6	0.0094	chr17	
LOC102724661	3.94	3.26	1.6	0.1702	chr18	
CTB-33G10.6	4.28	3.6	1.6	0.1193	chr12	
AC018832.1	4	3.32	1.6	0.0524	chr6	6q25.2
AC100802.3	4.08	3.4	1.6	0.9357	chrX	
RP11-351A20.1; moyru	4.72	4.04	1.6	0.0065	chr5	
NALCN-AS1	4.61	3.95	1.59	0.0349	chr4	4q26
RP11-571M6.7	4.72	4.06	1.59	0.0886	chrX	Xq28
RDH10-AS1	6.55	5.88	1.59	0.0476	chr5	
LOC101929516	4.11	3.44	1.59	0.1199	chr14	
RP11-677O4.2	3.46	2.78	1.59	0.0137	chr14	
RP11-555K2.4	4.51	3.86	1.58	0.0552	chr5	
RP5-991C6.4; neyjoy	4.16	3.51	1.58	0.3037	chr7	
LINC01385; RP11-79P5.7	3.95	3.29	1.58	0.0512	chr11	
RP11-619L12.3; flogoy	3.89	3.24	1.58	0.0286	chr5	
CTD-2647E9.3; snoypaw	4.05	3.39	1.58	0.896	chr16	16p13.3
KB-1471A8.1	4.38	3.72	1.58	0.0165	chr12	
CTD-2530H12.7; gleyskeyby	4.39	3.73	1.58	0.0131	chr18	
AC003090.1	4.09	3.42	1.58	0.0624	chr8	
LINC00987	5.47	4.81	1.58	0.0177	chr1	
AC007278.2	4.01	3.34	1.58	0.0099	chr14	
RP11-105N14.3	3.89	3.23	1.58	0.0072	chr1	
RP11-493L12.4; perdeeby	4.05	3.4	1.57	0.0048	chr9	
LOC105374344	4.72	4.08	1.57	0.0118	chr18	
RP11-512C24.3	4.34	3.7	1.57	0.0082	chr2	
LOC101928583	3.87	3.22	1.57	0.0009	chr14	
RP11-417L19.2	5.72	5.08	1.57	0.0404	chrX	Xp22.12
RP4-736H5.3	4.35	3.7	1.57	0.0377	chr12	
RP11-871F6.3	4.02	3.37	1.57	0.2515	chr14	
RP11-120I21.2	6.04	5.39	1.57	0.0222	chr17	
RP11-323F24.4	3.88	3.23	1.57	0.0049	chr17	
RP11-394I13.2	3.73	3.07	1.57	0.03	chr18	
RP11-1148L6.5	6.76	6.1	1.57	0.3395	chr21	21q22.12
LOC101927431; AC006196.1	3.99	3.35	1.56	0.1086	chr8	

LINC01222	3.84	3.2	1.56	0.1677	chr14	
RP11-563P16.1	4.63	3.99	1.56	0.0218	chr1	
RP11-305O4.3	3.88	3.24	1.56	0.021	chr4	
LINC00709	4.03	3.38	1.56	0.0899	chr17	
RP11-91I8.1	4.48	3.84	1.56	0.0436	chr7	
UBA6-AS1	4.42	3.79	1.55	0.0188	chr11	
AC005863.2	4.04	3.41	1.55	0.0284	chr5	
LINC00350	6.65	6.02	1.55	0.007	chr5	
RP11-221N13.4	4.8	4.16	1.55	0.1038	chr12	
AC018737.3; zorstuby	5.24	4.61	1.55	0.243	chr3	
RP11-21M24.2	3.68	3.06	1.54	0.1003	chr17	17q25.1
RP11-285B24.1	4.26	3.63	1.54	0.0131	chr15	15q22.2
LINC01249	4.56	3.94	1.54	0.0058	chr8	
CTD-2532K18.2	5.25	4.62	1.54	0.0115	chr12	
RP6-159A1.3	4.55	3.93	1.54	0.1526	chr15	
RP11-20A20.2	4.19	3.57	1.54	0.7328	chr4	
RP11-497D6.5	5.09	4.46	1.54	0.1633	chrY	Yq11.2
AP001092.4; spymer	4.95	4.32	1.54	0.0333	chr7	
RP4-705F19.1; slerby	4.18	3.57	1.53	0.0126	chr8	
CTD-2055G21.1	5.03	4.42	1.53	0.2279	chr9	9q21.13
LOC101929577; RP11-710F7.3	3.94	3.32	1.53	0.0655	chr6	6q23.3
RP11-557C18.3	4.17	3.55	1.53	0.0236	chr1	
RP11-397O4.1	4.63	4.01	1.53	0.3057	chr10	
RP11-248E9.5	3.86	3.24	1.53	0.0462	chr8	
CTD-2311M21.5	4.18	3.57	1.53	0.0547	chr12	
LINC00640	4.37	3.77	1.52	0.0298	chr12	
AC091493.2	4.52	3.92	1.52	0.2666	chr15	
LINC00693	3.83	3.23	1.52	0.1147	chr7	
CTD-2533K21.4	3.94	3.34	1.52	0.0105	chr13	
RP11-22D3.2	4.74	4.13	1.52	0.0409	chr3	3q23
RP11-399H11.2	6.09	5.48	1.52	0.0682	chr1	
RP11-699A5.2; shysho	3.61	3.01	1.52	0.0055	chr13	13q33.1
GPC6-AS1	4	3.39	1.52	0.0028	chr1	
RP11-328C8.5	5.15	4.54	1.52	0.0982	chr17	
SNORD112; MEG8	4.51	3.92	1.51	0.0974	chrX	
RP11-568A19.1	4.02	3.43	1.51	0.0209	chr1	
RP11-108L7.14	4.91	4.32	1.51	0.0232	chr18	
RP11-315E17.1	4.12	3.53	1.51	0.0297	chr17	17q21.32
CTD-2544H17.2	7.46	6.86	1.51	0.0042	chr2	
ATG10-IT1; RP11-356D23.3	4.28	3.69	1.51	0.0842	chr2	
CTD-2587M2.1	3.89	3.3	1.51	0.6946	chr2	
RP11-128P17.1; soysho	3.88	3.29	1.51	0.1171	chr2	
AC024132.1	4.27	3.68	1.51	0.4355	chr2	
RP11-773H22.4	4.69	4.09	1.51	0.3826	chr8	
RP11-101P17.11	4.03	3.43	1.51	0.1062	chr8	
RP11-863P13.2	3.84	3.24	1.51	0.0251	chr4	
RP11-472G23.10	3.93	3.34	1.51	0.6432	chr3	3q13.2

LINC01497	4.51	3.92	1.5	0.0009	chr19	
LINC01456; RP3-410B11.1	3.98	3.39	1.5	0.0099	chr5	
RP11-76E17.4	3.54	2.96	1.5	0.0602	chr5	
RP11-586K2.1	5.09	4.5	1.5	0.069	chr12	12p13.33
MIR3179-3; MIR3179-1	5.29	4.7	1.5	0.1368	chr13	13q21.2
LINC01455	5.24	4.65	1.5	0.0199	chr6	
LINC00226	14.32	21.56	-1.5	0.7642	chr14	14q32.33
RP11-289F5.1	14.42	21.71	-1.5	0.0186	chr9	
RP11-831F12.2	18.13	27.28	-1.5	0.2224	chr14	
EMG1	83.29	126.24	-1.51	0.1146	chr12	12p13.3
FAM27E2	66.26	100.43	-1.51	0.0512	chr9	9p11.2
LOC399715; RP11-563J2.2	33.36	50.56	-1.51	0.3301	chr10	10p15.1
LOC400794; RP11-280O1.2	26.54	39.95	-1.51	0.434	chr1	1q23.3
AC005532.5; storka	27.28	41.07	-1.51	0.2967	chr7	
NBR2	75.58	113.77	-1.51	0.0472	chr17	17q21
RP5-897D18.1	13.27	19.97	-1.51	0.2523	chr20	
RP5-1184F4.5	35.26	53.08	-1.51	0.0701	chr20	
RP11-6N17.10	13.09	19.70	-1.51	0.1016	chr17	
RP11-73M18.8	28.84	43.41	-1.51	0.1205	chr14	
TCONS_l2_00026969	75.58	113.77	-1.51	0.0809	chr7	
RP11-552C15.1	34.30	51.98	-1.51	0.6361	chr16	
RP11-697E2.11	11.79	17.88	-1.51	0.2268	chr15	
RP11-862P13.1	18.38	27.67	-1.51	0.1384	chr14	
SUCLG2-AS1	36.00	54.57	-1.51	0.0067	chr3	
TBC1D14	53.45	80.45	-1.51	0.1026	chr4	4p16.1
TGFB2; TGFB2-OT1	12.55	19.03	-1.51	0.1023	chr1	1q41
AC093388.3	13.64	20.82	-1.52	0.0282	chr2	
CTB-51J22.1	46.85	71.51	-1.52	0.1239	chr7	
DHRS4-AS1	17.51	26.72	-1.52	0.3267	chr14	14q11.2
AC003984.1	17.63	26.72	-1.52	0.1686	chr7	
MAPKAPK5; ADAM1A	37.27	56.49	-1.52	0.0953	chr12	12q24.13
RP5-867C24.4	274.37	418.77	-1.52	0.0598	chr17	
RP11-120J1.1	31.34	47.50	-1.52	0.048	chr9	
RP11-171I2.1	9.25	14.03	-1.52	0.0275	chr2	
RP11-180C16.1	170.07	257.78	-1.52	0.12	chr7	
RP11-654G14.1	14.62	22.16	-1.52	0.047	chr8	
RP11-689C9.1	10.63	16.22	-1.52	0.0353	chr18	
RP11-715F3.2	58.08	88.65	-1.52	0.0503	chr18	
RP11-932O9.8	25.81	39.40	-1.52	0.222	chr15	
AC003664.1; vyvaw	110.66	168.90	-1.53	0.4107	chr17	
CTD-3064M3.3	10.20	15.67	-1.53	0.1798	chr8	
LA16c-359F1.1	14.72	22.47	-1.53	0.0175	chr16	
RP11-739N10.1	12.73	19.56	-1.53	0.092	chr18	
MIR924HG	27.47	41.93	-1.53	0.1065	chr18	18q12.2
RP11-48G14.3	11.24	17.15	-1.53	0.0545	chr15	
RP11-57A19.5	24.25	37.01	-1.53	0.059	chr16	
RP11-270M14.4	13.36	20.53	-1.53	0.005	chr14	
pywubu	76.11	116.16	-1.53	0.2824	chr17	

AC073218.3	54.95	84.45	-1.54	0.1807	chr2	
blanee; AC012314.20	21.56	33.13	-1.54	0.2946	chr19	
CIRBP-AS1	21.71	33.59	-1.54	0.0365	chr19	19p13.3
LINC01265	10.20	15.67	-1.54	0.5754	chr5	5p13.1
LMLN-AS1	25.28	39.12	-1.54	0.1546	chr3	3q29
LNX1-AS2	15.14	23.26	-1.54	0.0532	chr4	
RP4-782L23.1	12.30	18.90	-1.54	0.2196	chr1	
NOP14-AS1	20.11	30.91	-1.54	0.1066	chr4	4p16.3
RP1-12G14.7	24.25	37.27	-1.54	0.1264	chr6	
RP11-10O22.1	37.27	57.28	-1.54	0.8595	chr3	
RP11-231G15.3	15.35	23.59	-1.54	0.0081	chr5	
RP11-378J18.9	116.16	178.53	-1.54	0.2906	chr1	
RP11-452D21.1	41.64	64.45	-1.54	0.2361	chr14	
RP11-1094M14.14	437826	677565	-1.54	0.2082	chr17	
FAM85A; AC145124.2	17.15	26.54	-1.55	0.0135	chr8	
LINC00623	29.65	45.89	-1.55	0.6022	chr1	1q21.1
LINC01017	31.78	49.18	-1.55	0.0127	chr5	5p15.33
RP3-395M20.8	15.78	24.42	-1.55	0.0276	chr1	1p36.32
RBM23	157.59	243.88	-1.55	0.0534	chr14	14q11.2
RP11-116O18.1	12.30	19.03	-1.55	0.0564	chr18	
RP11-417F21.2	24.42	38.05	-1.55	0.2052	chr2	
RP11-508N22.12	37.53	58.08	-1.55	0.0052	chr10	
RP13-884E18.2	10.27	15.89	-1.55	0.0038	chr4	
BMS1P4	16.00	25.11	-1.56	0.1211	chr10	10q22.2
CTB-167B5.2	16.68	25.99	-1.56	0.1018	chr7	
HIF1A-AS2	12.04	18.77	-1.56	0.002	chr14	
LINC00161	12.47	19.43	-1.56	0.0378	chr21	21q21.3
LINC00998	24.42	38.32	-1.56	0.0768	chr7	7q31.1
AC138035.2	22.94	35.75	-1.56	0.0578	chr5	
RP11-13K12.1	13.74	21.41	-1.56	0.015	chr17	
RP4-591N18.2	19.70	30.91	-1.56	0.0726	chr22	
RP11-47J17.1	26.91	41.93	-1.56	0.103	chr11	
RP11-166N17.3	407.31	634.73	-1.56	0.2829	chr10	
RP11-267D19.1	14.62	22.78	-1.56	0.0801	chr12	
AC092580.4; shawdar	22.94	36.00	-1.57	0.0097	chr2	
CTA-292E10.6; flyter	15.67	24.42	-1.57	0.0699	chr22	
CTD-3131K8.3	105.42	166.57	-1.57	0.0015	chr19	
DPYD-IT1; RP11-359C24.1	14.93	23.43	-1.57	0.2187	chr1	
geedar; RP11-70D24.2	19.29	30.27	-1.57	0.1859	chr16	
ILF3-AS1	25.28	39.67	-1.57	0.0649	chr19	19p13.2
LINC01002	72.50	113.77	-1.57	0.1542	chr19	19p13.3
RP11-345M22.3	47.84	75.06	-1.57	0.2848	chr16	
RP4-669L17.10; spawrubo	27.86	43.71	-1.57	0.6671	chr1	
RP11-20B24.7	23.92	37.53	-1.57	0.095	chr17	
RP11-21K12.3	27.47	43.11	-1.57	0.0962	chr12	
RP11-252A24.5; susuri	25.11	39.40	-1.57	0.5294	chr16	
RP11-295D4.4; steesnor	32.67	51.27	-1.57	0.0673	chr16	
RP11-354P11.3	32.67	50.91	-1.57	0.128	chr17	

RP11-363E7.4	21.26	33.36	-1.57	0.3725	chr9	
RP11-367N14.3	11.96	18.77	-1.57	0.0552	chr4	
RP11-448G15.1	14.12	22.16	-1.57	0.2419	chr4	
RP11-757O6.1	11.16	17.63	-1.57	0.1724	chr18	
SNORD87; SNHG6	1884.54	2957.17	-1.57	0.8615	chr8	8q13.1
TMX2-CTNND1	22.78	35.75	-1.57	0.072	chr11	
AP000487.6	103.97	164.28	-1.58	0.0259	chr11	
CITF22-1A6.3	29.04	45.89	-1.58	0.075	chr22	
DAPK1-IT1; RP11-40C6.3	72.50	114.56	-1.58	0.0663	chr9	
DBNL; MIR6837	17.03	26.72	-1.58	0.7372	chr7	7p13
FAM85B; RP11-556O5.5	79.89	126.24	-1.58	0.0912	chr8	
CTC-308K20.1	34.30	54.19	-1.58	0.0234	chr5	5q35.1
RP11-673E1.1; sitime	19.16	30.27	-1.58	0.1643	chr4	4q31.21
PPIEL	36.00	56.89	-1.58	0.1315	chr1	1p34.3
ZGPATandLIME1	13.74	21.71	-1.58	0.0012	chr20	
RP11-390B4.3	17.15	27.10	-1.58	0.5327	chr10	
RP11-582E3.6	54.19	85.63	-1.58	0.0265	chr12	
BCDIN3D-AS1	25.99	41.07	-1.59	0.044	chr12	12q13.13
CTB-151G24.1	11.55	18.51	-1.59	0.1343	chr19	
HOXB3; HOXB4; MIR10A	20.68	32.90	-1.59	0.1257	chr17	17q21.3
KLF3-AS1	14.52	23.10	-1.59	0.3616	chr4	4p14
RP11-10E18.7	10.56	16.80	-1.59	0.0128	chr13	
RP11-73M18.7	37.79	60.13	-1.59	0.0522	chr14	
RP11-234G16.5	16.68	26.54	-1.59	0.0415	chr10	
RP11-702F3.1	16.22	25.81	-1.59	0.1349	chr11	
LINC00597	19.29	30.91	-1.6	0.1536	chr15	15q23-q24
RP11-394G3.2	15.56	24.93	-1.6	0.0544	chr6	
RP11-525K10.3; tuzybu	36.76	58.89	-1.6	0.3171	chr16	16q23.2
MYADM	42.22	68.12	-1.6	0.2541	chr19	19q13.42
PRR34-AS1	225.97	362.04	-1.6	0.5907	chr22	22q13.31
RP5-1021I20.2	41.64	66.72	-1.6	0.1912	chr14	
AD001527.7	515.56	831.75	-1.61	0.5433	chr19	
FAM41C	119.43	192.67	-1.61	0.22	chr1	1p36.33
FAM95B1	34.30	54.95	-1.61	0.0109	chr9	9p12
FLJ36777; RP11-367J11.3	16.80	27.10	-1.61	0.0203	chr4	
KLHL6-AS1	40.79	65.34	-1.61	0.7783	chr3	3q27.1
OR2A9P; OR2A20P	23.10	37.27	-1.61	0.1421	chr7	7q35
RP4-657D16.6	12.55	20.11	-1.61	0.0658	chr1	
RP5-1021I20.8	28.84	46.53	-1.61	0.0133	chr14	
RP11-542M13.2	50.91	81.57	-1.61	0.245	chr16	
C21orf91; C21orf91-OT1	95.01	154.34	-1.62	0.0211	chr21	21q21.1
GS1-124K5.3	14.83	24.08	-1.62	0.001	chr7	
HTR7P1	10.06	16.34	-1.62	0.0278	chr12	12p13.1
LINC01531	12.13	19.70	-1.62	0.0282	chr19	19q13.12
RP11-356J5.13	25.46	41.36	-1.62	0.0396	chr11	11q23.1
RP11-6J21.2	11.63	18.77	-1.62	0.2937	chr1	
SNORA44; SNORA6	57.68	93.70	-1.62	0.3087	chr1	1p35.3
AC064871.3	15.67	25.63	-1.63	0.0219	chr2	

GTF2IP20	11.88	19.43	-1.63	0.072	chr1	1q42.11
LOC100506076; yumomo	28.05	45.57	-1.63	0.5918	chr2	
RP3-453C12.14	43.71	71.01	-1.63	0.1288	chr20	
RP11-283G6.5; sheestaw	24.25	39.67	-1.63	0.3935	chr12	
AC006041.1; starcharby	13.27	21.86	-1.64	0.0728	chr7	
RP11-734K23.9; romomo	67.65	110.66	-1.64	0.4403	chr2	2q11.2
RP4-633I8.4	22.01	36.25	-1.64	0.315	chr1	
RP11-53B2.2	59.30	97.68	-1.64	0.2446	chr18	
RP11-59E19.4	60.97	100.43	-1.64	0.1458	chr3	
RP11-166O4.6	25.11	41.07	-1.64	0.161	chr7	
RP11-325K4.2	16.56	27.10	-1.64	0.8215	chr16	
RP11-519C12.1	14.62	23.92	-1.64	0.0975	chr15	
RP1-267L14.3	27.28	44.94	-1.65	0.0648	chr12	
RP11-291I6.2	83.87	138.14	-1.65	0.0383	chr13	
ITCH-AS1; RP3-468O1.4	1360.57	2256.70	-1.66	0.1349	chr20	
LOC100289230	51.98	86.82	-1.66	0.0493	chr5	5q21.1
PCAT19	11.63	19.29	-1.66	0.0386	chr19	
RP11-50I19.2	10.27	17.03	-1.66	0.0608	chr12	
AP000769.7	10.56	17.51	-1.67	0.0427	chr11	
RP11-626P14.1	11.55	19.29	-1.67	0.1613	chr14	
RP11-707P20.1	18.51	30.91	-1.67	0.01	chrX	Xp22.32
OIP5-AS1	74.54	124.50	-1.67	0.0659	chr15	15q15.1
RP1-168P16.2	16.45	27.67	-1.67	0.0293	chr16	
RP11-415J8.7	9.78	16.34	-1.67	0.0294	chr1	
RP11-661C3.2	22.63	38.05	-1.67	0.035	chr17	
GAS5; SNORD74	53.08	88.65	-1.68	0.008	chr1	1q25.1
LOC100129034	16.91	28.44	-1.68	0.642	chr9	9q33.3
RP11-800A3.2; lerglawbu	33.59	56.49	-1.68	0.021	chr11	
AF001550.7	17.27	29.24	-1.69	0.1386	chr16	
bP-21264C1.2	70.03	118.60	-1.69	0.2486	chr21	
MALT1	11.63	19.70	-1.69	0.5102	chr18	18q21
RP3-406A7.7	11.79	19.97	-1.69	0.0735	chr6	
RP11-865I6.2	20.25	34.30	-1.69	0.0694	chr8	
LOC286437	87.43	148.06	-1.7	0.0329	chrX	Xq22.2
PIGP	15.56	26.54	-1.7	0.0359	chr21	21q22.2
PRKCQ-AS1	37.01	63.12	-1.7	0.2087	chr10	10p14
RP11-88I18.2	28.84	49.18	-1.7	0.4265	chr9	
RP11-861E21.1; sparraw	65.80	112.21	-1.7	0.2255	chr18	
SNORA59B; SNORA59A	20.82	35.51	-1.7	0.0283	chr17	17p11.2
AC016682.1; larly	19.16	32.90	-1.71	0.0382	chr2	
RP11-760M1.3; ternor	13.09	22.47	-1.71	0.063	chr13	
RP11-136K14.2	8.75	14.93	-1.71	0.0203	chr6	
RP11-211G23.1	18.13	31.12	-1.71	0.0568	chr11	
CTD-2383I20.1	14.93	25.63	-1.72	0.0556	chr5	
SCARNA10	19.29	33.36	-1.72	0.0299	chr12	12p13.31
Y_RNA; RP11-48B3.5	32.22	55.33	-1.72	0.0363	chr8	
RP11-160E2.21	191.34	330.84	-1.73	0.0825	chr17	
RP11-457M11.7	22.63	39.12	-1.73	0.016	chr6	

SOD2	33.13	57.28	-1.73	0.733	chr6	6q25.3
CLUHP3	15.35	26.72	-1.74	0.0018	chr16	16p11.2
CTC-429P9.2	41.64	72.50	-1.74	0.335	chr19	
RP11-70D24.3	46.53	81.01	-1.74	0.6103	chr16	
VTRNA2-1	25.46	44.32	-1.74	0.1828	chr5	5q31.1
GUCY1A3	46.53	81.01	-1.75	0.0019	chr4	4q32.1
LLNLR-246C6.1	12.82	22.32	-1.75	0.0254	chr19	
RP1-60O19.1; LOC553137	44.94	78.25	-1.75	0.1113	chr6	
PRNCR1	10.85	19.03	-1.75	0.0047	chr8	8q24.21
RP11-50B3.4	19.70	34.54	-1.75	0.4826	chr11	
RP11-57H14.4	30.70	54.19	-1.76	0.0392	chr10	10q25.2
RP1-140K8.5	16.22	28.44	-1.76	0.0047	chr6	
RP13-317D12.3	1398.83	2452.44	-1.76	0.0486	chr11	
AC006116.19	13.64	24.42	-1.78	0.0968	chr19	
AJ239322.1; ramomo	94.35	167.73	-1.78	0.008	chr2	
RP11-17E13.2	29.24	51.98	-1.78	0.0186	chr1	
U91328.22	54.19	96.34	-1.78	0.3063	chr6	
LINC01530	14.83	26.54	-1.79	0.0285	chr19	19q13.41
RP11-90B9.3	13.00	23.26	-1.79	0.0934	chr15	
RP11-180N14.1	11.71	21.11	-1.79	0.0198	chr3	
RP11-817I4.1	23.26	41.64	-1.79	0.1149	chr12	
ASH1L-IT1; RP11-21N7.6	75.06	135.30	-1.8	0.2782	chr1	
RP11-138H8.2	18.25	32.90	-1.8	0.0406	chr15	
PLAGL1; HYMAI	70.52	127.12	-1.81	0.7296	chr6	6q24-q25
RP11-150O12.3	21.56	39.12	-1.81	0.1412	chr8	
RP11-563J2.3	22.94	41.36	-1.81	0.0027	chr10	
FAM223A; FAM223B	22.01	40.22	-1.82	0.088	chrX	
AC093375.1; geedo	16.00	29.04	-1.82	0.0652	chr2	2q24.1
RP11-484O2.1; slawblerby	22.94	41.64	-1.82	0.138	chr4	
DSCR8	13.09	24.08	-1.83	0.034	chr21	21q22.2
RP3-405J10.3	82.71	151.17	-1.83	0.1511	chr12	
RP11-278C7.4	33.82	61.82	-1.83	0.2249	chr12	
AC079630.2; snynaw	14.32	26.35	-1.84	0.0046	chr12	
RP11-23F23.2	8.75	16.11	-1.84	0.024	chr11	
RP11-15A1.8	12.64	23.26	-1.84	0.0173	chr19	
RP11-350N15.6	17.75	32.67	-1.84	0.2224	chr8	
TIMP3	17.15	31.56	-1.84	0.0081	chr22	22q12.3
CTC-513N18.6	47.18	86.82	-1.85	0.0335	chr19	
RP11-157H4.1	37.01	68.59	-1.85	0.0077	chr13	
RP11-196G11.5	31.78	58.89	-1.85	0.0025	chr16	
RP11-128N14.5	37.01	69.07	-1.86	0.103	chr13	
RP11-762I7.4	121.10	224.41	-1.86	0.0539	chr12	
NRSN2-AS1	24.42	45.57	-1.87	0.0011	chr20	
RP3-445N2.1	9.85	18.38	-1.87	0.0784	chr6	
RP11-529E10.7	18.51	34.54	-1.87	0.0631	chr4	
RP11-83M16.5; plostarbu	20.11	37.79	-1.88	0.1424	chr5	
CTD-2538G9.6	572.05	1082.39	-1.89	0.0204	chr19	
PCF11	39.67	75.06	-1.89	0.0264	chr11	11q13

RP11-5L12.1	122.79	232.32	-1.89	0.5111	chr11	
RP11-90C4.3; shoboy	14.12	26.54	-1.89	0.2573	chr1	
AC139100.3	10.41	19.84	-1.9	0.002	chr18	
RP11-1149O23.2; jargor	10.13	19.16	-1.9	0.0766	chr8	
AC107072.2	29.24	55.72	-1.91	0.0066	chr4	
ACAP2; ACAP2-IT1;	20.82	39.67	-1.91	0.016	chr3	
MIR214; DNM3OS	78.25	149.09	-1.91	0.1332	chr1	1q24.3
ATXN10	148.06	284.05	-1.92	0.6621	chr22	22q13.31
DENND5B	20.97	40.22	-1.92	0.0414	chr12	12p11.21
LINC01127	33.82	64.45	-1.92	0.1649	chr2	2q12.1
byrer; RP11-182J1.13	79.34	152.22	-1.92	0.0029	chr15	15q25.2
RP11-805J14.3; kleytobo	430.54	826.00	-1.92	0.1028	chr11	
AKAP10	32.67	63.12	-1.93	0.0203	chr17	17p11.1
RP3-426I6.5	81.57	157.59	-1.93	0.0242	chr1	
RP11-123M21.1	11.16	21.71	-1.94	0.1057	chr12	
RP11-750H9.7	256.00	494.56	-1.94	0.1148	chr11	
ASAP1-IT1	24.93	48.50	-1.95	0.0255	chr8	8q24.21
LOC645513	84.45	164.28	-1.95	0.0425	chr4	4q26
MIR4435-2HG	119.43	233.94	-1.95	0.0868	chr2	2q13
PPP1R14B	357.05	694.58	-1.95	0.2233	chr11	11q13
DHRSX-IT1; RP13-858C7.1	27.47	53.82	-1.96	0.0022	chrX	
RP11-807H17.1	33.13	65.34	-1.96	0.0474	chr7	
SCARNA9	39.95	78.25	-1.96	0.0413	chr11	11q21
CTD-2027I19.3	40.50	79.89	-1.97	0.054	chr19	
RP11-11N9.4	33.82	66.72	-1.97	0.0134	chr8	
RP11-7M10.2; skawploy	544.96	1074.91	-1.98	0.1423	chr15	
RP11-475I24.3	26.72	53.45	-2.01	0.0022	chr9	9p11.1
HOTAIRM1	1305.15	2646.74	-2.02	0.2099	chr7	7p15.2
RP11-74J13.8	13.36	27.10	-2.02	0.0101	chr13	13q14.11
RP11-109E12.1	45.89	92.41	-2.02	0.0346	chr2	
ZFP91-CNTF	26.72	54.19	-2.02	0.0172	chr11	11q12.2
H19; MIR675; AC004556.2	28.84	58.49	-2.03	0.4446	chr11	
LINC00152	826.00	1675.06	-2.03	0.1674	chr2	2p11.2
RP11-66H6.4	13.18	26.72	-2.03	0.0076	chr16	
CTC-471J1.2	17.88	36.76	-2.05	0.0177	chr19	
ISM1-AS1	20.39	42.52	-2.08	0.9543	chr20	
RP11-316M21.7	38.05	79.34	-2.09	0.0148	chr10	
WDFY3-AS2	25.81	54.19	-2.11	0.1379	chr4	4q21.3
AC137934.1	27.47	58.49	-2.12	0.3861	chr16	
AP000473.6	47.18	99.73	-2.12	0.0103	chr21	
KB-1507C5.3	1074.91	2304.12	-2.14	0.2064	chr8	
LOC102724034	56.49	121.94	-2.16	0.0009	chr15	15q25.2
LOC103021295	199.47	430.54	-2.16	0.0575	chr1	
RP11-3P17.5	130.69	288.01	-2.2	0.0078	chr3	
RP11-391M1.4	97.68	215.27	-2.2	0.2663	chr3	
KCNQ1OT1	73.52	162.02	-2.21	0.1681	chr11	11p15
RP11-214K3.22	24.25	53.45	-2.21	0.0739	chr12	
USP9X	38.05	85.63	-2.24	0.0318	chrX	Xp11.4

RP11-118E18.2; josley	12.04	27.10	-2.26	0.0003	chr17	17q22
GLIDR; RP11-211N8.2	22.01	50.21	-2.27	0.0013	chr9	
LINC00622	50.56	115.36	-2.28	0.0743	chr1	1p12
RP1-257A7.5	34.78	79.34	-2.28	0.0061	chr6	
dorjuby; RP11-864G5.3	49.87	114.56	-2.29	0.1441	chr11	
RP11-1046B16.3	70.03	162.02	-2.32	0.0383	chr14	
LINC01158	401.71	935.76	-2.33	0.2082	chr2	2q12
RP11-28F1.2	23.92	56.49	-2.36	0.0019	chr18	
RP11-326G21.1	147.03	349.71	-2.37	0.0188	chr1	
NPIPB5	335.46	803.41	-2.39	0.0531	chr16	16p12.2
RP11-356M20.3	137.19	326.29	-2.39	0.0268	chr15	
RP11-539E19.2; sneemoyby	47.18	112.99	-2.39	0.0643	chr10	
blara; RP11-254F19.2	32.00	76.64	-2.4	0.0384	chr16	
DIP2A-IT1	43.11	106.89	-2.48	0.0379	chr21	21q22.3
CTC-546K23.1; geeskorbu	385.34	955.43	-2.48	0.1477	chr5	5q23.1
LOC100190986	23.59	58.89	-2.49	0.082	chr16	16p12.2
RP11-888D10.4	16.11	40.22	-2.5	0.0072	chr18	
SNORD50A; SNHG5;	814.63	2033.85	-2.5	0.1432	chr6	6q14.3
RNASEK; C17orf49	1478.58	3717.20	-2.51	0.037	chr17	17p13.1
RP11-264B14.2	75.06	194.01	-2.59	0.0342	chr17	
RP11-530C5.4	19.97	51.63	-2.59	0.0021	chr12	
pleyglorby	93.05	249.00	-2.67	0.1296	chr7	7p15.1
RP11-359J14.2	29.86	79.89	-2.68	0.0034	chr12	
SCIN	19.43	52.71	-2.71	0.0105	chr7	7p21.3
CTC-360G5.9	145.01	401.71	-2.78	0.028	chr19	
GLIDR; RP11-262H14.4	216.77	625.99	-2.89	0.0072	chr9	9q13
SNX2	512.00	1562.89	-3.06	0.1923	chr5	5q23
RP11-111F5.3	30.06	95.01	-3.14	0.0065	chr9	
ANKRD10-IT1	20.68	64.89	-3.16	0.006	chr13	
PCBP2; PCBP2-OT1	121.10	439.59	-3.62	0.0364	chr12	12q13.13
SLC38A1	471.14	1871.53	-3.96	0.0595	chr12	12q13.11
RP11-410E4.1	17.03	76.64	-4.48	0.0066	chr2	
RP11-140H17.2	14.52	67.65	-4.64	0.0022	chr16	
AC002044.4	1314.23	6295.04	-4.78	0.0239	chr16	
RPL37	3147.52	15181.2	-4.85	0.0725	chr5	5p13
EPB41L4A-AS1	20.68	123.64	-5.96	0.0029	chr5	5q22.2
CTC-444N24.11	390.72	2368.90	-6.05	0.0032	chr19	
RP11-105C19.1	222.86	1438.15	-6.44	0.0006	chr16	
RP11-138H8.8	69.55	621.67	-8.93	0.0017	chr15	

Supplementary Table S5. List of snoRNAs differentially expressed between NR and R.

snoRNA	NR intensity	R intensity	Fold Change	P-val	Chromosome	Start	Stop
ACA40	292.04	69.55	4.22	0.0335	chr11	93468276	93468402
ENSG00000207187	95.67	27.47	3.48	0.0202	chr2	30410300	30410432
ACA10	95.67	27.47	3.48	0.0202	chr16	2012335	2012467
U17a	80.45	24.93	3.22	0.0038	chr1	28833877	28834083
U17a	436.55	143.01	3.06	0.005	chr1	28833877	28834083
ENSG00000207130	270.60	103.97	2.61	**0.0266**	chr3	128433414	128433548
ENSG00000206903	270.60	103.97	2.61	0.0266	chr15	65577799	65577929
ACA24	270.60	103.97	2.61	0.0266	chr4	119200345	119200475
ACA4	47.18	18.25	2.58	0.0087	chr3	186505402	186505538
U92	147.03	60.55	2.43	0.0234	chr9	19063654	19063784
U17b	2778.33	1192.69	2.34	0.003	chr1	28835070	28835274
U18C	102.54	44.63	2.3	0.0325	chr15	66793590	66793656
ACA16	12.55	5.58	2.25	0.0043	chr1	28907432	28907565
U19	75.06	33.59	2.24	0.0053	chr5	138614469	138614668
SNORA84	118.60	54.57	2.18	0.0219	chr9	95054743	95054875
ENSG00000239026	6.23	2.89	2.17	0.0097	chr5	174877819	174877921
ACA55	182.28	84.45	2.17	0.0061	chr1	40033046	40033182
ENSG00000252505	18.64	8.69	2.14	0.0024	chr8	33397558	33397655
ACA46	25.46	12.30	2.07	0.0078	chr16	58582403	58582537
ACA11	33.82	16.56	2.04	0.0195	chr4	1976363	1976487
U50	1341.84	670.92	2.01	0.007	chr6	86387012	86387086
U42B	33.36	16.80	1.98	0.0209	chr17	27047568	27047634
U71d	233.94	119.43	1.96	0.0142	chr20	37062505	37062642
ACA24	23.43	12.21	1.92	0.0368	chr4	119200345	119200475
U42B	7.21	3.84	1.88	0.0178	chr17	27047568	27047634
U83A	306.55	165.42	1.86	0.035	chr22	39711218	39711312
U69	42.52	23.43	1.81	0.0417	chrX	118921316	118921447
ENSG00000252765	4.38	2.46	1.78	0.0019	chr1	101598709	101598895
ENSG00000252505	12.91	7.26	1.78	0.0148	chr8	33397558	33397655
ACA16	67.65	38.05	1.77	0.0005	chr1	28907432	28907565
U90	73.52	41.64	1.76	0.0196	chr3	160232695	160233024
ACA31	54.57	30.91	1.76	0.0419	chr13	45911615	45911744
U37	1082.39	613.11	1.76	0.0464	chr19	3982505	3982570
U16	155.42	88.65	1.75	0.0196	chr15	66795149	66795249
U78	634.73	364.56	1.74	0.0163	chr1	173834760	173834824
HBI-100	39.95	23.10	1.72	0.0141	chr1	175937533	175937676
U79	382.68	229.13	1.67	0.0328	chr1	173834486	173834570
HBII-276	445.72	270.60	1.64	0.0058	chr8	67834709	67834784

U71d	143.01	88.65	1.61	0.0401	chr20	37062505	37062642
ACA45	8.28	5.31	1.55	0.0221	chr15	83424697	83424823
ACA61	380.04	245.57	1.55	0.0121	chr1	28906276	28906405
ENSG00000252765	4.11	2.68	1.54	0.0048	chr1	101598709	101598895
ENSG00000212567	5.46	3.61	1.52	0.0022	chr5	40790179	40790306
ACA58	8.22	5.46	1.51	0.0075	chr3	131197941	131198077
ENSG00000238343	5.78	3.84	1.5	0.0329	chr16	68347142	68347243
ENSG00000252409	3.61	5.46	-1.52	0.0054	chr3	41729360	41729525
ENSG00000238843	3.29	4.99	-1.52	0.0195	chr1	156499122	156499221
ENSG00000212191	4.11	6.28	-1.53	0.0252	chr4	179607040	179607110
ENSG00000221300	14.93	24.42	-1.63	0.0408	chr2	42667517	42667576

Supplementary Table S6. List of genes differentially expressed between NR and R.

Gene Symbol	NR intensity	R intensity	Fold Change	*P*-val
EDC3	6.1	4.62	2.79	0.0064
DOCK4	8	6.67	2.52	0.0046
MGAT2P1	5	3.69	2.48	0.0011
HFM1	4.88	3.62	2.39	0.0196
OR56A4	4.82	3.56	2.39	0.0094
MTND5P5	5.83	4.58	2.38	0.0216
OR8D1	5.08	3.93	2.22	0.015
OR10AK1P	4.32	3.21	2.17	0.01
MIR1297	5.38	4.28	2.14	0.008
CENPI	5.89	4.81	2.12	0.0138
SNORD3B-2	7.73	6.65	2.11	0.0371
MIR92A2	4.95	3.89	2.09	0.0355
LYPD1	4.53	3.47	2.08	0.013
TRIM60P3Y	5.09	4.05	2.06	0.0104
MTND6P10	8.81	7.82	1.99	0.0093
OR6C74	4.8	3.8	1.99	0.0143
B3GALNT1P1	5.15	4.16	1.98	0.0368
MTCO1P27	4.07	3.09	1.98	0.0078
UCHL1	4.83	3.84	1.98	0.004
OR13G1	4.19	3.23	1.95	0.0045
SLPI	5.87	4.9	1.95	0.0293
SNORD115-28	5.01	4.06	1.93	0.0394
SPANXC; SPANXD	5.77	4.82	1.93	0.0017
TRAJ2	6.34	5.4	1.93	0.0046
ACTR3BP5	4.93	3.99	1.92	0.0141
MEIOC	4.61	3.67	1.92	0.0277
MIR4483	5.24	4.3	1.92	0.0039
TATDN1P1	5.04	4.1	1.92	0.0302
MIR3977	4.62	3.69	1.91	0.0203
HSPA8P19	4.09	3.16	1.9	0.0056
MTCO2P25	4.25	3.33	1.9	0.0333
MAK	4.96	4.04	1.89	0.0163
MIR1302-1	4.83	3.92	1.88	0.0061
MIRLET7E	5.33	4.42	1.88	0.006
MTCYBP23	3.79	2.88	1.88	0.0114
MTATP6P16	4.23	3.33	1.87	0.0316
MTND5P34	4.28	3.38	1.87	0.0345
SETD4	4.18	3.28	1.86	0.0057
HMBOX1	4.6	3.71	1.85	0.0167
TAF6	5.06	4.17	1.85	0.0034
LMAN2L	4.73	3.85	1.83	0.0111

RNU6-650P	7.88	7.01	1.83	0.0099
APOH	3.91	3.05	1.82	0.0046
EPYC	4.1	3.24	1.82	0.0028
IAPP	4.02	3.15	1.82	0.027
NT5DC3	6.28	5.42	1.82	0.0168
IGHV4-59	4.82	3.97	1.81	0.0279
C8orf22	4.55	3.7	1.8	0.0301
COL16A1	5.27	4.43	1.78	0.035
MTND2P3	4.11	3.28	1.78	0.0124
NAA15	6.67	5.84	1.78	0.0142
THEGL	4.62	3.79	1.78	0.0104
FSD2	4.58	3.75	1.77	0.0005
GXYLT1P3	4.58	3.76	1.77	0.0008
OR8G1	4.54	3.73	1.76	0.036
CYB561D2	4.36	3.55	1.75	0.0347
IGHV3-48	4.12	3.32	1.75	0.0429
IGHV3OR16-11	6.06	5.25	1.75	0.0084
MIR7973-2	5.99	5.18	1.75	0.0011
MTRNR2L5	6.01	5.2	1.75	0.0172
KRTAP8-2P	5.45	4.65	1.74	0.0174
MTND3P9	5.01	4.22	1.74	0.0199
OFD1P6Y	4.41	3.62	1.74	0.0039
OR13H1	4.94	4.14	1.74	0.0207
SERPINB8	4.47	3.67	1.74	0.0111
DDX43	4.18	3.39	1.73	0.0199
ENKUR	5.17	4.38	1.73	0.0114
OR5T2	4.09	3.3	1.73	0.0283
RFPL4B	4.29	3.5	1.73	0.0347
SLC23A3	4.51	3.72	1.73	0.032
TNKS	4.13	3.34	1.73	0.0287
UBE2D3P3	4.15	3.36	1.73	0.0115
ASNS	7.14	6.36	1.72	0.0196
C2orf80	4.35	3.57	1.72	0.0116
SGOL2	4.04	3.26	1.72	0.0451
FBXO47	4.5	3.72	1.71	0.0166
GAPDHP36	4.06	3.29	1.71	0.0039
PRELID3BP11	4.5	3.73	1.71	0.0007
AC010655.1	4.74	3.97	1.7	0.0348
ARMC3	5.3	4.54	1.7	0.0367
LINC00879	4.68	3.91	1.7	0.0145
MIA2	4.65	3.89	1.7	0.0476
BPIFB4	3.43	2.68	1.69	0.0039
CADPS	4.01	3.25	1.69	0.0017
CDKN2AIP	3.88	3.12	1.69	0.0165
CLLU1	4.1	3.34	1.69	0.0091
LAX1	4.54	3.78	1.69	0.0013

PIH1D3	5.77	5.02	1.69	0.0148
PPM1K	5.83	5.08	1.69	0.0268
SERPINB4	4.26	3.5	1.69	0.0028
ARHGEF26	4.66	3.9	1.68	0.0126
BNIP3P23	4.14	3.39	1.68	0.0221
HMGB4	4	3.26	1.68	0.016
MIR19B2	4.09	3.34	1.68	0.0406
RPP14	4.15	3.4	1.68	0.0015
UBDP1	5.28	4.53	1.68	0.0438
ARL6IP5	6.8	6.06	1.67	0.0055
GREM1	4.89	4.16	1.67	0.0363
MTND6P6	4.44	3.7	1.67	0.0069
NLRP2	4.29	3.55	1.67	0.0036
MTND2P4	4.34	3.61	1.66	0.0336
NXPH2	8.59	7.86	1.66	0.032
OR2L13	3.91	3.18	1.66	0.0303
PCMTD1	4.06	3.33	1.66	0.0228
TMEM241	3.75	3.02	1.66	0.0344
USP47	4.47	3.74	1.66	0.0486
ATP12A	4.25	3.52	1.65	0.0118
BNIP3P37	6.57	5.85	1.65	0.0276
CHIC1	4.59	3.86	1.65	0.0263
KLK13	3.93	3.21	1.65	0.0028
APOC4	5.12	4.4	1.64	0.0091
OPALIN	4.42	3.7	1.64	0.0109
RPS3AP17	4.3	3.58	1.64	0.0043
KRTAP13-3	4.35	3.65	1.62	0.0125
LOC650226	5.02	4.33	1.62	0.0302
MIR505	6.02	5.33	1.62	0.0455
RPS6P21	3.95	3.26	1.62	0.0058
SMOC1	4.98	4.28	1.62	0.0015
VCP	5.83	5.13	1.62	0.0076
CCDC69	5.24	4.55	1.61	0.025
MTND5P19	4.47	3.78	1.61	0.032
ZFP57	3.6	2.92	1.61	0.0032
MTCO3P23	5.29	4.61	1.6	0.0415
RBMX2P4	4.25	3.57	1.6	0.0382
SNRPGP11	8.3	7.63	1.6	0.0135
TMSB4XP3	4.42	3.74	1.6	0.0256
ZNF20	4.57	3.9	1.6	0.0158
AKR1D1	4.7	4.03	1.59	0.0028
CERKL	3.9	3.24	1.59	0.0078
DEFB119	3.59	2.92	1.59	0.0273
HNRNPCL3	5.37	4.71	1.59	0.0141
KBTBD4	6.24	5.57	1.59	0.0471
RAC1P1	4.24	3.57	1.59	0.0203

RPS20P32	6.46	5.79	1.59	0.0185
AKAP6	4.1	3.44	1.58	0.0319
CD47	5.15	4.49	1.58	0.0313
FER1L4	4.9	4.24	1.58	0.0119
KIF20A	5.07	4.41	1.58	0.0437
MAS1	5.96	5.3	1.58	0.0068
MIR496	4.59	3.93	1.58	0.0185
OR2H2	3.88	3.22	1.58	0.0052
RGPD2; RGPD1	4.22	3.57	1.58	0.0069
RNU1-97P	4.75	4.09	1.58	0.0083
SLC12A8	4.91	4.25	1.58	0.0071
SNRPD2P2	4.07	3.4	1.58	0.0305
SPANXD	5.58	4.92	1.58	0.0063
CYCSP52	4.98	4.33	1.57	0.043
MIR3179-2	5.38	4.73	1.57	0.0221
OR10K2	4.55	3.9	1.57	0.0124
POTEI	4.71	4.07	1.57	0.0086
RNU6-678P	4.98	4.33	1.57	0.0301
SLIT2	5.4	4.74	1.57	0.0293
SPACA1	4.37	3.72	1.57	0.0229
TAAR9	3.82	3.17	1.57	0.0304
CTCFL	4.98	4.35	1.56	0.0096
EIF4A1P13	4.86	4.22	1.56	0.0168
MUC17	3.99	3.35	1.56	0.0439
NPFFR1	4.33	3.69	1.56	0.0179
OR2AE1	5.14	4.5	1.56	0.0007
RPL35AP15	4.2	3.56	1.56	0.0271
SNORD114-24	3.74	3.1	1.56	0.0439
CENPF	4.52	3.89	1.55	0.0067
DCLRE1C	4.15	3.52	1.55	0.0498
ERICH3	3.68	3.04	1.55	0.0321
IGHV3OR16-8	4.7	4.08	1.55	0.0414
INTS6P1	4.64	4	1.55	0.0442
MTAPP1	4.11	3.48	1.55	0.0148
NDUFS5P5	4.85	4.22	1.55	0.0032
PMP2	3.8	3.17	1.55	0.0074
PPP1R1C	6.19	5.56	1.55	0.0213
PPP1R42	3.87	3.23	1.55	0.0395
SLC27A6	4.01	3.37	1.55	0.0143
SNTB1	4.1	3.47	1.55	0.0271
STRADBP1	3.88	3.25	1.55	0.0018
ZBTB7C	4.3	3.66	1.55	0.0358
FAM60DP	3.75	3.12	1.54	0.0447
GLT8D2	3.73	3.11	1.54	0.0134
MTND2P14	4.17	3.55	1.54	0.0273
OR51A7	3.93	3.31	1.54	0.0194

SMCP	3.68	3.06	1.54	0.0063
TACR3	3.49	2.87	1.54	0.0295
ANKRD34C	3.69	3.07	1.53	0.0191
C3orf14	4.28	3.66	1.53	0.0044
CLASP1	6.18	5.57	1.53	0.045
HOXA1	4.31	3.69	1.53	0.0335
IGLV3-21	5.28	4.67	1.53	0.0464
IL1RN	5.11	4.5	1.53	0.0437
OPHN1	4.35	3.74	1.53	0.0253
SPATA2P1	4.76	4.15	1.53	0.0313
TGFBR3L	4.62	4	1.53	0.0045
TMEM117	3.71	3.1	1.53	0.002
TRBV5-1	3.79	3.18	1.53	0.016
TTPA	3.83	3.22	1.53	0.0147
ZNF101	5.2	4.58	1.53	0.0167
ZNF425	4.85	4.24	1.53	0.0014
ACOT12	3.71	3.1	1.52	0.0141
BDKRB1	3.96	3.36	1.52	0.0078
FTH1P20	4.31	3.71	1.52	0.0367
HNRNPDP2	5.56	4.96	1.52	0.0173
LINC00640	4.37	3.77	1.52	0.0463
OFD1P9Y	3.91	3.3	1.52	0.0431
PRKAR2A	4.46	3.86	1.52	0.0435
RPL9P33	4.81	4.2	1.52	0.0078
SRSF10P1	4.38	3.77	1.52	0.0476
TNNC1	4.84	4.24	1.52	0.0297
TRAV4	3.74	3.13	1.52	0.0062
DDN	3.5	2.91	1.51	0.0385
GAMTP2	4.37	3.77	1.51	0.0371
GVINP2	5.02	4.43	1.51	0.0259
IL31	3.68	3.09	1.51	0.0406
MIR4272	4.48	3.89	1.51	0.039
MRPL45P1	3.65	3.06	1.51	0.033
NDUFA12P1	4.78	4.19	1.51	0.0161
NOXRED1	4.39	3.8	1.51	0.025
OFD1P8Y	3.62	3.02	1.51	0.0063
PLEKHB2	4.35	3.75	1.51	0.004
PRRG2	4.83	4.23	1.51	0.0389
PRYP6	4.85	4.26	1.51	0.0308
RPS6P8	6.69	6.09	1.51	0.0099
SLC24A4	3.85	3.26	1.51	0.0031
SNORD112; MEG8	4.51	3.92	1.51	0.0414
SNRPGP20	7.51	6.92	1.51	0.0468
VN1R51P	3.96	3.36	1.51	0.0252
ZSCAN10	5.93	5.34	1.51	0.0237
ART2BP	4.52	3.93	1.5	0.0022

DBX2	5.15	4.56	1.5	0.0084
FCRL4	4.2	3.62	1.5	0.0108
IGHVIII-67-3	3.72	3.13	1.5	0.0421
IGLV3-27	4.89	4.31	1.5	0.0071
KRTAP21-2	4.02	3.43	1.5	0.0137
MIR3179-3	5.29	4.7	1.5	0.0132
RGS22	4.16	3.57	1.5	0.0292
USP26	5.04	4.45	1.5	0.0055
ZSWIM2	4.39	3.8	1.5	0.016
GNS	6.55	7.14	-1.5	0.0279
KIAA0319L	5.84	6.42	-1.5	0.0323
MINA	3.78	4.37	-1.5	0.0219
MRPS2	4.25	4.84	-1.5	0.0096
PHACTR2	4.68	5.27	-1.5	0.0416
ATG5	4.08	4.68	-1.51	0.0459
EIF1P6	3.97	4.56	-1.51	0.0036
HERPUD2	3.76	4.36	-1.51	0.0288
LIMA1	6.41	7.01	-1.51	0.0118
MIR3687-2	6.85	7.44	-1.51	0.0187
PRADC1	4.72	5.31	-1.51	0.0091
RHOBTB2	4.97	5.56	-1.51	0.043
TRMT10B	3.82	4.41	-1.51	0.0304
ACAA2	4.82	5.43	-1.52	0.0136
CCDC90B	4.98	5.58	-1.52	0.0496
CD93	4.17	4.77	-1.52	0.0234
CNTNAP3P2	5.34	5.94	-1.52	0.0103
HMGB1P20	4.13	4.73	-1.52	0.0318
NADSYN1	4.09	4.7	-1.52	0.0478
NDUFA13	4.82	5.42	-1.52	0.0182
NUSAP1	3.46	4.06	-1.52	0.0185
OGDH	6.28	6.88	-1.52	0.007
PLA2G4C	3.62	4.22	-1.52	0.0159
SLC44A3	4.09	4.69	-1.52	0.0481
SLX1A-SULT1A3	4.12	4.73	-1.52	0.0112
SMIM8	5.04	5.65	-1.52	0.01
TBC1D31	3.62	4.22	-1.52	0.0211
U2AF1	7.61	8.21	-1.52	0.0028
WDR33	4.51	5.11	-1.52	0.0254
ZC3H6	4.05	4.65	-1.52	0.04
ZNF780A	4.05	4.65	-1.52	0.0359
AP1M1	5.65	6.26	-1.53	0.0156
CRKL	4.71	5.33	-1.53	0.0425
DEFB124	3.38	3.99	-1.53	0.0207
DENND3	3.72	4.34	-1.53	0.0112
FAM156A	4.21	4.82	-1.53	0.039
GNAQ	4.11	4.72	-1.53	0.0259

LGALS3	4.38	5	-1.53	0.0381
RPL23P2	4.82	5.43	-1.53	0.0161
ADH5P4	4.63	5.24	-1.54	0.0059
AK6P2	3.9	4.52	-1.54	0.0403
DICER1-AS1	5.42	6.04	-1.54	0.0094
DMGDH	3.41	4.04	-1.54	0.0074
F8A2	5.24	5.86	-1.54	0.0207
GABBR1	5.2	5.82	-1.54	0.0345
GRAMD3	4.41	5.04	-1.54	0.0388
KLF6	4.79	5.41	-1.54	0.0248
MFSD11	5.19	5.81	-1.54	0.0057
MST1P2	5.14	5.77	-1.54	0.0092
PEPD	4.84	5.46	-1.54	0.0114
SMARCE1	11.81	12.43	-1.54	0.0397
TMEM126A	4.75	5.38	-1.54	0.0079
TMOD4	4.14	4.77	-1.54	0.0451
FN3KRP	4.97	5.61	-1.55	0.0105
GPHN	5.39	6.03	-1.55	0.0474
LRP6	6.17	6.8	-1.55	0.0052
MICU2	5.39	6.02	-1.55	0.0093
NDFIP1P1	4.5	5.14	-1.55	0.0313
PHLPP1	4.85	5.48	-1.55	0.0454
RAP2B	3.48	4.12	-1.55	0.0433
SEPT14P8	6.27	6.9	-1.55	0.0408
USP36	5.33	5.97	-1.55	0.0129
ZNF91	5.7	6.33	-1.55	0.0257
ATP6V1C1	6.01	6.65	-1.56	0.0312
BNIP2	4.03	4.67	-1.56	0.0256
CDC42EP1	6.74	7.39	-1.56	0.0168
CPVL	6.01	6.65	-1.56	0.0371
IL15RA	4.15	4.79	-1.56	0.0074
PTMAP1	6.11	6.75	-1.56	0.0485
RAD18	4.38	5.02	-1.56	0.0202
RNU6-912P	6.89	7.53	-1.56	0.0225
STIP1	4.45	5.09	-1.56	0.0134
TMEM209	4.73	5.37	-1.56	0.0077
TMEM220	4.59	5.23	-1.56	0.0214
CHST9	3.54	4.2	-1.57	0.0026
FKBP1A	4.15	4.81	-1.57	0.0367
FUT6	4.08	4.72	-1.57	0.0164
GCNT3	6.68	7.33	-1.57	0.0433
GPNMB	4.53	5.18	-1.57	0.0289
MUC3A	4.52	5.17	-1.57	0.0362
MYO9B	4.61	5.26	-1.57	0.0358
RGPD3	4.4	5.05	-1.57	0.0313
SEP-08	3.67	4.32	-1.57	0.0342

STK4	6.12	6.77	-1.57	0.0309
TGS1	6.2	6.85	-1.57	0.024
WDR61	3.54	4.19	-1.57	0.0289
ADGRF5P2	3.85	4.52	-1.58	0.0284
COBLL1	4.91	5.58	-1.58	0.0352
COL5A3	4.94	5.6	-1.58	0.0488
MKRN1	4.36	5.02	-1.58	0.0107
PRODH2	3.74	4.4	-1.58	0.0307
TAF8	5.11	5.77	-1.58	0.0351
TRAF6	4.35	5.01	-1.58	0.003
ALKBH5	5.03	5.7	-1.59	0.0441
GUCD1	4.16	4.83	-1.59	0.0059
HCG4	3.57	4.23	-1.59	0.0038
HCP5	5.46	6.14	-1.59	0.023
HSPE1P12	4.33	5	-1.59	0.0218
KL	4.23	4.9	-1.59	0.0011
LAMTOR5	4.01	4.68	-1.59	0.0314
MIR548O2	4.34	5.01	-1.59	0.0346
NAA30	8.66	9.34	-1.59	0.027
NT5DC1	3.93	4.6	-1.59	0.0414
P2RY14	3.72	4.39	-1.59	0.0233
RGL3	3.6	4.28	-1.59	0.0375
RN7SL826P	4.61	5.28	-1.59	0.0029
SERPINA6	4.44	5.11	-1.59	0.0344
SNORD92	8.25	8.91	-1.59	0.0338
SNORD116-1	3.34	4.01	-1.59	0.0114
ZNF69	6.18	6.85	-1.59	0.0299
ZNF681	6.29	6.96	-1.59	0.0326
CEBPZOS	5.86	6.54	-1.6	0.0366
GGT5	4.36	5.04	-1.6	0.0035
MBTD1	3.95	4.62	-1.6	0.0146
MDH1	6.06	6.74	-1.6	0.0489
TARM1	6.37	7.05	-1.6	0.0031
USP39	7.08	7.76	-1.6	0.0307
ARHGAP28	4.16	4.85	-1.61	0.0173
DIP2B	5.07	5.76	-1.61	0.0245
ISYNA1	4.94	5.62	-1.61	0.003
KDELC2	4.09	4.78	-1.61	0.0121
NOP14-AS1	4.26	4.94	-1.61	0.0491
NRIP1	3.75	4.44	-1.61	0.0028
RBMS1P1	3.75	4.43	-1.61	0.0081
SCGB1D2	3.54	4.23	-1.61	0.0238
SLC6A13	4.27	4.95	-1.61	0.0191
SLC16A9	3.51	4.2	-1.61	0.0074
TRIM33	7.15	7.83	-1.61	0.002
WDR91	4.13	4.82	-1.61	0.0386

CTPS2	4.98	5.68	-1.62	0.0142
NOC3L	4.5	5.19	-1.62	0.0181
OR4C11	3.53	4.23	-1.62	0.0433
PM20D2	3.89	4.59	-1.62	0.0326
REXO2	5.32	6.01	-1.62	0.0303
RRAS2	5.78	6.48	-1.62	0.0432
RRM1	5.39	6.08	-1.62	0.0125
XRN2	4.38	5.08	-1.62	0.0331
ZMIZ1	5.66	6.35	-1.62	0.0457
AC055733.1	7.76	8.47	-1.63	0.049
ACTR3P2	4.75	5.45	-1.63	0.0117
C14orf166	3.82	4.53	-1.63	0.0245
CNKSR3	5.35	6.05	-1.63	0.0208
EFCAB14	5.08	5.78	-1.63	0.0125
KCNA4	3.49	4.19	-1.63	0.0026
MTCO3P22	4.33	5.04	-1.63	0.0073
PCBP1	6.24	6.94	-1.63	0.0041
PRDM1	5.87	6.57	-1.63	0.0061
SIDT2	3.99	4.69	-1.63	0.0436
STYX	4.91	5.61	-1.63	0.0201
TIMM9	3.76	4.46	-1.63	0.0153
TMEM27	3.87	4.57	-1.63	0.0497
ZZEF1	4.29	4.99	-1.63	0.0211
KRT38	3.84	4.55	-1.64	0.024
OSBPL10	4.71	5.43	-1.64	0.007
RPL34P21	4.73	5.44	-1.64	0.0424
RPS16P5	4.56	5.27	-1.64	0.0192
SLC40A1	5.31	6.03	-1.64	0.0087
SMYD2	4.41	5.12	-1.64	0.026
STON2	4.44	5.15	-1.64	0.048
COG3	7.42	8.15	-1.65	0.0213
KIAA1462	4.16	4.88	-1.65	0.0011
NFATC2IP	6.52	7.24	-1.65	0.017
NMNAT1	4.13	4.85	-1.65	0.0348
PARD3	4.48	5.21	-1.65	0.026
PMS2	4.68	5.41	-1.65	0.0425
PTGR1	5.42	6.13	-1.65	0.0234
RAPH1	4.36	5.08	-1.65	0.0048
RAPSN	3.74	4.46	-1.65	0.024
CCDC93	4.03	4.76	-1.66	0.0334
CFL2	3.57	4.3	-1.66	0.0163
MRPL48	5.28	6.01	-1.66	0.0064
RNU6V	5.74	6.47	-1.66	0.0187
SLC35A3	4.05	4.78	-1.66	0.0026
TPT1P5	5.5	6.23	-1.66	0.0009
UBE2D3P1	5.28	6.02	-1.66	0.0311

UTP18	5.45	6.17	-1.66	0.0349
YME1L1	4.79	5.51	-1.66	0.0295
ZNF141	6.01	6.74	-1.66	0.0039
ZNF480	4.85	5.58	-1.66	0.0023
BUB3	5.7	6.44	-1.67	0.0116
CHRNA10	4.68	5.42	-1.67	0.0107
MIR381	3.61	4.35	-1.67	0.0417
PNPLA4	3.82	4.56	-1.67	0.0284
PNRC2	7.9	8.64	-1.67	0.0469
SUSD6	6.01	6.74	-1.67	0.0223
TNS3	5.25	5.99	-1.67	0.0081
ZMYND11	4.66	5.4	-1.67	0.0364
DUSP1	3.55	4.3	-1.68	0.0161
H1F0	3.74	4.49	-1.68	0.0239
HMGB1P28	3.99	4.74	-1.68	0.0106
MAFIP	4.06	4.81	-1.68	0.0221
PSMA3	3.96	4.71	-1.68	0.0178
UQCR10	4.97	5.72	-1.68	0.0155
CD99P1	5.81	6.57	-1.69	0.023
CTNND1	5.78	6.55	-1.69	0.0185
CYP4V2	3.12	3.88	-1.69	0.0146
DYNC2H1	3.85	4.61	-1.69	0.0084
EXPH5	3.79	4.54	-1.69	0.0038
LEMD3	6.77	7.52	-1.69	0.0254
PPM1A	7.46	8.21	-1.69	0.0173
SMURF1	4.46	5.22	-1.69	0.0474
SOCS5	3.97	4.73	-1.69	0.0466
VTRNA2-1	4.73	5.49	-1.69	0.0104
AP000866.1	5.71	6.48	-1.7	0.0222
DAP3	6.72	7.49	-1.7	0.0107
NEK9	4.45	5.22	-1.7	0.0108
RCN2	4.39	5.16	-1.7	0.0137
RNU6-23P	7.67	8.44	-1.7	0.0085
RPS15AP32	3.26	4.03	-1.7	0.0242
RPS24P13	5.44	6.2	-1.7	0.0412
TBCEL	5.58	6.34	-1.7	0.0376
ANKRD17	4.39	5.16	-1.71	0.0403
GYPC	5.28	6.05	-1.71	0.0167
INTS3	4.74	5.52	-1.71	0.0116
MIR6759	6.56	7.33	-1.71	0.011
SEPT7P2	5.71	6.48	-1.71	0.0379
SLC6A12	5.56	6.34	-1.71	0.0096
UBIAD1	3.72	4.5	-1.71	0.0028
ASPN	4.19	4.98	-1.72	0.016
CLRN3	3.37	4.15	-1.72	0.0322
CYCSP24	4.24	5.01	-1.72	0.0145

LOC389834	3.47	4.25	-1.72	0.0471
LPIN3	3.71	4.5	-1.72	0.0325
MTND2P28	9.02	9.81	-1.72	0.0192
PIFO	3.81	4.59	-1.72	0.0051
PIGP	3.95	4.73	-1.72	0.0287
RNU6-29P	8.78	9.57	-1.72	0.0143
STAG3L2	6.77	7.55	-1.72	0.0242
ZNF98	5.62	6.4	-1.72	0.0046
ARL8B	5.61	6.39	-1.73	0.0121
CHRAC1	4.71	5.5	-1.73	0.0368
FTH1P3	4.56	5.35	-1.73	0.0139
NPTN	4.94	5.73	-1.73	0.0434
SCARNA16	3.66	4.45	-1.73	0.0034
VEZT	5.16	5.96	-1.73	0.0132
ADHFE1	3.63	4.43	-1.74	0.0081
EXOC5	4.85	5.65	-1.74	0.0027
HMG20A	6.24	7.04	-1.74	0.0154
KLHDC1	6.54	7.34	-1.74	0.0019
NDUFAF4	4	4.8	-1.74	0.0253
NPY1R	5.33	6.13	-1.74	0.0052
PEAK1	4.79	5.59	-1.74	0.0047
TMX2-CTNND1	4.5	5.29	-1.74	0.0233
ARHGEF28	5.17	5.98	-1.75	0.0028
ASAH1	7.89	8.69	-1.75	0.0028
CRIM1	6.09	6.9	-1.75	0.0409
EIF3F	5.04	5.85	-1.75	0.0103
PAN2	5.75	6.56	-1.75	0.0145
PRPSAP2	5.38	6.19	-1.75	0.012
THNSL1	6.03	6.83	-1.75	0.0199
TMEM128	5.05	5.86	-1.75	0.002
ARHGEF7	4.01	4.82	-1.76	0.0038
CWC27	4.99	5.81	-1.76	0.0192
PIK3CD	4.88	5.69	-1.76	0.02
PNPLA8	4.98	5.8	-1.76	0.0227
RNU6-839P	5.14	5.95	-1.76	0.0252
SBF2	5.58	6.4	-1.76	0.0292
WDR47	3.73	4.54	-1.76	0.0047
MIR548D1	4.77	5.59	-1.77	0.0259
PTGS2	3.58	4.41	-1.77	0.0462
RPS20P20	5.14	5.97	-1.77	0.0265
FAM3C	6.88	7.71	-1.78	0.0025
GOLIM4	4.32	5.15	-1.78	0.0229
KCTD18	5.13	5.96	-1.78	0.0148
KIAA0753	4.44	5.28	-1.78	0.0043
KLF9	4.37	5.2	-1.78	0.0009
RNU6-296P	4.24	5.08	-1.78	0.0022

RPL19	4.34	5.17	-1.78	0.018
SCARNA2	6.57	7.4	-1.78	0.0044
SPCS2	5.6	6.43	-1.78	0.0088
YBX3	4.29	5.12	-1.78	0.0174
CALCOCO2	8.63	9.47	-1.79	0.0187
CHMP4B	5.58	6.42	-1.79	0.0104
SCARNA27	4.29	5.13	-1.79	0.03
CCZ1B	6.37	7.22	-1.8	0.0435
LRRC37BP1	5.07	5.91	-1.8	0.0091
NMD3	4.98	5.83	-1.8	0.0199
BCL2L1	5.15	6.01	-1.81	0.0096
CLCN3P1	5.86	6.72	-1.81	0.0085
FAR1	5.39	6.24	-1.81	0.0229
MBD5	7.3	8.16	-1.81	0.0334
PPP2R5C	6.19	7.05	-1.81	0.0242
SPCS2	4.52	5.38	-1.81	0.0011
TEX30	4.4	5.26	-1.81	0.0096
RALGAPB	5.55	6.41	-1.82	0.0142
CLEC4A	4.09	4.96	-1.83	0.0208
GARNL3	5.99	6.86	-1.83	0.0005
PTBP2	3.4	4.28	-1.83	0.0248
SNHG11	5.48	6.36	-1.83	0.0349
SNORD116-2	4.5	5.37	-1.83	0.0393
AIFM3	5.28	6.16	-1.84	0.0043
ANKRD36B	5.76	6.64	-1.84	0.042
CCNYL1	5.46	6.34	-1.84	0.0357
RNU6-758P	8.31	9.19	-1.84	0.0411
SNORD11	5.11	5.99	-1.84	0.0377
ATP6V1A	6.65	7.53	-1.85	0.0298
LOC286297	4.05	4.94	-1.85	0.0296
PARVA	6.44	7.32	-1.85	0.0068
PMS2P3	6.05	6.94	-1.85	0.0187
RPL21P3	4.3	5.19	-1.85	0.0234
ACTR6	6.96	7.86	-1.86	0.0043
EIF2D	3.97	4.86	-1.86	0.0011
ESYT2	4.57	5.46	-1.86	0.0228
PEBP1	4.89	5.79	-1.86	0.0011
TXNRD2	6.34	7.23	-1.86	0.0304
BICC1	5.15	6.05	-1.87	0.049
HMGN2P5	7.78	8.68	-1.87	0.0031
HSPA1B	7.17	8.07	-1.87	0.0179
MAP1LC3B	7.08	7.99	-1.87	0.0338
RNU6-69P	5.8	6.7	-1.87	0.0156
ZDHHC13	5.98	6.88	-1.87	0.005
CDC42P3	6.93	7.84	-1.88	0.0052
CTAGE5	4.01	4.92	-1.88	0.0057

PDE8A	4.05	4.96	-1.88	0.0236
PRRG4	4.58	5.5	-1.88	0.0405
VPS45	4.68	5.59	-1.88	0.0334
AC006328.4	3.75	4.67	-1.89	0.0203
ACVR1	6.12	7.04	-1.89	0.0065
NR3C1	5.39	6.31	-1.89	0.0151
ZNF146	6.51	7.43	-1.89	0.0141
C14orf105	5.11	6.04	-1.9	0.0111
EIF4E2	6.42	7.34	-1.9	0.0092
FASTKD1	4.32	5.25	-1.9	0.0007
GPC4	3.65	4.58	-1.9	0.0065
MRPS36P1	4.98	5.9	-1.9	0.018
RNU6-554P	4.51	5.43	-1.9	0.0026
SMA4	4.94	5.86	-1.9	0.021
WDTC1	4.63	5.55	-1.9	0.0174
CEP104	4.93	5.86	-1.91	0.028
DBI	6.96	7.89	-1.91	0.041
FBXO7	5.34	6.27	-1.91	0.0038
GSR	6.17	7.1	-1.91	0.0248
ZMYM2	5.13	6.06	-1.91	0.0278
ZNF44	4.93	5.86	-1.91	0.0422
COX8A	3.59	4.53	-1.92	0.0025
DCTN3	3.78	4.72	-1.92	0.0203
FZD4	4.8	5.74	-1.92	0.0005
HNRNPH1	7.12	8.06	-1.92	0.01
PRCP	5.33	6.27	-1.92	0.0077
SUZ12P1	5.86	6.8	-1.92	0.0334
UGP2	7.51	8.45	-1.92	0.0109
EIF1P5	4.14	5.09	-1.93	0.0018
NBR2	6.22	7.17	-1.93	0.001
PPP1R3C	6.27	7.22	-1.93	0.0071
SLC25A30	4.51	5.46	-1.93	0.0312
SNRK	4.42	5.37	-1.93	0.0117
UBD	6.81	7.76	-1.93	0.0201
SEC24B	4.56	5.51	-1.94	0.0024
STK3	4.07	5.03	-1.94	0.027
TMEM38B	4.66	5.62	-1.94	0.016
ANKRD6	5.17	6.13	-1.95	0.0004
LRRC47	8.28	9.24	-1.95	0.0102
PRKAB1	5.36	6.32	-1.95	0.0035
CD36	4.04	5.01	-1.96	0.0469
DYNLL1P1	5.69	6.66	-1.96	0.0175
NAT8	6.77	7.75	-1.96	0.0181
NCOR1P1	6.28	7.25	-1.96	0.0419
SAP18	5.69	6.65	-1.96	0.0089
WBP1L	3.59	4.56	-1.96	0.019

AKAP10	5.08	6.06	-1.97	0.0035
BBS9	3.56	4.53	-1.97	0.0017
CHURC1-FNTB	5.38	6.36	-1.97	0.0145
GLUD1	4.55	5.52	-1.97	0.0073
HERPUD1	4.44	5.42	-1.97	0.0393
MRPL50	4.65	5.63	-1.97	0.0125
NFKBIA	4.34	5.32	-1.97	0.006
OR2A9P	4.5	5.48	-1.97	0.0377
XRCC6BP1	3.61	4.59	-1.97	0.0005
IQCK	5.59	6.57	-1.98	0.0236
LRP2	3.35	4.34	-1.98	0.0267
NREP	7.4	8.38	-1.98	0.0216
CHUK	5.53	6.53	-1.99	0.0449
DUSP16	5.78	6.77	-1.99	0.0208
FUS	6.51	7.5	-1.99	0.0001
LRRC23	4.04	5.04	-1.99	0.0055
PSMD13	7.63	8.62	-1.99	0.0371
UBBP1	5.95	6.94	-1.99	0.0347
CRIPAK	6.83	7.83	-2	0.0024
ELP3	4.18	5.18	-2	0.0494
HNRNPH2	5.47	6.47	-2	0.0185
IGIP	4.41	5.4	-2	0.0048
PPP1R15B	4.75	5.75	-2	0.0113
TAF9B	5.2	6.2	-2	0.0309
UMAD1	4.64	5.64	-2	0.0168
ACADVL	4.91	5.91	-2.01	0.0041
BIVM-ERCC5	3.37	4.38	-2.01	0.0013
C9orf85	4.25	5.26	-2.01	0.0012
MIR1277	5.45	6.46	-2.01	0.0283
NPIPB3	6.37	7.37	-2.01	0.0187
NTRK3	3.85	4.86	-2.01	0.0004
ATG14	5.14	6.15	-2.02	0.0118
CCDC34	8.14	9.15	-2.02	0.0277
LDHB	5.73	6.74	-2.02	0.0325
PIK3R3	4.14	5.16	-2.02	0.0012
PITPNB	5.56	6.58	-2.02	0.0265
SCAMP1	4.66	5.67	-2.02	0.0027
ATG4C	6.04	7.07	-2.03	0.0213
PMS2P2;	3.49	4.51	-2.03	0.0093
TMEM63A	5.74	6.76	-2.03	0.0158
TMEM135	4.17	5.2	-2.03	5.64E-05
CUBNP2	4.02	5.05	-2.04	0.0083
GATAD1	4.1	5.13	-2.04	0.0031
HMGN2	7.13	8.16	-2.04	0.0045
MBIP	5.4	6.43	-2.04	0.0158
NNT	7.62	8.65	-2.04	0.0183

ZNF136	5.13	6.16	-2.04	0.0025
BFAR	5.28	6.31	-2.05	0.0069
SGIP1	5.39	6.42	-2.05	0.0357
SMN1	8.61	9.65	-2.05	0.0381
SUPV3L1	6.13	7.17	-2.05	0.0338
ZFAND5	8.98	10.02	-2.05	0.0455
ZFP91-CNTF	4.75	5.79	-2.05	0.0082
HARS2	4.64	5.68	-2.06	0.0097
NOSTRIN	3.93	4.97	-2.06	0.0085
RAB11FIP2	4.86	5.9	-2.06	0.0003
CALCOCO1	6.52	7.58	-2.08	0.0132
LINC01021	4.83	5.88	-2.08	0.0425
SCARNA9	5.31	6.37	-2.08	0.0333
SLC39A8	6.39	7.45	-2.08	0.0451
STAM2	4.64	5.7	-2.08	0.0036
ARCN1	5.35	6.42	-2.09	0.0191
CEP350	6.09	7.16	-2.09	0.0281
EPHX1	4.84	5.9	-2.09	0.0246
LOC643802	4.66	5.72	-2.09	2.14E-05
DENND5B	4.26	5.33	-2.1	0.0227
FAM104B	5.02	6.09	-2.1	0.0044
NDUFA10	5.49	6.56	-2.1	0.011
RBMS2	4.13	5.2	-2.1	0.0043
CCDC6	3.72	4.79	-2.11	0.0421
CNNM2	3.76	4.83	-2.11	0.0257
EXOSC8	4.75	5.83	-2.11	0.0195
LCMT1	8.22	9.29	-2.11	0.0056
PAFAH1B2	8.42	9.49	-2.11	0.0142
RPS12P5	4.99	6.06	-2.11	0.0203
SCARNA5	10.05	11.13	-2.11	0.0396
ADGRF5	4.61	5.69	-2.12	0.0131
MTA3	5.38	6.47	-2.12	0.0084
PGAP1	4.94	6.02	-2.12	0.0157
PPFIA4	5.39	6.47	-2.12	0.0351
PPIG	7.54	8.63	-2.12	0.0267
RFC1	6.69	7.77	-2.12	0.0303
FOXJ3	5.25	6.34	-2.13	0.0322
TIMD4	4.43	5.52	-2.13	0.0213
PNRC2P1	6.99	8.09	-2.14	0.0324
PTTG1IP	4.04	5.14	-2.14	0.0084
RNU6-189P	6.46	7.57	-2.15	0.0133
SULT1C2	5.16	6.27	-2.15	0.0034
ACVR2A	5.76	6.87	-2.16	0.0306
BANP	4.41	5.52	-2.16	0.028
CCNY	4.63	5.73	-2.16	0.0104
FEM1C	3.97	5.08	-2.16	0.0049

MAPK9	4.64	5.76	-2.16	0.0095
PLEKHF2	5.17	6.28	-2.16	0.0213
PPP2R5E	4.49	5.6	-2.16	0.0335
RHOT1	4.24	5.35	-2.16	0.0156
DTWD1	6.47	7.59	-2.17	0.0448
NPY6R	4.34	5.46	-2.17	0.0017
PCBD1	3.5	4.62	-2.17	0.0051
TMEM176B	5.42	6.53	-2.17	0.005
MTND1P23	14.29	15.41	-2.18	0.0151
SPATA18	4.37	5.5	-2.18	0.0061
WDR45B	4.05	5.17	-2.18	0.0224
PTP4A2P2	9.43	10.56	-2.19	0.0301
SNX10	4.75	5.88	-2.19	0.0087
ACP1	4.73	5.87	-2.2	0.013
KRTCAP2	5.72	6.86	-2.2	0.0216
RNU6-533P	4.08	5.22	-2.2	0.0386
TMEM176A	5.84	6.98	-2.2	0.0088
GUSBP3	4.49	5.64	-2.21	0.0267
KMT5B	4.33	5.47	-2.21	0.0452
UBA2	4.95	6.1	-2.21	0.0108
EGLN1	9.08	10.23	-2.22	0.0401
MXI1	5.24	6.39	-2.22	0.0248
SLC47A1	4.33	5.48	-2.22	4.77E-05
ZNF827	4.23	5.37	-2.22	0.0023
HEATR5B	4.13	5.29	-2.23	0.0073
NBEAP1	3.58	4.73	-2.23	0.015
SPAG9	5.33	6.49	-2.23	0.0008
TRIM44	4.48	5.64	-2.23	0.0256
BMS1P4	3.94	5.11	-2.24	0.0092
C11orf1	5.03	6.19	-2.24	0.0074
MAR-07	4.56	5.73	-2.24	0.0196
FAM96A	5.6	6.77	-2.25	0.0301
SETD5	4.16	5.33	-2.25	0.0279
TSC22D3	5.15	6.33	-2.25	0.0357
TULP3	4.27	5.44	-2.25	0.031
ZFP91	5.51	6.69	-2.25	0.0015
ARL17B	5.14	6.32	-2.26	0.0456
HNF4G	3.86	5.04	-2.26	0.0041
NAP1L4	7.56	8.74	-2.26	0.0049
VEPH1	4.78	5.96	-2.26	0.0007
ZNF512	5.11	6.28	-2.26	0.0222
DDX5	8.67	9.86	-2.27	0.0028
PLEKHH1	4.29	5.47	-2.27	0.0468
BCAP29	5	6.19	-2.28	0.0137
MEAF6P1	4.9	6.09	-2.28	9.83E-05
TCTN1	3.85	5.04	-2.28	0.0046

HUS1	5.73	6.93	-2.29	0.0117
MYO6	4.68	5.88	-2.29	6.68E-05
SEPT7P5	4.33	5.53	-2.29	0.008
DYNC2LI1	5.92	7.12	-2.3	0.0213
GNPTAB	5.66	6.86	-2.3	0.0467
SDHB	3.96	5.15	-2.3	0.025
CD63	9.1	10.31	-2.31	0.026
CTR9	4.72	5.93	-2.31	0.0038
GRIA4	3.89	5.1	-2.31	0.0444
SPPL2A	3.91	5.11	-2.31	0.0008
CBWD1	7.12	8.34	-2.32	0.0354
LINC01057	5.15	6.36	-2.32	0.0172
NET1	6.19	7.4	-2.32	2.64E-05
NSRP1	4.66	5.87	-2.32	0.0143
SUOX	5.33	6.54	-2.32	0.0016
ZMAT1	4.62	5.84	-2.32	0.0243
CAMK2N1	8.16	9.38	-2.33	0.0261
SLC2A3P1	5.68	6.9	-2.33	0.0035
ZDHHC4	4.82	6.04	-2.33	0.0053
MIR548Z	5.06	6.29	-2.34	0.004
PITPNA	6.09	7.32	-2.34	0.0441
ARFIP1	3.73	4.96	-2.35	0.001
CMBL	5.21	6.44	-2.35	0.006
HDLBP	10.64	11.87	-2.35	0.0145
LMBRD1	6.25	7.48	-2.35	0.009
PICALM	9.95	11.19	-2.35	0.034
PPP1R37	3.68	4.92	-2.35	0.0282
SNX25	6.63	7.87	-2.35	0.0016
TUSC3	6.68	7.91	-2.35	0.0375
BNIP3L	7.93	9.17	-2.36	0.0314
DCAF16	4.07	5.31	-2.36	0.0054
PNMA1	5.04	6.28	-2.36	0.0039
POLK	4.89	6.13	-2.36	0.004
PPIL4	8.1	9.34	-2.36	0.0386
TRMT13	7.14	8.38	-2.36	0.0251
SERINC1	5.95	7.19	-2.37	0.0397
TMEM261	5.12	6.36	-2.37	0.0177
CYP51A1	5.31	6.56	-2.38	0.0024
HBA2	5.39	6.65	-2.38	0.0235
NT5C3B	5.92	7.17	-2.38	0.0352
CAST	5.78	7.04	-2.39	0.0227
MIR548F3	4.52	5.77	-2.39	0.0093
RNU6-1313P	4.3	5.56	-2.39	0.0382
SMG1P5	6.13	7.39	-2.39	0.0005
TDRD3	5.18	6.43	-2.39	0.0014
ZNF493	5.22	6.47	-2.39	0.0186

HNRNPA1P44	5.32	6.58	-2.4	0.0239
PARP6	7.48	8.74	-2.4	0.0337
PRR13	5.85	7.12	-2.4	0.0123
RSU1P3	3.48	4.75	-2.4	0.0012
TNIP1	4.78	6.04	-2.4	0.0111
UGGT2	4.47	5.74	-2.4	0.0028
SMG1P2	7.29	8.56	-2.41	0.0064
STAG2	5.34	6.62	-2.41	0.0397
USP9X	5.26	6.53	-2.41	0.0203
ZBTB25	4.83	6.1	-2.41	0.0053
LSM14A	8.69	9.97	-2.42	0.0231
FAM3C2	6.01	7.29	-2.43	0.0003
MPC2	4.68	5.96	-2.43	0.0489
SLC25A16	4.42	5.7	-2.43	0.0002
APLP2	4.7	5.98	-2.44	0.0116
KAT6B	4.35	5.64	-2.44	0.0004
KIF21A	4.03	5.32	-2.44	0.018
STT3B	5.68	6.97	-2.44	0.0233
PIAS1	4.83	6.12	-2.45	0.0369
PNP	6.31	7.61	-2.45	0.0005
TAS2R15P	5.35	6.64	-2.45	0.0009
GNG2	5.18	6.48	-2.46	0.0057
HNRNPA1P19	4.94	6.24	-2.46	0.0194
IFT52	5.59	6.9	-2.47	0.0381
RAB18	4.43	5.74	-2.47	0.0001
WASL	5.57	6.88	-2.47	0.0037
ZNF675	8.04	9.35	-2.47	0.0237
BAZ2B	4.61	5.92	-2.48	0.0016
CYP3A5	4.37	5.67	-2.48	0.0009
EEF2	6.89	8.2	-2.48	0.0451
MRPS36	7.63	8.94	-2.48	0.0381
RNU6-96P	4.65	5.97	-2.48	0.0155
RPL36AL	8.02	9.33	-2.48	0.007
ZFAND3	4.75	6.06	-2.48	0.0008
CRYAB	6.82	8.14	-2.49	0.0422
CSDE1	7.53	8.85	-2.49	0.0014
LRRK2	6.76	8.08	-2.49	0.0018
CLK4	5.78	7.11	-2.5	0.0108
ENTPD5	4.94	6.26	-2.5	0.0003
PPP1R11	5.9	7.22	-2.5	0.0035
SEMA6A	4.52	5.84	-2.5	0.0003
PAPOLA	5.95	7.28	-2.51	0.049
PDCL3P4	6.7	8.03	-2.51	0.0013
MS4A4E	4.1	5.43	-2.52	0.0086
SLC15A4	6.96	8.29	-2.52	0.0051
C12orf29	7.5	8.84	-2.53	0.0047

MTUS1	11.85	13.19	-2.53	0.0486
CYB5A	4.39	5.73	-2.54	0.0388
NT5C2	5.02	6.36	-2.54	0.0021
VBP1	4.61	5.95	-2.54	0.0027
DDX17	9.06	10.41	-2.55	0.0241
LRP6	4.97	6.32	-2.55	0.0046
AP3B1	4.51	5.87	-2.56	0.0216
TLR3	4.8	6.15	-2.56	0.0311
AGAP9	5.37	6.73	-2.57	0.0199
ASPA	4.23	5.59	-2.57	0.0338
CBX7	7.3	8.66	-2.57	0.0081
CUL2	4.03	5.39	-2.58	0.0447
ESM1	5.66	7.03	-2.58	0.0124
JMJD1C	5.36	6.72	-2.58	0.0191
ZBTB44	3.42	4.78	-2.58	0.0122
ASCC3	3.92	5.29	-2.59	0.0035
DNAJB2	4.43	5.8	-2.59	0.0029
PCF11	5.35	6.72	-2.59	0.0002
ACAD11	3.82	5.2	-2.6	0.005
FRG1HP	6.36	7.74	-2.6	0.0003
IFNGR2	6.16	7.54	-2.6	0.0076
SERPINA1	3.77	5.14	-2.6	0.0059
ETV3	3.56	4.95	-2.61	0.0036
KANSL3	3.65	5.03	-2.61	0.0116
SLC2A5	4.42	5.8	-2.61	0.0173
SAV1	4.69	6.08	-2.62	0.0056
CSTB	9.47	10.86	-2.63	0.022
DAB2	6.66	8.06	-2.63	0.034
TRAPPC4	4.9	6.3	-2.63	0.0323
G3BP2	7.63	9.03	-2.64	0.0316
WHAMMP3	4.54	5.93	-2.64	0.032
FAM120AOS	4.96	6.37	-2.65	0.0006
EMCN	5.53	6.94	-2.66	0.0498
ERICH1	4.72	6.13	-2.66	0.0009
MKLN1	6.58	7.99	-2.66	0.0401
ZNF207	3.85	5.26	-2.66	0.0049
CRYL1	4.92	6.33	-2.67	0.0058
DPY19L4	5.75	7.18	-2.68	0.0163
TOM1L1	5.96	7.38	-2.68	0.0016
ATXN2L	5.29	6.72	-2.7	0.0049
BEND7	5.48	6.91	-2.7	0.0001
C11orf73	5.92	7.36	-2.7	0.0102
REREP2Y	5.85	7.28	-2.7	0.003
POLR1D	9.45	10.89	-2.71	0.0208
GLRX	6.92	8.37	-2.72	0.0305
CDK17	5.49	6.94	-2.73	0.0037

PKP4	6.03	7.48	-2.73	0.0161
RPS24	11.09	12.54	-2.73	0.04
ANKRD36C	6.61	8.07	-2.75	0.0492
NDUFA9	3.92	5.38	-2.75	0.0084
UBC	8.15	9.62	-2.76	0.0102
DCTN4	8.15	9.63	-2.8	0.0148
MIR548H3	5.12	6.61	-2.8	0.0331
MTCYBP3	3.81	5.29	-2.8	6.84E-07
FAM133CP	4.72	6.22	-2.83	0.0264
FLT1	3.45	4.95	-2.83	0.0253
WRB	6.6	8.1	-2.83	0.0207
PRPF3	7.05	8.56	-2.84	0.0094
ADAM22	4.22	5.73	-2.86	0.0158
ERV3-1	7.75	9.27	-2.88	0.001
GON4L	5.16	6.69	-2.88	0.0322
HSDL2	5.1	6.63	-2.89	0.003
RNF2	4.72	6.25	-2.89	0.0388
RRAGD	5.37	6.91	-2.9	0.0076
STK38L	8.24	9.78	-2.91	0.0333
CHURC1	9.07	10.62	-2.93	0.0038
CUL5	6.25	7.8	-2.93	0.0056
NPEPPS	5.19	6.75	-2.93	0.001
EVL	4.87	6.43	-2.94	0.0001
KANSL1L	4.03	5.59	-2.94	0.0001
CSF1	4.76	6.33	-2.97	0.0251
DKC1MIR664B	5.55	7.12	-2.97	0.0027
RNASEKC17orf49	10.54	12.1	-2.97	0.0239
CSNK2A1	12.17	13.75	-2.98	0.0442
UBE2G2	7.17	8.75	-2.98	0.0032
ROCK2	6.18	7.76	-2.99	0.0077
RP11-231C14.4	6.22	7.8	-2.99	0.0331
AC017081.1	14.68	16.29	-3.05	0.0142
RCAN2	3.65	5.25	-3.05	0.0006
PTPRM	5.23	6.84	-3.06	0.0077
SEP-11	7.19	8.81	-3.06	0.0082
TAOK1	6.92	8.54	-3.06	0.0237
LUC7L3	4.92	6.55	-3.08	0.0245
RPS4X	7.67	9.29	-3.08	0.0358
TSC22D1	7.83	9.46	-3.08	0.0033
FRG1JP	7.41	9.05	-3.11	0.0055
APOC1P1	5.36	7	-3.12	0.0051
TMEM246	5.38	7.03	-3.12	0.0039
UBN2	7.94	9.58	-3.13	0.0164
LRP6	5.16	6.81	-3.14	0.0009
NUCB2	6.25	7.91	-3.16	0.0047
ERBB3	6.04	7.71	-3.17	0.0099

MCFD2	5.4	7.06	-3.17	0.0058
MTHFD1	5.02	6.69	-3.18	0.0286
HNRNPUP1	7.61	9.28	-3.19	0.0084
RBPMS	9.33	11.01	-3.21	0.0461
GLYAT	4.13	5.83	-3.24	0.0063
UBE2V1P1	5.25	6.94	-3.24	0.0331
CCSER2	5.55	7.25	-3.25	0.0146
ARFGEF2	9.47	11.18	-3.27	0.0115
COX20P1	8.14	9.85	-3.27	0.0427
HIPK2	7.41	9.12	-3.27	0.0193
IDH3A	6.82	8.53	-3.27	0.0021
LPGAT1	5.06	6.78	-3.28	0.0011
RMND5A	4.84	6.56	-3.28	0.0042
ATM	5.58	7.3	-3.29	0.0325
RFWD2	3.62	5.33	-3.29	0.0057
TXNIP	7.32	9.04	-3.29	0.0078
ZC3H14	5.54	7.26	-3.29	0.004
ACTR10	6.55	8.27	-3.3	0.0173
AGMO	3.79	5.52	-3.3	0.0082
RNU6-1091P	4.52	6.24	-3.31	0.001
MGAT4A	4.93	6.66	-3.32	0.0278
TSG101	5.29	7.02	-3.32	0.0258
MIR548D2	8.35	10.09	-3.33	0.0148
MNAT1	6.19	7.92	-3.33	0.0037
FARP1	5.47	7.21	-3.34	0.0247
CSRP2	5.83	7.58	-3.37	0.0023
SUZ12P1	6.9	8.66	-3.38	0.0027
FAF2	5.86	7.62	-3.39	0.007
NPIPB5	8.16	9.93	-3.41	0.0014
RNU6-1322P	5.74	7.52	-3.42	0.0122
DDX18	7.44	9.26	-3.52	0.0023
RNU6-28P	10.41	12.23	-3.53	0.0101
DYNLRB1	6.61	8.44	-3.55	0.0011
UQCRC2	5.62	7.45	-3.55	0.0075
VMP1	13.88	15.73	-3.6	0.0494
ZEB1	6.24	8.1	-3.63	0.0036
PPP1R3E	5.48	7.36	-3.67	9.00E-05
RPL23A	14.18	16.05	-3.67	0.0372
ARHGAP42	4.89	6.78	-3.69	5.36E-05
SMIM19	5.64	7.53	-3.7	0.0057
NACA	12.96	14.86	-3.72	0.0158
RBMX	7.16	9.06	-3.74	0.0053
CAST	16.09	18	-3.75	0.0209
RNU6-797P	5.67	7.58	-3.76	0.0089
CCZ1	7.12	9.04	-3.78	0.0327
C11orf54	6.78	8.71	-3.81	0.0149

ZNF43	7.37	9.32	-3.86	0.0049
AMZ2	8.06	10.01	-3.87	0.0318
LRBA	6.49	8.45	-3.88	0.0037
GKAP1	5.48	7.44	-3.89	9.29E-05
CYP2J2	4.78	6.75	-3.92	0.0005
RIMKLB	4.72	6.7	-3.92	2.26E-06
FOXN3	8.6	10.59	-3.97	0.0027
RDX	4.11	6.1	-3.98	0.0034
MFF	6.35	8.37	-4.03	0.0042
AK3	6.59	8.61	-4.07	0.0051
GPR160	6.35	8.38	-4.08	0.0055
TBL1XR1	4.71	6.74	-4.08	0.0078
POR	5.85	7.89	-4.09	0.0019
ND2	11.53	13.58	-4.13	0.0026
SEPP1	5.02	7.07	-4.13	0.0043
ANK3	5.32	7.36	-4.14	0.0021
MGST1	9.09	11.15	-4.17	0.0315
ACE2	3.34	5.4	-4.19	0.0001
PGRMC2	7.6	9.69	-4.23	0.0003
SLC3A1	5.38	7.49	-4.33	0.0076
THADA	5.61	7.73	-4.35	0.0494
ACAT1	8.16	10.28	-4.36	0.0108
ZNF100	7.54	9.67	-4.38	0.0104
SLC17A3	3.85	5.99	-4.4	0.0137
MAP4K3	4.07	6.22	-4.42	0.0001
METTL10	6.55	8.7	-4.42	0.041
RGPD6	6.33	8.48	-4.43	0.0016
RGPD8	6.85	9.01	-4.46	0.0009
AKAP8L	6.77	8.94	-4.51	0.0132
HILPDA	5.6	7.77	-4.52	0.0198
EMX2	4.76	6.94	-4.54	0.0036
FNIP2	4.69	6.88	-4.55	0.0053
CCNI	10.63	12.83	-4.6	0.0237
RNU6-34P	7.49	9.7	-4.64	0.003
SNORD50A	9.54	11.82	-4.87	0.0158
SNX2	8.87	11.17	-4.92	0.0389
MRPS30	5.8	8.1	-4.93	0.0034
SETD3	6.33	8.64	-4.98	3.18E-05
PLEKHA1	4.2	6.61	-5.3	0.0015
DMXL1	5.54	8.01	-5.53	0.0026
YTHDC1	4.26	6.74	-5.56	0.0172
RNU6-428P	5.76	8.24	-5.58	0.003
MIR548AJ2	6.8	9.29	-5.61	0.0021
RALGAPA1	6.07	8.6	-5.77	4.17E-05
GOT1	3.82	6.37	-5.86	0.0005
ARHGEF12	7.03	9.6	-5.97	0.0048

GABARAPL1	7.72	10.32	-6.05	0.0078
NCOR1	7.7	10.29	-6.05	0.0003
MED14	5.69	8.31	-6.16	0.0059
CLDN10	6.98	9.62	-6.22	0.0038
APOC1	11.44	14.11	-6.35	0.0077
ZDHHC7	5.27	8.01	-6.68	0.0434
PRRC2C	10.73	13.5	-6.84	0.0408
CUBN	3.8	6.6	-6.97	0.0002
USMG5	6.45	9.37	-7.55	0.0202
MIR4454	9.77	12.71	-7.67	0.0192
PLPP3	7.41	10.37	-7.76	0.0015
BHLHE41	8.66	11.66	-7.98	0.0015
WBP11	7.52	10.68	-8.93	0.0017
UTRN	7.88	11.19	-9.92	0.0028
PDK4	5.81	9.26	-10.95	0.0155
RNU6-267P	4.4	8.3	-14.93	0.0213
RNU6-162P	4.98	8.95	-15.7	0.0304
N4BP2L2	13.92	18.08	-17.91	0.0132
SECISBP2L	6.37	12.19	-56.48	0.0002

Supplementary Table S7. ROC analysis of the identified DEmiRNAs and DEgenes between sunitinib responders and non-responders.

MiRNA/genes	**AUC (95% CI)**	***P*-value**
Single miRNAs and single genes		
miR-223	0.73 (0.55-0.86)	0.0102
miR-155	0.67 (0.49-0.82)	0.0727
miR-200b	0.66 (0.48- 0.81)	0.1031
miR-130b	0.59 (0.38- 0.78)	0.477
PRDM1	0.77 (0.58-0.90)	0.0066
FLT1	0.83 (0.66-0.93)	<0.0001
SAV1	0.83 (0.71-0.97)	<0.0001
Combinations		
All miRNAs	0.7 (0.49-0.86)	0.0735
miR-223 + miR-155 + miR-200b	0.71 (0.53-0.85)	0.0254
miR-223 + miR-155	0.71 (0.53-0.85)	0.0243
miR-223 + miR-200b	0.7 (0.52-0.85)	0.0321
miR-155 + miR-200b	0.67 (0.48-0.82)	0.0975
miR-130b + miR-155	0.62 (0.41-0.8)	0.36
miR-130b + miR-223	0.6 (0.41-0.8)	0.3
miR-130b + miR200b	0.6 (0.39-0.78)	0.4
miR-130b + miR-155+ miR-200b	0.58 (0.38-0.77)	0.52
miR-130b + miR-155 +	0.71 (0.5-0.87)	0.05
miR-223	0.66 (0.45- 0.83)	0.15
miR-130b + miR-200b+ miR-223		
All genes	0.9 (0.72-0.98)	<0.0001
PRDM1 + FLT1	0.83 (0.64-0.94)	<0.0001
PRDM1 + SAV1	0.86 (0.67-0.96)	<0.0001
FLT1 + SAV1	0.89 (0.71-0.97)	<0.0001
miR-223 + PRDM1	0.86 (0.68-0.96)	<0.0001
miR-200b + FLT1	0.81 (0.64-0.92)	0.0002
miR-223 + FLT1	0.83 (0.67-0.94)	<0.0001
miR-223 + SAV1	0.92 (0.76-0.99)	<0.0001
miR-200b + PRDM1	0.86 (0.68-0.96)	<0.0001
miR-200b + SAV1	0.9 (0.73-0.98)	<0.0001
miR-155 + PRDM1	0.83 (0.64-0.94)	<0.0001
miR-155 + FLT1	0.83 (0.66-0.94)	<0.0001
miR-155 + SAV1	0.90 (0.73-0.98)	<0.0001
miR-130b + SAV1	0.89 (0.68-0.98)	<0.0001
miR-130b + FLT1	0.76 (0.55-0.9)	0.0145
miR-130b + PRDM1	0.76 (0.53-0.92)	0.0377

AUC: Area under the curve, CI: Confidence interval.
